# Short-term Outcomes of Laparoscopy-Assisted vs Open Surgery for Patients With Low Rectal Cancer: The LASRE Randomized Clinical Trial

**DOI:** 10.1001/jamaoncol.2022.4079

**Published:** 2022-09-15

**Authors:** Wei-Zhong Jiang, Jian-Min Xu, Jia-Di Xing, Hui-Zhong Qiu, Zi-Qiang Wang, Liang Kang, Hai-Jun Deng, Wei-Ping Chen, Qing-Tong Zhang, Xiao-Hui Du, Chun-Kang Yang, Yin-Cong Guo, Ming Zhong, Kai Ye, Jun You, Dong-Bo Xu, Xin-Xiang Li, Zhi-Guo Xiong, Kai-Xiong Tao, Ke-Feng Ding, Wei-Dong Zang, Yong Feng, Zhi-Zhong Pan, Ai-Wen Wu, Feng Huang, Ying Huang, Ye Wei, Xiang-Qian Su, Pan Chi

**Affiliations:** 1 Department of Colorectal Surgery, Fujian Medical University Union Hospital, Fuzhou, China; 2 Department of General Surgery, Zhongshan Hospital, Fudan University, Shanghai, China; 3 Key Laboratory of Carcinogenesis and Translational Research, Ministry of Education, Gastrointestinal Cancer Center, Peking University Cancer Hospital and Institute, Beijing, China; 4 Division of Colorectal Surgery, Department of General Surgery, Peking Union Medical College Hospital, Chinese Academy of Medical Sciences & Peking Union Medical College, Beijing, China; 5 Department of Gastrointestinal Surgery, West China Hospital, Sichuan University, Chengdu, China; 6 Department of Colorectal Surgery, The Sixth Affiliated Hospital, Sun Yat-sen University, Guangzhou, China; 7 Department of General Surgery, Nanfang Hospital, Southern Medical University, Guangzhou, China; 8 Department of Colorectal Surgery, Cancer Hospital of the University of Chinese Academy of Sciences & Zhejiang Cancer Hospital, Hangzhou, China; 9 Department of Colorectal Surgery, Cancer Hospital of China Medical University, Liaoning Cancer Hospital & Institute, Shenyang, China; 10 Department of General Surgery, General Hospital of PLA, Beijing, China; 11 Department of Gastrointestinal Oncological Surgery, Fujian Provincial Cancer Hospital, Fuzhou, China; 12 Department of Colorectal & Anal Surgery, Zhangzhou Affiliated Hospital, Fujian Medical University, Zhangzhou, China; 13 Department of Gastrointestinal Surgery, Renji Hospital, Shanghai Jiao Tong University School of Medicine, Shanghai, China; 14 Department of Gastrointestinal Surgery, The Second Affiliated Hospital, Fujian Medical University, Quanzhou, China; 15 Department of Gastrointestinal Oncological Surgery, The First Affiliated Hospital, Xiamen University, Xiamen, China; 16 Department of Colorectal & Anal Surgery, Longyan Affiliated Hospital, Fujian Medical University, Longyan, China; 17 Department of Colorectal Surgery, Fudan University Cancer Center, Shanghai, China; 18 Department of Gastrointestinal Surgery, Hubei Provincial Cancer Hospital, Wuhan, China; 19 Department of Gastrointestinal Surgery, Union Hospital, Tongji Medical College, Huazhong University of Science and Technology, Wuhan, China; 20 Department of Colorectal Surgery and Oncology, Key Laboratory of Cancer Prevention and Intervention, Ministry of Education, The Second Affiliated Hospital, School of Medicine, Zhejiang University, Hangzhou, China; 21 Department of Colorectal Oncological Surgery, Shengjing Hospital, China Medical University, Shenyang, China; 22 Department of Colorectal Surgery, Sun Yat-sen University Cancer Center, Guangzhou, China

## Abstract

**Importance:**

The efficacy of laparoscopic vs open surgery for patients with low rectal cancer has not been established.

**Objective:**

To compare the short-term efficacy of laparoscopic surgery vs open surgery for treatment of low rectal cancer.

**Design, Setting, and Participants:**

This multicenter, noninferiority randomized clinical trial was conducted in 22 tertiary hospitals across China. Patients scheduled for curative-intent resection of low rectal cancer were randomized at a 2:1 ratio to undergo laparoscopic or open surgery. Between November 2013 and June 2018, 1070 patients were randomized to laparoscopic (n = 712) or open (n = 358) surgery. The planned follow-up was 5 years. Data analysis was performed from April 2021 to March 2022.

**Interventions:**

Eligible patients were randomized to receive either laparoscopic or open surgery.

**Main Outcomes and Measures:**

The short-term outcomes included pathologic outcomes, surgical outcomes, postoperative recovery, and 30-day postoperative complications and mortality.

**Results:**

A total of 1039 patients (685 in laparoscopic and 354 in open surgery) were included in the modified intention-to-treat analysis (median [range] age, 57 [20-75] years; 620 men [59.7%]; clinical TNM stage II/III disease in 659 patients). The rate of complete mesorectal excision was 85.3% (521 of 685) in the laparoscopic group vs 85.8% (266 of 354) in the open group (difference, −0.5%; 95% CI, −5.1% to 4.5%; *P* = .78). The rate of negative circumferential and distal resection margins was 98.2% (673 of 685) vs 99.7% (353 of 354) (difference, −1.5%; 95% CI, −2.8% to 0.0%; *P* = .09) and 99.4% (681 of 685) vs 100% (354 of 354) (difference, −0.6%; 95% CI, −1.5% to 0.5%; *P* = .36), respectively. The median number of retrieved lymph nodes was 13.0 vs 12.0 (difference, 1.0; 95% CI, 0.1-1.9; *P* = .39). The laparoscopic group had a higher rate of sphincter preservation (491 of 685 [71.7%] vs 230 of 354 [65.0%]; difference, 6.7%; 95% CI, 0.8%-12.8%; *P* = .03) and shorter duration of hospitalization (8.0 vs 9.0 days; difference, −1.0; 95% CI, −1.7 to −0.3; *P* = .008). There was no significant difference in postoperative complications rate between the 2 groups (89 of 685 [13.0%] vs 61 of 354 [17.2%]; difference, −4.2%; 95% CI, −9.1% to −0.3%; *P* = .07). No patient died within 30 days.

**Conclusions and Relevance:**

In this randomized clinical trial of patients with low rectal cancer, laparoscopic surgery performed by experienced surgeons was shown to provide pathologic outcomes comparable to open surgery, with a higher sphincter preservation rate and favorable postoperative recovery.

**Trial Registration:**

ClinicalTrials.gov Identifier: NCT01899547

## Key Points

**Question:**

Is laparoscopic surgery safe for treatment of low rectal cancer in terms of short-term oncologic outcomes?

**Findings:**

In this randomized clinical trial including 1039 patients, laparoscopic surgery had a comparable rate of complete total mesorectal excision (85.3% vs 85.8% in open surgery). The rate of negative circumferential and distal resection margins was 98.2% vs 99.7% and 99.4% vs 100%, respectively, and the median number of lymph nodes retrieved was 13.0 vs 12.0, respectively; laparoscopic surgery had a higher sphincter preservation rate and favorable postoperative recovery.

**Meaning:**

Compared with open surgery, laparoscopic surgery was shown to be safe for treatment of low rectal cancer in terms of short-term oncologic outcomes.

## Teaser

This randomized clinical trial compares the short-term efficacy of laparoscopic surgery vs open surgery for treatment of low rectal cancer.

## Introduction

Total mesorectal excision (TME) is the cornerstone of rectal cancer surgery.^1^ In recent decades, laparoscopic surgery has been increasingly performed to treat rectal cancer. However, it remains challenging to achieve oncological outcomes equivalent to open surgery, particularly for low rectal cancer. Low-lying rectal cancer in narrow pelvic spaces increases the difficulty of sharp dissection in laparoscopic surgery and could jeopardize surgical quality.^2,3,4^

Surgical quality indicators, such as TME quality, negative circumferential resection margins (CRMs), negative distal resection margins (DRMs), and number of retrieved lymph nodes, are surrogate markers of local rectal cancer recurrence.^2,3,4^ Previous randomized clinical trials comparing laparoscopic and open surgeries for rectal cancer have yielded conflicting results.^5,6,7,8,9^ The MRC CLASICC trial^5^ showed similar rates of negative CRM between laparoscopic and open surgeries. The COREAN^6^ and COLOR II^7^ trials reported comparable rates of high-quality TME in addition to similar rates of negative CRM. However, the ACOSOG Z6051^8^ and ALaCaRT trials^9^ failed to demonstrate noninferiority of the composite “successful resection” outcome (complete or nearly complete TME, negative CRMs and DRMs) in the laparoscopic group. Nevertheless, given the small sample sizes in these trials, little is known about the surgical quality of laparoscopic vs open surgery for low rectal cancer.

Two retrospective cohort studies have suggested equivalent perioperative safety and long-term outcomes for laparoscopic and open surgeries for low rectal cancer.^10,11^ Given the scarcity of results regarding surgical quality, evidence supporting the use of laparoscopic surgery for low rectal cancer remains insufficient. Thus, a large-scale randomized clinical trial of laparoscopic surgery for low rectal cancer was warranted.

The Laparoscopy-Assisted Surgery for Carcinoma of the Low Rectum (LASRE) trial was designed to compare the oncological outcomes between laparoscopic and open surgeries for low rectal cancer. We herein report the short-term pathologic and surgical outcomes. The primary end point measure of the 3-year disease-free survival (DFS) rate will be reported later when data become available.

## Methods

### Study Design and Participants

The LASRE trial is a multicenter, noninferiority randomized clinical trial conducted in 22 tertiary hospitals across China. The inclusion and exclusion criteria are shown in eTable 1 in Supplement 1. The trial protocol was approved by the central ethics committees of Fujian Medical University Union Hospital and local ethics committees of all the other participating centers. All participants provided written informed consent before enrollment. The trial protocol is presented in Supplement 2. This trial followed the Consolidated Standards of Reporting Trials (CONSORT) reporting guideline.

### Hospital and Surgeon Eligibility

Hospitals performing more than 30 laparoscopic TME surgeries per year were invited to participate. The participating surgeons were required to have performed at least 100 laparoscopic TME surgeries and submitted at least 2 unedited, anonymous videos of laparoscopic TME procedures for review to an Academic Credentialing Committee. Finally, 29 surgeons from 22 centers were qualified for participation.

### Randomization and Masking

Eligible patients were randomized (2:1) to undergo laparoscopic or open surgery using a dynamic minimization algorithm. Randomization was conducted on the day prior to the surgery using a central distributed annotation system for an interactive web response system and was stratified by clinical tumor stage (stage I or II/III), age (≤44 years, 45-59 years, or ≥60 years), sex, body mass index (BMI, calculated as weight in kilograms divided by height in meters squared; ≤23.9, 24.0-27.9, or ≥28.0), and American Society of Anesthesiologists classification (I, II, or III). The investigators and patients were not blinded to the treatment allocation.

### Interventions

#### Preoperative Chemoradiotherapy

Preoperative chemoradiotherapy was recommended for patients with clinical stage II/III disease in accordance with the National Comprehensive Cancer Network guidelines (version 4.2013).^12^ Radiotherapy was performed with a total dose of 45 to 50.4 Gy in 25 or 28 fractions. Concurrent chemotherapy was based on fluorouracil or its analogs. According to the Academic Advisory Committee recommendations, patients received additional preoperative capecitabine chemotherapy (1250 mg/m^2^, twice a day for 14 days) after radiotherapy and before surgery. Surgery was performed 6 to 8 weeks after radiotherapy in accordance with the National Comprehensive Cancer Network guidelines.^12^

#### Surgery

Both laparoscopic and open surgeries were performed according to the principles of TME. The central lymph nodes were dissected, irrespective of high or low inferior mesenteric artery ligation. The sharp pelvic dissection was performed along the surgical plane between the mesorectal and parietal fascia, and the autonomic nerves were preserved. The DRM was at least 1 and 2 cm for patients with and without preoperative chemoradiotherapy, respectively. The proximal resection margin was at least 10 cm from the upper tumor edge. The decision for sphincter preservation was jointly made by the attending surgeon and the patient. Splenic flexure mobilization was performed after considering the intraoperative circumstances. For sphincter-preserving surgery, diverting ileostomy or colostomy was performed in patients with high-risk anastomosis.

#### Pathologic Assessments

After a macroscopic assessment of TME quality by the operating surgeon, resected specimens were photographed for a central TME quality review by 2 experienced pathologists (Yuan-E Lian and Hu Chen) blinded to the patient information, surgical approach, and identities of operating surgeons. The TME quality was graded using the criteria of Nagtegaal et al^2^ as complete, nearly complete, or incomplete. Microscopic pathological assessment was performed by local pathologists at the participating centers. A positive resection margin, including CRM or DRM, was defined by the presence of cancer cells within 1 mm of the cut edge.

### Outcome Measures

Herein, we evaluated the short-term outcomes, including pathologic, surgical, and postoperative outcomes. Pathologic outcomes included the TME quality, negative CRM, negative DRM, lengths of proximal resection margin and DRM, and the number of retrieved lymph nodes. Surgical outcomes were defined as intraoperative events and surgery-related details, such as conversion rate, operating time, estimated blood loss, intraoperative complications, surgery type, and diverting ostomy creation. Postoperative outcomes included the characteristics of postoperative recovery, any complications occurring within 30 days, duration of hospitalization, and 30-day mortality. Complications were graded using the Clavien-Dindo classification^13^; grades III to V indicated severe complications.

### Statistical Analysis

The sample size was estimated using a log-rank test based on 3-year DFS with a noninferiority margin of 10%. When designing this trial, a noninferiority margin of 10% was determined to be clinically acceptable.^3,5^ The sample size was separately calculated for stage I vs II/III disease, considering distinct prognoses. Assuming that the 3-year DFS rates in open surgery were 94.3% and 75.2% for clinical stage I and II/III diseases, respectively,^14,15^ 359 patients with stage I disease (laparoscopic, 240; open, 119) and 609 with stage II/III disease (laparoscopic, 406; open, 203) and a total of 968 patients (laparoscopic, 646; open, 322) were required to provide 80% power with a 1-sided α of 2.5%. Accordingly, 1065 patients were enrolled to allow for exclusion after randomization while maintaining the required statistical power. All calculations allowed for a 20% dropout rate.

Data analyses were conducted according to the modified intention-to-treat (mITT) principle, excluding patients with distant metastasis discovered during surgery and those who did not undergo surgery or underwent local resection. The per-protocol analysis included patients who actually underwent the assigned surgery. Categorical variables were presented as numbers (percentages) and compared using the χ^2^ test or Fisher exact test. Continuous variables were presented as median (IQR or range) and analyzed using the *t* test or Mann-Whitney *U* test, as appropriate. All statistical tests were 2-sided, and statistical significance was set at *P* < .05. SAS software (version 9.4; SAS Institute Inc) was used for all statistical analyses.

## Results

### Patients

Between November 2013 and June 2018, 1070 patients were randomized to undergo laparoscopic (n = 712) or open (n = 358) surgery. Thirty-one patients (laparoscopic, 27; open, 4) were excluded from the mITT analysis because of refusal to undergo surgery, distant metastasis identified after randomization, local resection, nonrectal cancer, or upper rectal cancer. Thus, 1039 patients (laparoscopic, 685; open, 354) were included in the mITT analysis (median [range] age, 57 [20-75] years; 620 men [59.7%]; 659 [63.4%] with clinical TNM stage II/III disease). We excluded an additional 70 patients from the per-protocol population; of these, 53 underwent surgery opposite to what they were randomly assigned (50 were randomized to open surgery, and 3 to laparoscopic surgery), and 17 required switching to open surgery for various reasons. The per-protocol population included 969 patients (665 in laparoscopic and 304 in open surgery) (Figure). There were no significant between-group differences in age, sex, BMI, American Society of Anesthesiologists classification, comorbidities, and tumor distance from the dentate line. More than 95% of patients with clinical stage II/III disease in either group received preoperative chemoradiotherapy. Patient baseline characteristics are summarized in Table 1.

**Figure.**
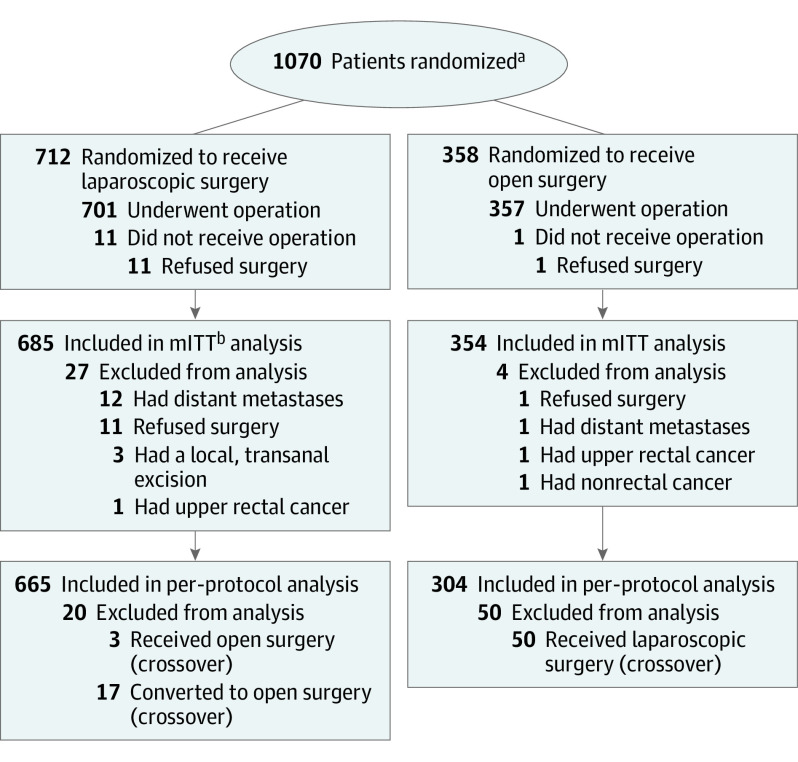
Flowchart of Patient Enrollment and Randomization ^a^Data are not available for the number of patients screened for eligibility. ^b^Abbreviation: mITT, modified intention to treat.

**Table 1.** Patient Baseline Demographic and Clinical Characteristics

Characteristic	No. (%)	
	Laparoscopic surgery (n = 685)	Open surgery (n = 354)
Age, median (IQR), y	58.0 (50.0-64.0)	57.0 (50.0-63.0)
Sex		
Male	409 (59.7)	211 (59.6)
Female	276 (40.3)	143 (40.4)
BMI		
Median (IQR)	22.9 (20.8-25.0)	23.0 (20.9-25.1)
Underweight or normal (<25.0)	517 (75.5)	261 (73.7)
Overweight (25.0-30.0)	154 (22.5)	88 (24.9)
Obese (>30.0)	14 (2.0)	5 (1.4)
ECOG performance status^a^		
0	520 (76.0)	265 (74.9)
1	160 (23.4)	86 (24.3)
2	4 (0.6)	3 (0.8)
ASA score		
I	484 (70.8)	258 (72.9)
II	191 (27.9)	92 (26.0)
III	9 (1.3)	4 (1.1)
Comorbidity		
Yes	184 (26.9)	97 (27.4)
No	501 (73.1)	257 (72.6)
Tumor distance from dentate line, median (IQR), mm^b^	30 (20-40)	30 (20-40)
Clinical TNM stage^c^		
I	252 (36.8)	128 (36.2)
II/III	433 (63.2)	226 (63.8)
Preoperative therapy^c^		
Chemoradiotherapy	415 (95.8)	219 (96.9)
Radiotherapy alone	0	1 (0.4)
Chemotherapy alone	3 (0.7)	0

Abbreviations: ASA, American Society of Anesthesiologists; BMI, body mass index, calculated as weight in kilograms divided by height in meters squared; ECOG, Eastern Cooperative Oncology Group.

^a^ Data were obtained from 1038 patients.

^b^ Data were obtained from 1034 patients. Tumors that invaded the dentate line in 5 patients were excluded.

^c^ Only patients with clinical stage II/III disease were included.

### Pathologic Outcomes

The pathologic outcomes are presented in Table 2. Quality of TME was assessed in 921 patients. The rate of complete TME was 85.3% in the laparoscopic group vs 85.8% in the open group (difference, −0.5%; 95% CI, −5.1% to 4.5%; *P* = .78). In subgroup analysis that only included patients with clinical stage I disease, the rate of complete TME was 86.8% in the laparoscopic group vs 86.7% in the open group (difference, −0%; 95% CI, −7.5% to 9.1%; *P* = .53). In subgroup analysis that only included patients with clinical stage II/III disease, the rate of complete TME was 84.5% in the laparoscopic group vs 85.4% in the open group (difference, −0.9%; 95% CI, −6.5% to 5.4%; *P* = .32) (eTable 2 in Supplement 1).

**Table 2.** Pathologic Outcomes in the Overall Analysis

Characteristic	No. (%)		Difference (95% CI)	*P* value
	Laparoscopic surgery (n = 685)	Open surgery (n = 354)		
TME quality^a^				
Complete	521 (85.3)	266 (85.8)	−0.5 (−5.1 to 4.5)	.78
Nearly complete	74 (12.1)	34 (11.0)	1.1 (−3.5 to 5.3)	
Incomplete	16 (2.6)	10 (3.2)	−0.6 (−3.4 to 1.6)	
Length of PRM, median (IQR), mm	124 (95 to 150)	130 (100 to 155)	−6.0 (−15.4 to 3.4)	.02
Length of DRM, median (IQR), mm	20 (13 to 30)	20 (13 to 30)	0.0 (−1.7 to 1.7)	.92
Negative CRMs	673 (98.2)	353 (99.7)	−1.5 (−2.8 to 0.0)	.09
Negative DRM	681 (99.4)	354 (100.0)	0.6 (−1.5 to 0.5)	.36
No. of retrieved lymph nodes, median (IQR)	13 (9 to 17)	12 (9 to 17)	1.0 (0.1 to 1.9)	.39
Pathologic TNM stage				
0/pCR	93 (13.6)	41 (11.6)	2.0 (−2.5 to 6.0)	.96
I	252 (36.8)	132 (37.3)	−0.5 (−6.7 to 5.6)	
IIA	141 (20.6)	82 (23.2)	−2.6 (−8.1 to 2.6)	
IIB	23 (3.4)	11 (3.1)	0.2 (−2.4 to 2.4)	
IIC	1 (0.1)	1 (0.3)	−0.1 (−1.4 to 0.6)	
IIIA	54 (7.9)	29 (8.2)	−0.3 (−4.1 to 3.0)	
IIIB	104 (15.2)	49 (13.8)	1.3 (−3.4 to 5.7)	
IIIC	17 (2.5)	9 (2.5)	−0.1 (−2.5 to 1.8)	

Abbreviations: CRM, circumferential resection margin; DRM, distal resection margin; pCR, pathological complete response; PRM, proximal resection margin; TME, total mesorectal excision.

^a^ Data were obtained from 921 patients.

There were no significant between-group differences in the rates of negative CRM (98.2% vs 99.7%; difference, −1.5%; 95% CI, −2.8% to 0.0%; *P* = .09) and negative DRM (99.4% vs 100%; difference, −0.6%; 95% CI, −1.5% to 0.5%; *P* = .36), as well as the number of retrieved lymph nodes (median of 13.0 vs 12.0; difference, 1.0; 95% CI, 0.1-1.9; *P* = .39).

### Surgical Outcomes

The surgical details are listed in Table 3. Seventeen patients (2.5%) in the laparoscopic group required conversion to open surgery because of abdominal adhesion (n = 7), adhesion to adjacent organs (n = 3), tumor fixity (n = 3), obesity (n = 1), hypercapnia (n = 1), and other reasons (n = 2). Laparoscopic surgery had a longer operating time (195.0 vs 180.0 minutes; difference, 15.0; 95% CI, 6.2-23.8; *P* < .001) and lower estimated intraoperative blood loss (50.0 vs 100.0 mL; difference, 50.0; 95% CI, −50.0 to 50.0; *P* < .001). Intraoperative complications occurred in 12 patients (1.8%) in the laparoscopic group and 7 patients (2.0%) in the open group for different reasons, with no significant between-group difference (difference, −0.2%; 95% CI, −2.4% to 1.4%; *P* = .80).

**Table 3.** Surgical Details

Characteristic	No. (%)		Difference (95% CI)	*P* value
	Laparoscopic surgery (n = 685)	Open surgery (n = 354)		
Conversion to open surgery^a^	17 (2.5)	NA	NA	NA
Operative time, median (IQR), min	195.0 (155.0 to 240.0)	180.0 (140.0 to 220.0)	15.0 (6.2 to 23.8)	<.001
Estimated blood loss, median (IQR), mL	50.0 (30.0 to 100.0)	100.0 (50.0 to 100.0)	−50.0 (−50.0 to 50.0)	<.001
Intraoperative complications^b^	12 (1.8)	7 (2.0)	−0.2 (−2.4 to 1.4)	.80
Type of surgery				
Low anterior resection	431 (62.9)	200 (56.5)	6.4 (0.2 to 12.7)	.09
Intersphincteric resection	57 (8.3)	27 (7.6)	0.7 (−3.0 to 4.0)	
Abdominoperineal resection	194 (28.3)	123 (34.7)	−6.4 (−12.5 to −0.5)	
Other^c^	3 (0.4)	4 (1.1)	−0.7 (−2.5 to 0.4)	
Sphincter preservation^d^	491 (71.7)	230 (65.0)	6.7 (0.8 to 12.8)	.03
Diverting ostomy^e^				
Yes	387 (78.8)	168 (73.0)	5.8 (−0.8 to 12.7)	.09
No	104 (21.2)	62 (27.0)	−5.8 (−12.7 to 0.8)	
Type of diverting ostomy				
Ileostomy	370 (95.6)	162 (96.4)	−0.8 (−4.0 to 3.5)	.66
Colostomy	17 (4.4)	6 (3.6)	0.8 (−3.5 to 4.0)	

Abbreviation: NA, not applicable.

^a^ Data were obtained from 682 patients, and 3 patients underwent open surgery.

^b^ Intraoperative complications occurred in 12 patients in the laparoscopic group (bleeding [n = 9], hypercapnia [n = 1], anastomotic bleeding [n = 1], and rectum perforation [n = 1]) and 7 patients in the open group (bleeding [n = 4], presacral hemorrhage [n = 1], prostate injury [n = 1], and vaginal injury [n = 1]).

^c^ In the laparoscopic group, 2 patients underwent the Hartmann procedure, and 1 underwent posterior pelvic resection. In the open group, 2 underwent transanal total mesorectal excision, 1 underwent the Hartmann procedure, and 1 underwent total proctocolectomy.

^d^ Only patients who underwent sphincter-preserving surgery were included. In the laparoscopic group, 431 patients underwent low anterior resection, 57 underwent intersphincteric resection, 2 underwent the Hartmann procedure, and 1 underwent posterior pelvic resection. In the open group, 200 patients underwent low anterior resection, 27 underwent intersphincteric resection, 2 underwent transanal total mesorectal excision, and 1 underwent the Hartmann procedure.

^e^ Only patients who underwent sphincter-preserving surgery were included.

The rate of sphincter preservation was 71.7% in the laparoscopic group vs 65.0% in the open group (difference, 6.7%; 95% CI, 0.8% to 12.8%; *P* = .03). In the analysis that only included patients with sphincter preservation, the rate of diverting ostomy (mostly ileostomy) was 78.8% in the laparoscopic group vs 73.0% in the open group (difference, 5.8%; 95% CI, −0.8% to 12.7%; *P* = .09).

### Recovery and Postoperative Complications

The postoperative recovery details are shown in Table 4. Compared with open surgery, laparoscopic surgery showed more favorable postoperative outcomes, including less time to first flatus (40.4 vs 44.8 hours; difference, −4.4; 95% CI, −8.6 to −0.2; *P* = .006), less time to first defecation (61.2 vs 66.3 hours; difference, −5.0; 95% CI, −11.5 to 1.5; *P* = .03), shorter duration of analgesic use (45.0 vs 48.0 hours; difference, −3.0; 95% CI, −6.2 to 0.2; *P* = .001), and shorter duration of hospitalization (8.0 vs 9.0 days; difference, −1.0; 95% CI, −1.7 to −0.3; *P* = .008).

**Table 4.** Postoperative Recovery and Complications

Characteristics	No. (%)		Difference (95% CI)	*P* value
	Laparoscopic surgery (n = 685)	Open surgery (n = 354)		
Postoperative recovery, median (IQR)				
Time to first flatus, h	40.4 (18.6 to 64.2)	44.8 (22.7 to 65.7)	−4.4 (−8.6 to −0.2)	.006
Time to first defecation, h	61.2 (29.2 to 94.5)	66.3 (36.5 to 108.5)	−5.0 (−11.5 to 1.5)	.03
Time to liquid diet, h	46.0 (22.5 to 87.0)	58.1 (23.3 to 87.4)	−11.5 (−19.1 to −3.9)	.39
Time to normal diet, h	116.0 (70.2 to 164.5)	131.2 (70.1 to 178.9)	−15.1 (−27.5 to −2.7)	.43
Duration of analgesic use, h	45.0 (28.8 to 65.0)	48.0 (39.5 to 68.2)	−3.0 (−6.2 to 0.2)	.001
30-d Postoperative complications	89 (13.0)	61 (17.2)	−4.2 (−9.1 to 0.3)	.07
Type of postoperative complications^a^				
Presacral hemorrhage	1 (0.1)	1 (0.3)	−0.1 (−1.4 to 0.6)	>.99
Active intraabdominal bleeding	3 (0.4)	3 (0.8)	−0.4 (−2.0 to 0.6)	.69
Anastomotic bleeding^b^	3 (0.6)	2 (0.9)	−0.3 (−2.6 to 1.1)	.57
Anastomotic leakage^c^	12 (2.5)	14 (6.1)	−3.7 (−7.7 to −0.6)	.01
Chylous leakage	4 (0.6)	0	0.6 (−0.5 to 1.5)	.36
Ileus	15 (2.2)	10 (2.8)	−0.6 (−3.1 to 1.3)	.53
Incision complications	18 (2.6)	18 (5.1)	−2.5 (−5.4 to −0.1)	.04
Stoma-related complications	2 (0.3)	0	0.3 (−0.8 to 1.1)	.55
Urinary disorder	13 (1.9)	2 (0.6)	1.3 (−0.3 to 2.7)	.09
Urinary tract infection	7 (1.0)	2 (0.6)	0.5 (−1.1 to 1.6)	.69
Cardiovascular event	2 (0.3)	4 (1.1)	−0.8 (−2.6 to 0.2)	.21
Pneumonia	6 (0.9)	6 (1.7)	−0.8 (−2.8 to 0.6)	.39
Others	18 (2.6)	6 (1.7)	0.9 (−1.7 to 2.7)	.34
Clavien-Dindo classification				
I-II	84 (12.3)	54 (15.3)	−3.0 (−7.7 to 1.3)	.07
IIIa-IVa	5 (0.7)	7 (2.0)	−1.2 (−3.3 to 0.2)	
30-d Mortality	0	0	0.0 (−0.6 to 1.1)	NA
Duration of hospitalization, median (IQR), d	8.0 (7.0 to 11.0)	9.0 (7.0 to 12.0)	−1.0 (−1.7 to −0.3)	.008

Abbreviation: NA, not applicable.

^a^ The number of individual complications exceeded the total number of complications because patients may have had 2 or more complications.

^b^ Patients who underwent sphincter-preserving surgery and 1 who underwent the Hartmann procedure were excluded.

^c^ Patients who underwent sphincter-preserving surgery and 2 patients who underwent the Hartmann procedure were excluded.

There was no significant difference in postoperative complications rate between the 2 groups (13.0% vs 17.2%; difference, −4.2%; 95% CI, −9.1% to 0.3%; *P* = .07). The rate of severe postoperative complications was 0.7% and 2.0% in the laparoscopic and open groups, respectively (difference, −1.2%; 95% CI, −3.3% to 0.2%; *P* = .07). The laparoscopic group had lower rates of anastomotic leakage (2.5% vs 6.1%; difference, −3.7%; 95% CI, −7.7% to −0.6%; *P* = .01) and incisional complication (2.6% vs 5.1%; difference, −2.5%; 95% CI, −5.4% to −0.1%; *P* = .04). No patient died within 30 days in either group.

### Sensitivity Analysis

Results from the per-protocol analysis were largely consistent with the mITT analysis (eTables 3-6 in Supplement 1).

## Discussion

Laparoscopic surgery provided comparable pathologic outcomes to those of open surgery in terms of complete TME, negative CRM and DRM, and the number of retrieved lymph nodes. Moreover, rates of conversion to open surgery and occurrences of postoperative complications were low, with no 30-day mortality. As reported previously,^6,7,8,9^ other confirmed advantages of laparoscopic surgery over open surgery were observed, including lower blood loss, faster recovery of bowel function, and shorter durations of analgesic use and hospitalization.

Compared with incomplete TME, complete TME is associated with lower circumferential margin involvement and local recurrence rates.^2,3,4,16^ In this trial, the complete TME rates were comparable between the 2 groups in both the overall analysis and subgroup analyses that separated stage I and stage II/III disease. The overall complete TME rate in the laparoscopic group (85.3%) was similar to that in the COLOR II^7^ (88%) and ALaCaRT^9^ (87%) trials, but higher than that in the MRC CLASICC^5^ (77%), COREAN^6^ (72.4%), and ACOSOG Z6051^8^ (72.9%) trials. In this trial, the incomplete TME rate in the laparoscopic group (2.6%) was seemingly lower than that reported following a subgroup analysis of low rectal cancer in the COLOR II trial (5.5%).^7^ Taken together, the results suggest that laparoscopic surgery performed by experienced surgeons could facilitate high-quality mesorectal resection in low rectal cancer.

Circumferential margin involvement is a well-recognized predictor of local recurrence after TME surgery.^17,18^ In this trial, the negative CRM rate did not differ significantly between groups. The negative CRM rate after laparoscopic surgery (98.2%) was similar to that in the COREAN trial (97.1%),^6^ and higher than that in the MRC CLASICC (84.0%),^5^ ACOSOG Z6051 (87.9%),^8^ COLOR II (90%),^7^ and ALaCaRT (93.0%)^9^ trials. This was consistent with the high-quality TME obtained in this trial (85.3% complete TME and 12.1% nearly complete TME). Statistical analysis did not show a significant reduction in the rate of negative CRM in the laparoscopic group (98.2% vs 99.7% in the open group; *P* = .09). A similar finding (ie, a statistically nonsignificant trend for a low rate of negative CRM) has been previously reported by the MRC CLASICC and ACOSOG Z6051 trials,^5,8^ but not by the COREAN and ALaCaRT trials.^6,9^ In the COLOR II trial,^7^ a higher rate of negative CRM was observed in the laparoscopic group. The results of the negative CRM rate in this trial, combined with those of the COLOR II trial, supported the effectiveness of laparoscopic surgery in achieving negative pathological CRMs in low rectal cancer.

In this trial, there was no significant difference in the DRM length and negative DRM rate between the laparoscopic (99.4%) and open (100%) groups. The rate of negative DRM in this trial was similar to that reported by the ACOSOG Z6051 trial^8^ (98.3% for laparoscopic surgery, 98.2% for open surgery) and ALaCaRT trial^9^ (99% for both surgery types). In previous studies, a negative DRM rate similar to that reported in this trial translated into comparable local recurrence, regardless of the surgical approach.^19,20,21^

The open surgery conversion rate in this trial (2.5%) was comparable with that in the COREAN trial (1.2%)^6^ but notably lower than reported by other trials (9%-34%).^5,7,8,9^ The low conversion rate in this trial may be attributed to the strict criteria for surgeon qualifications and the advancement of laparoscopic instruments and surgical skills during the study period. A similar phenomenon occurred in the MRC CLASICC trial,^3^ wherein the conversion rate decreased from 38% in the first year to 16% in the sixth year.

Consistent with findings of previous trials,^5,6,7,8,9^ laparoscopic surgery was associated with a longer operating time and lower volumes of intraoperative blood loss. Owing to patient characteristics (low rectal cancer) in this trial, the rate of abdominoperineal resection (laparoscopic, 28.3%; open, 34.7%) was higher than in previous trials.^6,7,8,9^ Nonetheless, a higher rate of sphincter preservation was observed in the laparoscopic group than in the open group. Such a finding was consistent with the results in previous studies.^11,22,23^ The underlying reasons for such a difference are unknown, but it could be reasonably attributed arbitrarily to the advantages of laparoscopic surgery in magnifying and visualizing deep pelvic structures, and thus more confidence in successfully completing adequate resection while preserving the sphincters.

The postoperative complications rate in the laparoscopic group in this trial (13.0%) was lower than that reported in previous trials (23.5%-57.1%).^3,5,7,8^ The rate of overall postoperative complications did not differ between the 2 groups. However, the rates of anastomotic leakage and incisional complications were lower in the laparoscopic group, adding support to the safety and benefits of laparoscopic surgery. Diverting ostomy does not reduce the rate of anastomotic leakage in patients at low risk of anastomotic leakage.^24,25,26,27^ In high-risk patients, however, diverting ostomy is one of the most effective measures that could be taken to prevent or ameliorate anastomotic leakage.^28,29,30,31^ Accordingly, the lower rate of anastomotic leakage in the laparoscopic group in this trial could be partly attributed to the higher rate of diverting ostomy.

### Limitations

This study had several limitations. First, the experience required from participating surgeons appears to be more stringent than that in earlier trials. Surgical skills affect the quality of rectal cancer surgery^32,33^; therefore, it remains unknown whether comparable results will be achieved with less-experienced surgeons. Second, the median BMI of enrolled patients was significantly lower than that of typical Western populations. The generalizability of the results for overweight or obese patients requires further investigation. Third, 50 patients (14.1%) randomized to the open group did not undergo the allocated surgery; instead, they opted to undergo laparoscopic surgery. The inclusion of these participants in the data analysis conformed with the mITT principle and may have biased the results; however, after excluding these crossover patients, the per-protocol population analysis revealed similar short-term results.

## Conclusions

In the LASRE randomized clinical trial, findings demonstrated that laparoscopic surgery for low rectal cancer, when performed by experienced surgeons, could yield pathologic outcomes comparable to those of open surgery in terms of complete mesorectal excision and negative resection margins, with a higher sphincter preservation rate and favorable postoperative recovery. Long-term oncological outcomes are currently being evaluated and will be addressed in future studies.

## Notes

Supplement 1. **eAppendix.** Trial protocol modification in ClinicalTrials.gov **eTable 1.** Eligibility Criteria for the Enrollment of the Patients **eTable 2.** Pathologic Outcomes in the Subgroup Analysis Based on Disease Stage in the mITT Population **eTable 3.** Patient Baseline Demographic and Clinical Characteristics in the Per-Protocol Population **eTable 4.** Pathologic Outcomes in the Per-Protocol Population **eTable 5.** Surgical Details in the Per-Protocol Population **eTable 6.** Postoperative Recovery and Complications in the Per-Protocol Population

Supplement 2. Trial Protocol and Statistical Analysis Plan

Supplement 3. LASRE trial investigators

Supplement 4. Data Sharing Statement
