# Supplementary material for: Short-term Outcomes of Laparoscopy-Assisted vs Open Surgery for Patients With Low Rectal Cancer: The LASRE Randomized Clinical Trial
Source: JAMA Oncol. 2022 Sep 15;8(11):1607–15. doi: 10.1001/jamaoncol.2022.4079 (PMC9478880; doi:10.1001/jamaoncol.2022.4079)
Supplement: Supplement 1. — eAppendix. Trial protocol modification in ClinicalTrials.gov eTable 1. Eligibility Criteria for the Enrollment of the Patients eTable 2. Pathologic Outcomes in the Subgroup Analysis Based on Disease Stage in the mITT Population eTable 3. Patient Baseline Demographic and Clinical Characteristics in the Per-Protocol Population eTable 4. Pathologic Outcomes in the Per-Protocol Population eTable 5. Surgical Details in the Per-Protocol Population eTable 6. Postoperative Recovery and Complications in the Per-Protocol Population [file jamaoncol-e224079-s001.pdf]

Supplemental Online Content

Jiang WZ, Xu JM, Xing JD, et al; for the LASRE trial investigators. Short-term outcomes of laparoscopy-assisted vs open surgery for patients with low rectal cancer: the LASRE randomized clinical trial. *JAMA Oncol.* Published online September 15, 2022. doi:10.1001/jamaoncol.2022.4079

eTable 1. Eligibility Criteria for the Enrollment of the Patients

eTable 2. Pathologic Outcomes in the Subgroup Analysis Based on Disease Stage in the mITT Population

eTable 3. Patient Baseline Demographic and Clinical Characteristics in the Per-Protocol Population

eTable 4. Pathologic Outcomes in the Per-Protocol Population

eTable 5. Surgical Details in the Per-Protocol Population

eTable 6. Postoperative Recovery and Complications in the Per-Protocol Population

This supplemental material has been provided by the authors to give readers additional information about their work.

© 2022 Jiang WZ et al. *JAMA Oncology.*

eAppendix. Trial protocol modification in ClinicalTrials.gov

I cross-examined all modifications in the trial registry and our trial records, and wish to make the following clarifications: 1) Tumor size for eligibility criteria has never been modified. We did not specify the tumor size requirement in the first version (July 11, 2013) and added the specification (<6 cm diameter) in the version (November 5, 2013). DSMB was not involved in this modification. 2) Primary endpoint was a 5-year OS rate in the first version (July 11, 2013) and changed to a 3-year DFS rate in the version (October 7, 2013). This change was made based on the recommendation of the DSMB and prior to the enrollment of the first patient. 3) Definitions of secondary endpoints were added to the registry upon a subsequent amendment. Indeed, several secondary outcomes in the first version (i.e., negative CRM/DRM, length of DRM, and number of retrieved lymph nodes) were placed under an umbrella concept of "pathologic outcomes" in a subsequent version (September 26, 2021). DSMB was not involved in this modification.

Date	Section	Description
July 11, 2013		LASRE registration
July 14, 2013	Inclusion Criteria	Replacing "T3" with "T3-4"
October 7, 2013	Title	Adding "open-label"
	Sponsor/Collaborators	<u>Adding study sites:</u><br>Sixth Affiliated Hospital Sun Yat-sen University, Peking Union Medical College Hospital, Beijing Cancer Hospital, Liaoning Tumor Hospital & Institute, Union Hospital Huazhong University of Science and Technology, West China Hospital, The Second Affiliated Hospital of Fujian Medical University, The First Affiliated Hospital of Xiamen University, Zhangzhou Affiliated Hospital of Fujian Medical University, Longyan Affiliated Hospital of Fujian Medical University
	Primary Outcome Measures	Replacing "overall survival [Time Frame: 5 years]" with "disease-free survival [Time Frame: 3 years]"
	Secondary Outcome Measures	Replacing "disease-free survival [Time Frame: 5 years] with "overall survival [Time Frame: 3 and 5 years]"
	Inclusion Criteria	Changing "7 cm from the anal verge" to "within 5 cm from the dentate line"
October 9, 2013	Sponsor/Collaborators	<u>Adding study site:</u><br>Shengjing Hospital
November 5, 2013	Sponsor/Collaborators	<u>Adding study sites:</u><br>Second Affiliated Hospital, School of Medicine, Zhejiang University
	Inclusion Criteria	Replacing "T3-4, N0 or Tany, N1-2" with "T3-4a, N0 or T1-4a, N1-2"<br>Adding: tumor size <6cm
August 16, 2015	Sponsor/Collaborators	<u>Adding study sites:</u><br>Wuhan Union Hospital, Fudan University Shanghai

© 2022 Jiang WZ et al. *JAMA Oncology.*

		Zhongshan Hospital, Sun Yat-sen University Cancer Center, Hubei Cancer Hospital, Zhejiang Cancer Hospital
September 26, 2021	Primary Outcome Measures	Adding definition for disease-free survival [Time Frame: 3 years]: Disease-free survival is defined as the time from date of surgery to the date of rectal cancer recurrence or metastasis or cancer-related death (locoregional or distant recurrence).
	Secondary Outcome Measures	Placing individual pathologic outcomes, including distal resection margin [Time Frame: 1 week post operatively], circumferential margin [Time Frame: 1 week post operatively], and proximal resection margin [Time Frame: 1 week post operatively], under an umbrella scheme of Pathologic outcomes [Time Frame: 1 week post operatively] Adding definitions;<br>Pathologic outcomes are defined as TME quality, negative CRM and negative DRM, length of proximal resection margin (PRM), length of DRM, and the number of retrieved lymph nodes. The TME quality was graded based on the criteria proposed by Nagtegaal et al. as complete, nearly complete, or incomplete. Positive resection margin, including circumferential resection margin (CRM) and distal resection margin (DRM), was defined as the presence of cancer cells within 1 mm from the cut edge.
		Adding definition for 30-day postoperative complications [Time Frame: 1 month within operatively]: Thirty-day postoperative complications included any complications occurring within 30 days after surgery. Postoperative complications were graded according to the Clavien-Dindo classification. Severe complications were defined as Clavien-Dindo III-V.
		Adding definition for 30-day postoperative mortality [Time Frame: 30 days post operatively]: Thirty-day operative mortality is defined as deaths occurring from any cause during the first 30 postoperative days.
		Adding definition for locoregional recurrence rate [Time Frame: 3 and 5 years post operatively]: Locoregional recurrence was defined as the presence of any anastomotic, pelvic or perineal tumour documented by clinical and/or pathological examination.
		Placing distal resection margin [Time Frame: 1 week post operatively] under Pathologic outcomes
		Placing circumferential margin [Time Frame: 1 week post operatively] under Pathologic outcomes

© 2022 Jiang WZ et al. *JAMA Oncology*.

		Placing proximal resection margin [Time Frame: 1 week post operatively] under Pathologic outcomes

© 2022 Jiang WZ et al. *JAMA Oncology*.

eTable 1. Eligibility Criteria for the Enrollment of the Patients

**Inclusion criteria**
Aged 18-75 years
Pathological diagnosis of rectal adenocarcinoma (including highly and moderately differentiated tubular adenocarcinoma, papillary adenocarcinoma, poorly differentiated tubular adenocarcinoma, mucinous adenocarcinoma, and signet-ring cell carcinoma)
The lower margin of the tumor is < 5 cm from the dentate line at the time of initial diagnosis by rigid proctoscopy
cT1-3N0-2M0 or cT4aN0-2M0 adenocarcinoma after neoadjuvant chemoradiotherapy. Patients with pelvic lateral lymph nodes are ineligible
Primary tumor < 6 cm in size
No other concurrent primary cancers
Adequate function of main organs, allowing surgical treatment
Patients and their family members can understand the study plan, are willing to participate, and agree to give written informed consent
**Exclusion criteria**
Aged < 18 or > 75 years;
Concurrent or previous malignancies within five years
Need for emergency surgery due to intestinal obstruction, intestinal perforation, intestinal hemorrhage, etc.
Previous history of colorectal surgery that might affect the reconstruction of the digestive tract
Need to remove other organs in addition to the rectum
ASA classification IV or V
Current pregnancy or lactation:<br>● Women of childbearing age with a positive pregnancy test at baseline or who have not taken a pregnancy test; postmenopausal women must be at least 12 months postmenopausal<br>● Sexually active men and women (of reproductive age) who are unwilling to take contraceptive measures during the study period
Severe mental illness
Inability to tolerate surgery due to severe emphysema, interstitial pneumonia, ischemic heart disease, etc.
Continuous systemic steroid therapy within the last month
Contraindications to laparoscopic surgery
Patients and their family members cannot understand the conditions and objectives of this study

© 2022 Jiang WZ et al. *JAMA Oncology.*

eTable 2. Pathologic Outcomes in the Subgroup Analysis Based on Disease Stage in the mITT Population

Characteristics	Laparoscopic Surgery (n = 685)	Open Surgery (n = 354)	Difference	95% CI	*P* Value
TME quality No. (%)					
Stage I disease, No. (%) [a]					
Complete	177 (86.8)	85 (86.7)	0.0	-7.5 to 9.1	.53
Nearly complete	21 (10.3)	12 (12.2)	-2.0	-10.6 to 5.1	
Incomplete	6 (2.9)	1 (1.0)	1.9	-2.9 to 5.4	
Stage II/III disease, No. (%) [b]					
Complete	344 (84.5)	181 (85.4)	-0.9	-6.5 to 5.4	.32
Nearly complete	53 (13.0)	22 (10.4)	2.6	-3.0 to 7.6	
Incomplete	10 (2.5)	9 (4.2)	-1.8	-5.6 to 1.0	
Length of PRM, median, (IQR) mm					
Stage I disease	115 (86–150)	118 (95–150)	-3.0	-20.2 to 14.2	.09
Stage II/III disease	128 (100–154)	135 (100–165)	-7.0	-17.8 to 3.8	.09
Length of DRM, median, (IQR) mm					
Stage I disease	20 (11–30)	20 (10–27)	0.0	-2.5 to 2.5	.19
Stage II/III disease	21 (15–35)	25 (17–35)	-3.0	-6.2 to 0.2	.47
Negative CRMs, No. (%)					
Stage I disease	248 (98.4)	128 (100.0)	-1.6	-4.0 to 1.5	.37
Stage II/III disease	425 (98.2)	225 (99.6)	-1.4	--3.2 to 0.8	.26
Negative DRMs, No. (%)					
Stage I disease	252 (100.0)	128 (100.0)	0.0	-1.5 to 2.9	NA
Stage II/III disease	429 (99.1)	226 (100.0)	-0.9	-2.4 to 0.8	.36
Retrieved lymph nodes, median, (IQR)					
Stage I disease	15 (12–19)	14.5 (11–19)	1.0	-0.8 to 2.8	.68
Stage II/III disease	12 (8–16)	12 (6–15)	0.0	-1.0 to 1.0	.60
Pathologic T stage, No. (%)					
T0/ Tis	99 (14.5)	41 (11.6)	2.8	-1.6 to 6.9	.87
T1	66 (9.6)	34 (9.6)	0.0	-4.0 to 3.6	
T2	243 (35.5)	131 (37.0)	-1.6	-7.8 to 4.5	
T3	238 (34.7)	129 (36.4)	-1.7	-7.9 to 4.3	
T4a	36 (5.3)	17 (4.8)	0.4	-2.7 to 3.1	
T4b	3 (0.4)	2 (0.6)	-0.1	-1.6 to 0.8	

© 2022 Jiang WZ et al. *JAMA Oncology.*

eTable 2. Pathologic Outcomes in the Subgroup Analysis Based on Disease Stage in the mITT Population（continued）

Pathologic N stage, No. (%)					
N0	511 (74.6)	266 (75.1)	-0.7	-6.1 to 5.0	.79
N1a	70 (10.2)	32 (9.0)	1.2	-2.8 to 4.8	
N1b	58 (8.5)	31 (8.8)	-0.3	-4.2 to 3.1	
N1c	7 (1.0)	3 (0.8)	0.2	-1.5 to 1.4	
N2a	28 (4.1)	12 (3.4)	0.7	-2.0 to 3.0	
N2b	12 (1.7)	10 (2.8)	-1.2	-3.6 to 0.6	

Note. Data are presented as number (%) or median (IQR).

Abbreviations: CI, confidence interval; TME, total mesorectal excision; PRM, proximal resection margin; IQR, interquartile range; DRM, distal resection margin; CRM, circumferential resection margin; NA, not applicable.

[a] Data were obtained from 302 patients.

[b] Data were obtained from 619 patients.

© 2022 Jiang WZ et al. *JAMA Oncology.*

eTable 3. Patient Baseline Demographic and Clinical Characteristics in the Per-Protocol Population

Characteristics	Laparoscopic Surgery (n = 665)	Open Surgery (n = 304)
Age, median (IQR), years	57.0 (50.0–64.0)	57.0 (50.0–63.0)
Sex, No. (%)		
Male	394 (59.2)	184(60.5)
Female	271(40.8)	120(39.5)
BMI, median, (IQR), kg/m^2	22.9 (20.8–25.0)	23.1 (20.9–25.3)
Underweight and normal (< 25.0), No. (%)	501(75.3)	217(71.4)
Overweight (25.0 - 30.0), No. (%)	151(22.7)	83(27.3)
Obese (> 30.0), No. (%)	13(2.0)	4(1.3)
ECOG performance status, No. (%)[a]		
0	507(76.4)	228(75.0)
1	154(23.2)	75(24.7)
2	3(0.5)	1(0.3)
ASA score, No. (%)		
I	471(70.9)	221(72.7)
II	184(27.7)	80(26.3)
III	9(1.4)	3(1.0)
Comorbidity, No. (%)		
Yes	181(27.2)	88(28.9)
No	484(72.8)	216(71.1)
Tumor distance from dentate line, median, (IQR), mm [b]	30.0 (20.0-40.0)	30.0 (20.0-40.0)
Clinical TNM stage, No. (%)		
I	246(37.0)	96(31.6)
II/III	419(63.0)	208(68.4)
Preoperative therapy, No. (%)[c]		
Chemoradiotherapy	403(96.2)	202(97.1)
Radiotherapy alone	0(0)	1(0.5)
Chemotherapy alone	3(0.7)	0(0)

Note. Data are presented as number (%) or median (interquartile range, IQR).

Abbreviations: IQR, interquartile range; BMI, body mass index; ECOG, Eastern Cooperative Oncology Group; ASA, American Society of Anesthesiologists.

[a] Data were obtained from 968 patients.

[b] Data were obtained from 964 patients. Tumors that invaded the dentate line in five patients were excluded.

[c] Only patients with clinical stage II/III disease were included.

© 2022 Jiang WZ et al. *JAMA Oncology.*

eTable 4. Pathologic Outcomes in the Per-Protocol Population

Characteristics	Laparoscopic Surgery (n = 665)	Open Surgery (n = 304)	Difference	95% CI	*P* Value
TME quality No. (%)					
Overall [a]					
Complete	507(85.6)	242(87.4)	-1.7	-6.3 to 3.4	.78
Nearly complete	69(11.7)	28(10.1)	1.5	-3.2 to 5.7	
Incomplete	16(2.7)	7(2.5)	-0.2	-2.6 to 2.3	
Stage I disease, No. (%) [b]					
Complete	172(86.4)	71(88.8)	-2.3	-9.9 to 7.4	.69
Nearly complete	21(10.6)	8(10.0)	-0.6	-8.7 to 7.5	
Incomplete	6(3.0)	1(1.3)	1.8	-4.0 to 5.3	
Stage II/III disease, No. (%) [c]					
Complete	335(85.2)	171(86.8)	-1.6	-7.1 to 4.7	.73
Nearly complete	48(12.2)	20(10.2)	2.1	-3.7 to 7.1	
Incomplete	10(2.5)	6(3.0)	-0.5	-4.1 to 2.1	
Length of PRM, median, (IQR) mm					
Overall	123 (95–150)	135 (100–158)	-12.0	-22.5 to 1.5	<.001
Stage I disease	114 (85–150)	136 (100–150)	-20.0	-37.2 to 2.8	.001
Stage II/III disease	128 (100–153)	135 (100–165)	-7.0	-18.1 to 4.1	.10
Length of DRM, median, (IQR) mm					
Overall	20 (13–30)	20 (15–30)	0.0	-1.7 to 1.7	.76
Stage I disease	20 (11–30)	20 (10–27)	0.0	-2.5 to 2.5	.30
Stage II/III disease	21 (15–35)	25 (17–35)	-3.0	-6.2 to 0.2	.49
Negative CRMs, No. (%)					
Overall	654(98.3)	303(99.7)	-1.3	-2.6 to 0.4	.16
Stage I disease	248 (98.4)	128 (100.0)	-1.6	-4.0 to 2.3	.49
Stage II/III disease	425 (98.2)	225 (99.6)	-1.4	--3.2 to 0.8	.26
Negative DRMs, No. (%)					
Overall	662(99.5)	304(100.0)	0.5	-1.3 to 0.8	.56
Stage I disease	246 (100.0)	96 (100.0)	0.0	-1.5 to 3.8	NA

© 2022 Jiang WZ et al. *JAMA Oncology.*

eTable 4. Pathologic Outcomes in the Per-Protocol Population （continued）

Stage II/III disease	416(99.3)	226 (100.0)	-0.7	-1.8 to 0.9	.55
Retrieved lymph nodes, median, (IQR)					
Overall	13 (9–17)	12 (9–17)	1.0	0.1 to 1.9	.31
Stage I disease	15 (12–19)	15 (11–19)	1.0	-1.3 to 1.3	.80
Stage II/III disease	12 (8–16)	12 (6–15)	0.0	-1.0 to 1.0	.66
Pathologic T stage, No. (%)					
T0/ Tis	98(14.7)	38(12.5)	2.2	-2.6 to 6.6	.76
T1	63(9.5)	29(9.5)	-0.1	-4.4 to 3.7	
T2	240(36.1)	114(37.5)	-1.4	-8.0 to 5.0	
T3	228(34.3)	104(34.2)	0.1	-6.4 to 6.4	
T4a	35(5.3)	17(5.6)	-0.3	-3.8 to 2.5	
T4b	1(0.2)	2(0.7)	-0.5	-2.2 to 0.3	
Pathologic N stage, No. (%)					
N0	499(75.0)	229(75.3)	-0.3	-6.0 to 5.7	.76
N1a	68(10.2)	28(9.2)	1.0	-3.3 to 4.8	
N1b	53(8.0)	30(9.9)	-1.9	-6.2 to 1.8	
N1c	6(0.9)	3(1.0)	-0.1	-2.0 to 1.2	
N2a	28(4.2)	8(2.6)	1.6	-1.2 to 3.8	
N2b	11(1.7)	6(2.0)	-0.3	-2.7 to 1.3	
Pathologic TNM stage, No. (%)					
0/pCR	92(13.8)	38(12.5)	1.3	-3.5 to 5.7	.87
I	247(37.1))	114(37.5)	-0.4	-7.0 to 6.1	
IIA	137(20.6)	66(21.7)	-1.1	-6.9 to 4.2	
IIB	22(3.3)	11(3.6)	-0.3	-3.3 to 2.0	
IIC	0(0)	1 (0.3)	-0.3	-1.8 to 0.3	
IIIA	52(7.8)	26(8.6)	-0.7	-4.8 to 2.8	
IIIB	99(14.9)	42(13.8)	1.1	-3.9 to 5.6	
IIIC	16(2.4)	6(2.0)	0.4	-2.0 to 2.2	

Note. Data are presented as number (%) or median (interquartile range, IQR).

Abbreviations: CI, confidence interval; TME, total mesorectal excision; PRM, proximal resection margin; IQR, interquartile range; CRM, circumferential resection margin; DRM, distal resection margin; NA, not applicable; pCR, pathological complete response.

[a] Data were obtained from 869 patients.

[b] Data were obtained from 279 patients.

[c] Data were obtained from 590 patients.

© 2022 Jiang WZ et al. *JAMA Oncology.*

eTable 5. Surgical Details in the Per-Protocol Population

Characteristics	Laparoscopic Surgery (n = 665)	Open Surgery (n = 304)	Difference	95% CI	*P* Value
Operative time, median, (IQR), min	193.0 (155.0–240.0)	180.0 (140.0–218.0)	13.0	3.8 to 22.2	< .001
Estimated blood loss, median, (IQR), mL	50.0 (30.0–100.0)	100.0 (50.0–100.0)	-50.0	-50.0 to 50.0	< .001
Intraoperative complications No. (%)	1 (0.2)	3 (1.0)	-0.8	-2.7 to 0.1	.18
Type of surgery, No. (%)					
Low anterior resection	419(63.0)	172(56.6)	6.4	0.2 to 13.1	.12
Intersphincteric resection	56(8.4)	25(8.2)	0.2	-3.9 to 3.7	
Abdominoperineal resection	188(28.3)	104(34.2)	-5.9	-12.3 to -0.3	
Others [a]	2(0.3)	3(0.6)	-0.7	-2.6 to 0.3	
Sphincter preservation, No. (%) [b]	477(71.7)	199(65.5)	6.3	0.0 to 12.7	.05
Diverting ostomy, No. (%) [c]					
Yes	376 (78.8)	150(75.4)	3.4	-3.3 to 10.7	.33
No	101 (21.2)	49 (24.6)	-3.4	-10.7, to 3.3	
Type of diverting ostomy					
Ileostomy	360(95.7)	147(98.0)	-2.3	-5.1 to 1.8	.21
Colostomy	16(4.3)	3(2.0)	2.3	-1.8 to 5.1	

Note. Data are presented as number (%) or median (interquartile range, IQR).

Abbreviations: CI, confidence interval; IQR, interquartile range.

[a] In the laparoscopic surgery group, two patients underwent Hartmann's procedure. In the open group, one underwent transanal total mesorectal excision, one underwent Hartmann's procedure, and one underwent total proctocolectomy.

[b] Only patients who underwent sphincter-preserving surgery were included. In the laparoscopic surgery group, 419 patients underwent low anterior resection, 56 underwent intersphincteric resection, and two underwent Hartmann's procedure. In the open surgery group, 172 underwent low anterior resection, 25 underwent intersphincteric resection, one underwent transanal total mesorectal excision, and one underwent Hartmann's procedure.

[c] Only patients who underwent sphincter-preserving surgery were included.

© 2022 Jiang WZ et al. *JAMA Oncology.*

eTable 6. Postoperative Recovery and Complications in the Per-Protocol Population

Characteristics	Laparoscopic Surgery (n = 665)	Open Surgery (n = 304)	Difference	95% CI	*P* Value
Postoperative recovery					
Time to first flatus, median, (IQR), h	40.4 (18.7–63.6)	44.1(20.0–65.4)	-3.5	-7.8 to 0.8	.03
Time to first defecation, median, (IQR), h	61.4 (30.0–94.5)	64.9 (31.1–104.3)	-3.4	-11.7 to 4.9	.34
Time to liquid diet, median, (IQR), h	46.3 (22.5–86.7)	48.2 (20.7–84.5)	-1.8	-9.6 to 6.0	.73
Time to normal diet, median, (IQR), h	116.0 (70.2–164.0)	115.2 (68.2–164.6)	0.7	-14.3 to -15.7	.71
Duration of analgesic use, h	45.1 (28.8–65.2)	49.5 (39.6–68.8)	-4.4	-8.5 to -0.3	<.001
30-day postoperative complications, No. (%)	86 (12.9)	58 (19.1)	-6.1	-11.5 to -1.3	.01
Type of postoperative complications, No. (%)[a]					
Presacral hemorrhage	1 (0.2)	1 (0.3)	-0.2	-1.7 to 0.6	.53
Active intraabdominal bleeding	2 (0.3)	3 (1.0)	-0.7	-2.6 to 0.3	.37
Anastomotic bleeding[b]	3(0.6)	2 (1.0)	-0.4	-3.0 to 1.0	.98
Anastomotic leakage[c]	12 (2.5)	12 (6.0)	-3.5	-7.9 to -0.4	.02
Chylous leakage	4 (0.6)	0 (0)	0.6	-0.7 to 1.5	.42
Ileus	15 (2.3)	9 (3.0)	-0.7	-3.4 to 1.3	.51
Incision complications	16 (2.4)	17(5.6)	-3.2	-6.5 to -0.6	.01
Stoma-related complications	2 (0.3)	0 (0)	0.3	-1.0 to 1.1	.99
Urinary disorder	12 (1.8)	2 (0.7)	1.1	-0.7 to 2.6	.27
Urinary tract infection	6 (0.9)	2 (0.7)	0.2	-1.5 to 1.4	.99
Cardiovascular event	2 (0.3)	3 (1.0)	-0.7	-2.6 to 0.3	.37
Pneumonia	6 (0.9)	6 (2.0)	-1.1	-3.4 to 0.4	.28
Others	18 (2.6)	6 (1.7)	0.9	-1.7 to 2.7	.34
Clavien-Dindo classification, No. (%)					
I-II	81(12.2)	52(17.1)	-4.9	-10.1 to 0.2	.03
IIIa-IVa	5(0.8)	6(2.0)	-1.2	-3.5 to 0.2	
30-day mortality, No. (%)	0 (0)	0 (0)	0.0	-1.2 to 0.6	NA
Duration of hospitalization, median, (IQR), d	8.0 (7.0–11.0)	9.0 (7.0–12.0)	-1.0	-1.7 to -0.3	.007

Note. Data are presented as number (%) or median (interquartile range, IQR).

Abbreviations: CI, confidence interval; NA, not applicable.

[a] The number of individual complications exceeded the total number of complications because one patient may have had more than two complications.

© 2022 Jiang WZ et al. *JAMA Oncology.*

[b] Patients who underwent sphincter-preserving surgery and one who underwent Hartmann's procedure were excluded.

[c] Patients who underwent sphincter-preserving surgery and two patients who underwent Hartmann's procedure were excluded.

© 2022 Jiang WZ et al. *JAMA Oncology*.
