# Supplementary material for: Short-term Outcomes of Laparoscopy-Assisted vs Open Surgery for Patients With Low Rectal Cancer: The LASRE Randomized Clinical Trial
Source: JAMA Oncol. 2022 Sep 15;8(11):1607–15. doi: 10.1001/jamaoncol.2022.4079 (PMC9478880; doi:10.1001/jamaoncol.2022.4079)
Supplement: Supplement 2. — Trial Protocol and Statistical Analysis Plan [file jamaoncol-e224079-s002.pdf]

Laparoscopy-**A**ssisted **S**urgery for Carcinoma of the Low **RE**ctum: A Prospective, Multi-Center, Randomized, Open-Label, Parallel-Group, Non-Inferiority Clinical Trial **(LASRE Trial)**

Study Protocol

Principal investigator（PI）: Prof. Pan Chi, MD，FRCS

Applicant: Fujian Medical University Union Hospital

Version: 3.0

Date: May 18, 2015

Contents

Summary of Study Plan **4**
1 Background **8**
2 Objective **12**
3 Participants **12**
3.1 Inclusion criteria 12
3.2 Exclusion criteria 12
4 Study design **13**
5 Sample size estimation **13**
6 Interventions **13**
6.1 Preoperative chemoradiotherapy regimens 13
6.1.1 Preoperative concurrent chemotherapy protocol 13
6.1.2 Preoperative radiotherapy protocol 14
6.1.3 Preoperative chemotherapy during the interval between preoperative chemoradiotherapy and surgery. 14
6.2 Surgical treatment plan 14
6.2.1 Open surgery 14
6.2.2 Laparoscopic surgery 15
6.3 Adjuvant therapy 15
7 Follow-up **15**
8 Study endpoints **16**
8.1 Primary outcome measure 16
8.2 Secondary outcome measures 16
8.3 Other pre-specified outcome measures 17
8.4 Definition of outcome measures 17
9 The flow of study **19**
9.1 Randomization 19
9.2 Screening of participants 20
9.3 Inclusion of participants 20
9.4 Management of patient participation 20
9.5 Preoperative preparation 21
9.6 Surgical procedures 21
9.6.1 Open surgery 21
9.6.1.1 Low or ultralow anterior resection 22
9.6.1.2 Intersphincteric resection 22
9.6.1.3 Abdominoperineal resection 22
9.6.2 Laparoscopy-assisted surgery 23
9.6. 2.1 Low or ultralow anterior resection 24
9.6. 2.2 Intersphincteric resection 24
9.6. 2.3 Abdominoperineal resection 25
9.7 Intraoperative observation 25
9.8 Adjuvant therapy 25
9.8.1 Adjuvant chemotherapy 26

9.8.2 Adjuvant chemoradiotherapy........26
9.9 Postoperative recovery management........26
9.10 Hospital discharge criteria........26
9.11 Variation of discharge time and the analysis of its causes........27
9.12 Follow-up plan........27
10 Pathological assessment........27
11 Data management........28
11.1 Electronic data management........28
11.2 Data management plan........29
12 Statistical analysis........30
12.1 Analysis datasets........30
12.2 Statistical methods........31
12.3 Statistical software and general requirements........32
13 On-site inspection........32
14 Investigators and research structure........32
14.1 Principal Investigator........32
14.2 Participating centers and co-investigators........32
14.3 Research coordinators …........33
14.4 On-site inspector **........34**
15 Ethical rules of this study........34
15.1 Ethical principles …........34
15.2 Ethical review procedure........34
15.3 Responses to ethical review decisions........34
15.4 Registration of this study........34
15.5 Privacy protection of participants........34
15.6 Modification of the study plan........35
16 Adverse events........35
16.1 Definition of adverse events........35
16.2 Expected adverse events........36
16.3 Grading of adverse events........38
16.4 Recording of adverse events........38
16.5 Reporting of adverse events........38
17 Confirmation of raw data........38
17.1 Raw data........38
17.2 Quality assurance of raw data........39
18 Quality management and control........39
18.1 Clinical research quality management........39
18.2 Quality management of participating centers, and surgeon eligibility........40
18.3 Surgical quality management........40
19 Publication policy........44
20 Changes of study protocol........45
21 References........45
Appendix 1: Trial flow........50
Appendix 2: Assessment schedule and process........51

Appendix 3: ECOG performance status **55**
Appendix 4: The ASA classification of physical status **56**
Appendix 5: Tumor regression grading **57**
Appendix 6: Quality of life (QoL) **58**
6.1 EORTC QLQ-C30 (Version 3.0) 58
6.2 EORTC QLQ-CR29 59
Appendix 7: The International Prostate Symptom Score **62**
Appendix 8: The International Index of Erectile Function Questionnaire (IIEF) **64**
Appendix 9: The Female Sexual Function Index **68**
Appendix 10: The Wexner score **73**
Appendix 11: TNM staging **74**

Summary of Study Plan

**Trial name**	Laparoscopy-assisted surgery for carcinoma of the low rectum: a prospective, multicenter, randomized, open-label, parallel-group, noninferiority clinical trial (LASRE)
**Principal investigator (PI)**	Prof. Pan Chi, MD, FRCS
**Participating hospitals**	● The Sixth Affiliated Hospital, Sun Yat-sen University<br>● Nanfang Hospital, Southern Medical University<br>● Peking Union Medical College Hospital, Chinese Academy of Medical Sciences ＆ Peking Union Medical College<br>● Peking University Cancer Hospital and Institute<br>● The General Hospital of the People's Liberation Army<br>● Fudan University Cancer Center<br>● Zhongshan Hospital, Fudan University<br>● Cancer Hospital of China Medical University, Liaoning Cancer Hospital & Institute<br>● Renji Hospital, Shanghai Jiao Tong University School of Medicine<br>● Union Hospital, Tongji Medical College, Huazhong University of Science and Technology<br>● The Second Affiliated Hospital, School of Medicine, Zhejiang University<br>● West China Hospital, Sichuan University<br>● Sun Yat-sen University Cancer Center<br>● Shengjing Hospital, China Medical University<br>● Cancer Hospital of the University of Chinese Academy of Sciences ＆ Zhejiang Cancer Hospital<br>● Hubei Provincial Cancer Hospital<br>● Fujian Provincial Cancer Hospital<br>● The Second Affiliated Hospital, Fujian Medical University<br>● The First Affiliated Hospital, Xiamen University<br>● Zhangzhou Affiliated Hospital, Fujian Medical University<br>● Longyan Affiliated Hospital, Fujian Medical University
**Study objective**	To evaluate the perioperative safety and long-term efficacy of laparoscopy-assisted surgery in the treatment of carcinoma of the low rectum compared with open surgery.
**Primary outcome measure**	The 3-year disease-free survival (DFS) rate
**Secondary**	● Pathologic outcomes

**outcome measures**	● 30-day postoperative complications<br>● 30-day postoperative mortality<br>● Overall survival<br>● Locoregional recurrence rate
**Inclusion criteria**	1) Aged 18-75 years;<br>2) Pathological diagnosis of rectal adenocarcinoma (including highly and moderately differentiated tubular adenocarcinoma, papillary adenocarcinoma, poorly differentiated tubular adenocarcinoma, mucinous adenocarcinoma, and signet-ring cell carcinoma);<br>3) The lower margin of the tumor is < 5 cm from the dentate line at the time of initial diagnosis by rigid proctoscopy;<br>4) cT1-3N0-2M0 or cT4aN0-2M0 adenocarcinoma after neoadjuvant chemoradiotherapy. Patients with pelvic lateral lymph nodes are ineligible;<br>5) No other concurrent primary cancers;<br>6) Primary tumor < 6 cm in size;<br>7) Adequate function of main organs, allowing surgical treatment;<br>8) Patients and their family members can understand the study plan, are willing to participate, and agree to give written informed consent.
**Exclusion criteria**	1) Aged < 18 or > 75 years;<br>2) Concurrent or previous malignancies within five years;<br>3) Need for emergency surgery due to intestinal obstruction, intestinal perforation, intestinal hemorrhage, etc. ;<br>4) Previous history of colorectal surgery that might affect the reconstruction of the digestive tract;<br>5) Need to remove other organs in addition to the rectum;

	6) ASA classification IV or V;<br><br>7) Current pregnancy or lactation:<br><br>● Women of childbearing age with a positive pregnancy test at baseline or who have not taken a pregnancy test; postmenopausal women must be at least 12 months postmenopausal<br><br>● Sexually active men and women (of reproductive age) who are unwilling to take contraceptive measures during the study period<br><br>8) Severe mental illness;<br><br>9) Inability to tolerate surgery due to severe emphysema, interstitial pneumonia, ischemic heart disease, etc.;<br><br>10) Continuous systemic steroid therapy within the last month;<br><br>11) Contraindications to laparoscopic surgery;<br><br>12) Patients and their family members cannot understand the conditions and objectives of this study.
**Sample size estimation**	The sample size was estimated using a log-rank test based on a 3-year DFS with a noninferiority margin of 10%. At the time of this trial design, a noninferiority margin of 10% was determined to be clinically acceptable. Considering distinct prognoses in patients with stage I versus II/III disease, the sample size was calculated separately. Assumptions for the 3-year DFS rate in the open surgery group were 94.3% and 75.2% for clinical stage I and II/III, respectively, with 359 patients with stage I disease (laparoscopic 240, open 119), 609 patients with stage II/III disease (laparoscopic 406, open 203), and a total of 968 patients (laparoscopic 646, open 322) were required to provide an 80% power with a one-sided α of 2.5%. 1065 patients were aimed to enroll to allow for exclusions after randomization to maintain the required statistical power. All calculations allowed for a 20% dropout. The planned and follow-up period were three years and five years, respectively.
**Treatment plan**	**1. Surgery:**<br>● Patients with cT1-2N0M0 tumors are randomized to undergo laparoscopy-assisted resection or open resection.

	● Patients with cT3N0, cT1-3N1-2M0, or cT4aNany-M0 rectal adenocarcinoma are randomized to undergo laparoscopy-assisted or open resection after 6-8 weeks of preoperative concurrent chemoradiotherapy.<br><br>**2. Preoperative chemoradiotherapy:**<br><br>● Preoperative chemoradiotherapy was indicated for patients with clinical stage II/III rectal cancer.<br><br>● Preoperative radiotherapy consisted of a dose of 45-50.4 Gy in 25 or 28 fractions.<br><br>● Concurrent chemotherapy was based on 5-fluorouracil (5-FU) or analogs.<br><br>● An addition of preoperative capecitabine chemotherapy (1250 mg/m^2, twice a day for 14 days) was performed between the end of radiotherapy and surgery.<br><br>**3. Adjuvant therapy:**<br><br>Adjuvant chemoradiotherapy or chemotherapy is recommended as the NCCN guideline.<br><br>● Chemotherapy regimens based on 5-FU or its analogs are chosen. CapeOx or single-agent Capecitabine chemotherapy is recommended. For concurrent chemoradiotherapy, single-agent capecitabine chemotherapy is recommended. Chemotherapy should not exceed 6 months.<br><br>● The radiotherapy protocols used in this study follow the principles of preoperative radiotherapy.

1. Background

Based on the World Health Organization (WHO) report in 2005, there are 400,000 new colorectal cancer patients every year in China. Colorectal cancer has become the third-leading cause of cancer-related death in Chinese patients, with an annual mortality rate of 16.83 per 100,000. Low and mid rectal cancers account for approximately 70% to 80% of rectal cancers in China. Total mesorectal excision (TME) is still the primary treatment for rectal cancer. Recent studies have shown that laparoscopy-assisted resection is safe to treat low rectal cancer. It has the advantages of minimally invasive procedures: faster postoperative recovery, less pain, shorter hospital stay, and long-term efficacy comparable to open surgery.

Randomized control trials with large samples were launched to investigate laparoscopic and open surgery for colorectal cancer in European and North American countries, including the CLASSIC trial, the COLOR trial, and the COST study. These studies compared the completeness of tumor resection, long-term efficacy, and patient's Quality of life (QoL) between the two approaches of surgery. The results showed that laparoscopic resection did not increase complications or operative mortality compared with open resection in treating rectal cancer. The two surgeries were comparable in the distance and negative rate of pathological resection margins (proximal, distal, and circumferential) and the number of dissected lymph nodes. Many meta-analyses have found no significant differences between the two surgical approaches in local recurrence rate, distant metastasis rate, or disease-free survival (DFS) rate.

Recent studies have also shown that laparoscopic total mesorectal excision (TME) for rectal cancer after preoperative chemoradiotherapy is safe without increasing the incidence of surgery-related complications or mortality. Moreover, with the accumulation of surgical experience and skills, the conversion rate from laparoscopic surgery to open surgery gradually decreases. However, the interpretation of these findings needs to be further discussed.

The 2013 National Comprehensive Cancer Network (NCCN) Guidelines v.4 for colon cancer recommended laparoscopic surgery to treat colon cancer, which should be performed by experienced surgeons. However, limited by the lack of data from randomized controlled studies with large samples, the 2013 NCCN Guidelines v.4 only recommended laparoscopic radical resection of rectal cancer for clinical research.

TME has been recognized as the "gold standard" for radical resection of mid and low rectal cancer. Due to the unique anatomical characteristics of low rectal cancer (within 5 cm from the dentate line), open surgery cannot avoid the disadvantage of the limited exposure to the pelvic floor. In contrast, the 30° lens of the laparoscope has

advantages in visualizing the deep pelvic structure; in patients with low rectal cancer, the pelvic operating field can be visualized to ease the separation and nudization of the lower end of the rectum. The benefits of neoadjuvant chemoradiotherapy for patients with resectable cT3/T4aN0 M0 or cT1-4aN1-2M0 low-rectal cancer have not been fully confirmed. In these patients, the safety and long-term efficacy of laparoscopic surgery still need to be evaluated and compared with open surgery.

The search for studies related to laparoscopic resection for rectal cancer at ClinicalTrials.gov, an international clinical trial registry platform, reveals that there are seven ongoing large-scale prospective randomized controlled trials concerning laparoscopic resection for rectal cancer across the world (Table 1). However, there is no randomized controlled trial of laparoscopic surgery, especially addressing low rectal cancer.

Thus, we designed this trial to assess the perioperative safety and long-term efficacy of laparoscopic surgery for low rectal cancer compared with open surgery. The findings from this study will provide high-level evidence for clinical decision-making for surgical treatment in patients with low rectal cancer.

Table 1. Randomized Clinical Trials of Laparoscopy-Assisted Surgery for Rectal Cancer

Trial	Country	Trial registration	Tumor location	Tumor stage	Chemoradiotherapy	The anticipated number of patients enrolled	Randomization ratio	Primary endpoint	Secondary endpoint
-	China	NCT00601549	Below the peritoneal reflection	Progressive stage	Concurrent chemoradiotherapy	600	1:1	Perioperative recovery indicators; oncological outcomes	Recovery indicators at six months after the operation
-	Spain	NCT00782457	Within 0-11 cm from the anal verge	T1-T3	Capecitabine combined with radiotherapy (50-54 Gy)	200	1:1	Perioperative recovery indicators; oncological outcomes	Long-term efficacy
MRC CLASSIC	Britain	NCT00003354	Colorectal	Unspecified	Chemoradiotherapy	1200	2:1	3-year local recurrence rate	3-, 5-, 7-year DFS rates and OS rates
COLOR II	Europe	NCT00297791	Within 15 cm from the anal verge	T1-T3	Undisclosed	1100	2:1	Local recurrence rate within three years after the operation	3-, 5-, 7-year DFS rates and OS rates
COREAN	South Korea	NCT00470951	Within 9 cm from the anal verge	Unspecified	Chemoradiotherapy	340	1:1	3-year DFS rate	Quality of life (QoL) and anorectal function within one year after the operation
ACOSOG-Z6051	USA, Canada	NCT00726622	Within 12 cm from the	IIA, IIIA, IIIB	5-FU-based chemotherapy/radiot	650	Undisclosed	Oncological outcomes	disease-free survival and rate of local

			anal verge		herapy				recurrence, as well as Quality of life(QoL)
ALaCaRT	Australia and New Zealand	ACTRN12609000663257	T1-T3 rectal cancer ≤15 cm from anal verge	T1-T3anyNanyM	5-FU-based chemotherapy/radiation	470	1:1	Successful resection, including complete mesorectal excision and negative circumferential and distal margins	Perioperative safety, oncological outcomes

2. Objective

To evaluate the perioperative safety and long-term efficacy of laparoscopy-assisted surgery for low rectal cancer compared to open surgery.

3. Participants

Eligible participants are the patients who have an initial diagnosis of rectal cancer within 5 cm from the dentate line (by rigid proctoscopy). Clinical staging is determined by computed tomography (CT), endorectal ultrasound, and pelvic magnetic resonance imaging (MRI). No distant metastasis can be detected. No contraindication to general anesthesia. The participating patients have cT3/T4aN0M0 or CT1-4aN1-2M0 low rectal cancer without contraindications to preoperative radiotherapy or chemotherapy. Low rectal cancer is defined as a rectal adenocarcinoma with a lower margin within 5 cm from the dentate line as determined by rigid proctoscopy at diagnosis.

3.1 Inclusion criteria

1) Aged 18-75 years;
2) Pathological diagnosis of rectal adenocarcinoma (including highly and moderately differentiated tubular adenocarcinoma, papillary adenocarcinoma, poorly differentiated tubular adenocarcinoma, mucinous adenocarcinoma, and signet-ring cell carcinoma);
3) The lower margin of the tumor is < 5 cm from the dentate line at the time of initial diagnosis by rigid proctoscopy;
4) cT1-3N0-2M0 or cT4aN0-2M0 adenocarcinoma after neoadjuvant chemoradiotherapy. Patients with pelvic lateral lymph nodes are ineligible;
5) Primary tumor < 6 cm in size;
6) No other concurrent primary cancers;
7) Adequate function of main organs, allowing surgical treatment;
8) Patients and their family members can understand the study plan, are willing to participate, and agree to give written informed consent.

3.2 Exclusion criteria

1) Aged < 18 or > 75 years;
2) Concurrent or previous malignancies within five years;
3) Need for emergency surgery due to intestinal obstruction, intestinal perforation, intestinal hemorrhage, etc. ;
4) Previous history of colorectal surgery that might affect the reconstruction of the digestive tract;
5) Need to remove other organs in addition to the rectum;
6) ASA classification IV or V;

7) Current pregnancy or lactation:

- Women of childbearing age with a positive pregnancy test at baseline or who have not taken a pregnancy test; postmenopausal women must be at least 12 months postmenopausal;
- Sexually active men and women (of reproductive age) who are unwilling to take contraceptive measures during the study period;

8) Severe mental illness;

9) Inability to tolerate surgery due to severe emphysema, interstitial pneumonia, ischemic heart disease, etc.;

10) Continuous systemic steroid therapy within the last month;

11) Contraindications to laparoscopic surgery;

12) Patients and their family members cannot understand the conditions and objectives of this study.

4. Study design

This study is a prospective, multicenter, randomized, open-label, parallel-group, noninferiority clinical trial.

5. Sample size estimation

The sample size was estimated using a log-rank test based on a 3-year DFS with a noninferiority margin of 10%. At the time of this trial design, a noninferiority margin of 10% was determined to be clinically acceptable. Considering distinct prognoses in patients with stage I versus II/III disease, the sample size was calculated separately. Assumptions for 3-year DFS rate in the open surgery group were 94.3% and 75.2% for clinical stage I and II/III, respectively,20,21 359 patients with stage I disease (laparoscopic 240, open 119), 609 patients with stage II/III disease (laparoscopic 406, open 203), and a total of 968 patients (laparoscopic 646, open 322) were required to provide an 80% power with a one-sided α of 2.5%. One thousand sixty-five patients were aimed to enroll to allow for exclusions after randomization to maintain the required statistical power. All calculations allowed for a 20% dropout. The planned and follow-up period were three years and five years, respectively.

6. Intervention

6.1 Preoperative chemoradiotherapy

Patients with cT3/T4aN0M0 or cT1-4aN1-2M0 low rectal carcinoma undergo preoperative chemoradiotherapy before surgery and undergo surgery 6 to 8 weeks after completing the radiotherapy.

6.1.1 Preoperative concurrent chemotherapy protocol

According to the NCCN Guidelines (2013, v.4), for the treatment of rectal cancer, chemotherapy regimens based on 5-FU or its analogs are chosen. A regimen of capecitabine (825 mg/ m^2, twice a day, 5-7 days a week for five weeks) is recommended.

6.1.2 Preoperative radiotherapy protocol

(1) Definition of target areas: The primary tumor and regional lymphatic drainage area's high-risk recurrence area must be irradiated. The high-risk recurrence areas of primary tumors include the tumor/tumor bed, the mesorectal region, and the presacral region. The target area for radiation treatment of carcinoma of the low rectum should include the ischiorectal fossa.

The regional lymphatic drainage areas include the lymphatic drainage area of the common iliac vessels in the true pelvis, the mesorectal region, the lymphatic drainage area of the internal iliac vessels, and the obturator lymph node area.

Radiation therapy techniques: According to the radiotherapy equipment available in the hospital, different techniques can be chosen, including conventional radiotherapy, three-dimensional (3D) conformal radiotherapy, intensity-modulated radiotherapy (IMRT), and image-guided radiotherapy.

CT simulation is recommended for radiotherapy treatment planning. If CT simulation is unavailable, conventional simulation must be performed. Patients should be placed in the prone or supine position with a full bladder.

Patients must receive irradiation in three or more fields.

If IMRT is necessary, the radiation treatment plan must be validated.

Local dose enhancement can be achieved through intraoperative or external irradiation.

Irradiation dose: The irradiation dose and schedule must be clearly defined regardless of which radiation therapy technology (conventional radiotherapy or new techniques, such as 3D conformal radiotherapy and IMRT) is used. The volumetric dose is defined for 3D conformal radiotherapy planning and IMRT, while the isocenter dose should be defined for conventional radiotherapy planning.

The protocol recommended for the high-risk recurrence area of the primary tumor and the regional lymphatic drainage area is a total dose (DT) of 45.0-50.4 Gy in 1.8-2.0 Gy per fraction for 25 or 28 fractions.

6.1.3 Preoperative chemotherapy during the interval between preoperative chemoradiotherapy and surgery

As recommended by the academic advisory committee, an addition of preoperative capecitabine chemotherapy (1250 mg/ m^2, twice a day for 14 days) was performed between the end of radiotherapy and surgery.

6.2 Surgical treatment plan

Surgery will be performed 6 to 8 weeks after the end of radiotherapy.

6.2.1 Open surgery

This surgery includes standard TME and dissection of the No. 253 lymph nodes. The inferior mesenteric artery (IMA) is ligated either from its base directly or after the branching of the left colon artery. The rectum's distal resection margin (DRM) is at least 1-2 cm away from the tumor. The surgical method is chosen as follows:

(1) Transabdominal low anterior resection (LAR) or ultralow anterior resection (ULAR) may be chosen if the lower margin of the tumor is ≥ 2 cm from the proximal side of the anorectal ring.

(2) In cT1 and T2 patients, as well as T3 patients whose external anal sphincters are not invaded by the tumor (including patients who have undergone neoadjuvant therapy), intersphincteric resection (ISR) through either the transpelvic approach or transanal approach may be chosen when the lower margin of tumor is < 2 cm from the proximal end of the anorectal ring.

(3) cT1 to T3 tumors that are ≥ 5 cm from the anal verge are confined to the intestinal wall within a depth of ≤3 cm and affect 1/2 of the circumferential wall of the rectum without invading the bladder, prostate, or vagina can be treated with ULAR via a pull-through procedure.

(4) Abdominoperineal resection (APR) can be performed in patients with external anal sphincter invasion.

(5) Extralevator abdominoperineal excision (ELAPE) can be performed via the pelvic approach in patients with levator ani muscle invasion.

For patients receiving sphincter preservation or partial sphincter preservation, loop transverse colostomy or ileostomy is recommended. See "9.6.1 Open surgery" for the detailed surgical procedure.

6.2.2 Laparoscopic surgery

The indications of laparoscopic operation are the same as those of open operation. The operation follows the principle of TME and includes the dissection of the No. 253 lymph nodes. The IMA is ligated either from its base directly or after the branching of the left colon artery. The rectal DRM should be at least 1 to 2 cm away from the tumor. The principles for

selecting the intestinal segmental resection and/or reconstruction methods are the same as in "**6.2.1 Open surgery**". Loop transverse colostomy or ileostomy is recommended for patients who undergo sphincter-preserving surgery. See "**9.6.2 Laparoscopic surgery**" for the detailed surgical procedure.

6.3 Adjuvant therapy

Radiotherapy and chemotherapy are planned according to the postoperative pathological staging results. The details are presented in "**9.8 Adjuvant therapy**".

7. Follow-up

- Medical history is collected, and a physical examination is performed every three months for two years, then every six months for three years, and every year after five years.
- Carcinoembryonic antigen (CEA) and carbohydrate antigen 19-9 (CA19-9) are measured every three months for two years, then every six months for three years, and every year after five years.
- Abdominal/pelvic ultrasound and chest X-ray examinations are done every three months for two years, then every six months for three years, and every year after five years.
- An abdominal/pelvic MRI or non-contrast-enhanced plus contrast-enhanced CT scan is performed once a year. For patients who are allergic to contrast agents, non-contrast-enhanced CT alone is an alternative.
- Colonoscopy once a year; if a full preoperative colonoscopy could not be performed due to obstructing lesion, colonoscopy should be performed in 3-6 months after surgery
- Quality of life (QoL) is evaluated with the EORTC QLQ C30 and EORTC QLQ CR29 at three months, six months, and 12 months after surgery.
- Bladder function is assessed at five days, three months, six months, and 12 months after surgery.
- Sexual function is assessed at three months, six months, and 12 months after surgery.
- An anorectal function is assessed 12 months after surgery.
- Colonoscopy is performed within one year after surgery. Patients who show abnormal findings in the first examination must be examined again within one year. Patients who do not have polyps in the first examination will receive colonoscopy again three years and afterward once every five years. It is recommended to remove any colorectal adenomas found in follow-up examinations.

8. Study endpoints

8.1 Primary outcome measure

3-year DFS rate (final calculation is done 3 years after the last participant is enrolled)

8.2 Secondary outcome measures

■ Pathologic outcomes

Pathologic outcomes are defined as TME quality, negative CRM and negative DRM, length of proximal resection margin (PRM), length of DRM, and the number of retrieved lymph nodes.

■ 30-day postoperative complications

■ 30-day postoperative mortality

■ 3-year and 5-year OS rates (calculated 3 and 5 years after the last participant is enrolled)

■ Locoregional recurrence rate

8.3 Other pre-specified outcome measures

■ Operative outcomes:

1) Operation time (min)
2) Estimated blood loss (ml)
3) Conversion to open surgery (only applicable to the laparoscopic resection group)
4) Events of intraoperative complications

■ Postoperative recovery outcomes

1) Time to first flatus (h)
2) Time to the first defecation (h)
3) Time to first liquid food (h)
4) Time to normal diet (h)
5) Duration of analgesic use (h)
6) Length of postoperative hospital stay (d)

■ The Quality of life (QoL)

1) Quality of life (QoL) assessment: the EORTC QLQ C30 and EORTC QLQ CR29 scores

2) Bladder function assessment:

- The residual urine volume (1st measurement: after the urethral catheter is removed on the fifth day after the operation, the residual urine volume in the bladder is determined by B-ultrasound after the patient urinates as much as possible for the first time)
- I-PSS

3) Patient self-reported sexual function

Patient self-reported sexual function as assessed by the International Index of Erectile Function (IIEF-5) Female Sexual Function Index(FSFI) for male and female sexual function， respectively.

8.4 Definition of outcome measures

8.4.1 Disease-free survival (DFS)

DFS is defined as the period from the surgery date to the date of tumor recurrence or death (whichever comes first). In patients to whom this definition is not applicable, the last evaluation date is used for analysis. In addition, patients whose treatment is terminated without confirmation of tumor recurrence or metastasis should be followed up radiographically after the termination of treatment.

The date of recurrence is defined as when obtaining a definitive diagnosis.

Tumor recurrence is diagnosed when any of the conditions below occurs:

- Clear evidence showing tumor metastasis to the pelvis and pelvic lateral lymph nodes or inguinal lymph nodes
- Clear evidence showing metastatic lesions in the liver, lung, bone, etc.
- Death from any cause

Note: Without objective evidence (radiography/pathology), an increase in tumor markers alone or unexplained clinical deterioration does not necessarily indicate tumor recurrence.

8.4.2 Overall survival (OS)

OS is defined as the time from the date of randomization to death from any cause. For the patients who are alive at the end of the study, the date of the last contact is used for analysis.

8.4.3 Pathologic outcomes

Pathologic outcomes are defined as TME quality, negative CRM and negative DRM, length of proximal resection margin (PRM), length of DRM, and the number of retrieved lymph nodes.

8.4.4 30-day postoperative complications

Thirty-day postoperative complications included any complications occurring within 30 days after surgery. Postoperative complications were graded according to the Clavien-Dindo classification. Severe complications were defined as Clavien-Dindo III-V.

8.4.5 30-day postoperative mortality

Thirty-day operative mortality is defined as deaths occurring from any cause during the first 30 postoperative days.

8.4.6 Operative outcomes

- **Conversion to Open Surgery**

The conversion is defined as any part of the mesorectal dissection using the traditional open surgery. The surgeon decided on the conversion after considering patient safety, technical diffficulties, and relevant conditions influencing the completion of TME.

- **Operative time**

Operative time is defined as the time from skin incision to the completion of skin suture.

- **Estimated blood loss**

Estimated blood loss will be measured according to the suction and the weight of wet gauze, and then minus the irrigation.

- **Intraoperative outcomes**

Intraoperative outcomes are defined as events occurring during surgery and details of the surgical procedure, such as conversion, operative time, estimated blood loss, type of surgery, and diverting ostomy creation.

8.4.7 Postoperative outcomes

- **Time to first flatus**

Time to first flatus is defined as the time from surgery completion to the first occurrence of flatus during the subject's postoperative recovery (measured in hours).

- **Time to the first defecation**

Time to the first defecation is defined as the time from skin closure to the time of time to first defecation (measured in hours)

- **Time to the liquid diet**

Time to a liquid diet is defined as the time from surgery completion to the resumption clear liquid diet (measured in hours)

- **Time to the normal diet**

Time to normal diet is defined as the time from surgery completion to the resumption of the normal diet (measured in hours)

- **Length of hospital stay**

Duration of hospital stay is measured from the day of surgery until the day of discharge from the hospital

9. The flow of study

An academic advisory committee supervised by the principal investigator of the study will be established.

Before starting the study, surgeons participating in the study will receive training on standard operating procedures and a surgical training certificate provided by the academic advisory committee. All hospitals participating in this study shall provide copies of qualification certificates/licenses and personal legal certificates. All materials shall be bound together for document management. All doctors participating in this study shall provide copies of the qualification certificates, which will be documented.

9.1 Randomization

This study uses a dynamic randomization technique based on variance minimization. Patients are stratified by clinical tumor stage (stage I, II, or III), age (≤ 44 years, 45-59 years, or ≥ 60 years), sex, body mass index (BMI) (≤ 23.9 kg/ m^2, 24-27.9 kg/ m^2, or ≥ 28 kg/ m^2), and ASA classification (I, II, or III). Random numbers are generated and assigned to patients by a computerized central randomization system called the Distributed Annotation System (DAS) for interactive web response system (IWRS) for treatment randomization following the principle of central competition. After the last participant has been assigned a random number, the researcher responsible for randomization derives the randomization table from the randomization system and submits it to the sponsoring organization. Two sealed copies of each randomization table are stored in the national institution on clinical trials of drugs to which the principal investigator belongs and the sponsoring organization.

After each participant is approved as eligible, a researcher logs in to the DAS for IWRS, enters the participant's information, including initials, sex, and age, applies for randomization into the treatment group, and then fills the random number into the medical record of the study.

9.2 Screening of participants

All participants must have a definitive histopathological diagnosis. Their tumors must have been clinically staged through pelvic MRI or, if possible, the combined use of pelvic MRI and endorectal ultrasound. Patients who meet the inclusion criteria and cannot be excluded are considered potential participants based on the exclusion criteria.

Stage I patients: The doctors in charge inform the participants and their families about the study, explain the differences between the two surgical approaches, and ask whether they agree to be randomized into one of the two groups. Patients who agreed to be randomized signed informed consent and were enrolled in the study.

Stage II or III patients: After filling out **The Participant Screening Form,"** patients are treated with preoperative chemoradiotherapy and given preoperative examinations 6-7 weeks after the completion of radiotherapy. Patients who fully meet the inclusion criteria and cannot be excluded are considered potential participants based on the exclusion criteria. The doctors in charge informed the participants and their families about the study explained the differences between the two surgical approaches, and asked whether they agreed to be randomized into one of the two groups patients who agreed to be randomized signed informed consent and were enrolled in the study.

9.3 Inclusion of participants

Only the patients who fully meet the inclusion criteria will be included in the study; those who meet any exclusion criteria will not be included.

9.4 Management of patient participation

Five days before randomization, patients who meet the inclusion criteria complete the following examinations and questionnaires:

1) Complete blood count, urinalysis, stool analysis, fecal occult blood tests
2) Biochemical examination (including liver and kidney function, electrolytes, blood glucose, and blood lipid levels), blood type, coagulation function, digestive tract tumor markers (CEA and CA199), screening for infectious diseases (hepatitis B, hepatitis C, AIDS, syphilis, etc.)
3) X-ray examination or non-contrast-enhanced chest CT scan; contrast-enhanced scan if needed
4) Electrocardiogram (ECG)
5) Enhanced liver/pelvic MRI scan, which can be combined with endorectal ultrasound or endoscopic ultrasound if possible
6) Echocardiography, pulmonary function, and other examinations according to the patient's condition if necessary
7) Serum or urine pregnancy tests in women of childbearing age
8) Bladder function assessment: the residual urine volume in the bladder and the I-PSS
9) Quality of life (QoL) assessment: EORTC QLQ C30 and EORTC QLQ CR29
10) Sexual function assessment: the IIEF-5 and the FSFI
11) Assessment of anorectal function (for those who plan to preserve or partially preserve the anal sphincter): Anorectal pressure, rectal sensory function, anorectal reflexes, and fecal incontinence (Wexner score)

9.5 Preoperative preparation

1) Anesthesia: general anesthesia with endotracheal intubation.
2) Intestinal preparation: An oral laxative is administered 12-24 hours before the operation and finished within 2 to 3 hours.
3) Prophylactic use of antibiotics: Antibiotics are intravenously administered 0.5 to 2 hours before surgery or at the beginning of anesthesia, and a second dose can be given more than 3 hours after surgery.

4) Other preoperative preparations are performed routinely.

9.6 Surgical procedures

9.6.1 Open surgery

Standard TME and dissection of No. 253 lymph nodes:

For patients receiving sphincter preservation or partial sphincter preservation, loop transverse colostomy or ileostomy is recommended.

1) Anesthesia: general anesthesia with endotracheal intubation.

2) Abdominal incision: An incision is made around the umbilicus in the middle of the lower abdomen.

3) Exploring the abdominal cavity: The abdominal exploration is performed from far to near" and from the presumed typical structures to the tumor site. The purpose is to clarify whether tumor metastasis has occurred in the liver, the peritoneum, or the lymph nodes near the para-abdominal aorta, the mesenteric artery, and the internal iliac artery tumor has infiltrated into the bladder, the prostate, or the uterus, and its appendages.

4) Inferior mesenteric vessels: After the inferior mesenteric vessels are separated, the IMA is ligated either from its base directly or after the branching of the left colon artery. Subsequently, the No. 253 lymph nodes are dissected.

5) Separation of the sigmoid colon and rectum: The sigmoid colon and its mesentery are separated along with the space of the left Toldt's fascia, with particular special attention paid to protecting the ureter and gonadal vessels. Sharp dissection is performed along with the space between the mesorectal fascia and the parietal pelvic fascia following the posterior order, lateral, and anterior. In male patients, the anterior rectal wall is separated from the seminal vesicle along with the space of Denonvilliers' fascia. In female patients, the anterior wall of the rectum is separated at the level of the rectogenital septum.

Particular attention should be paid to protecting the pelvic autonomic nerves during the lateral and anterolateral rectum separation. Finally, the rectum is mobilized down to the level of the levator ani muscle.

The surgical procedures for segmental resection and/or reconstruction of the rectum are as follows.

9.6.1.1 Low or ultralow anterior resection (LAR or ULAR)

After excision and nakation of the mesorectum, the rectum is transected at 2 cm below the margin of the tumor using a Contour cutter stapler. The sigmoid colon is transected at 10 cm proximal to the tumor, and a mechanical end-to-end anastomosis of the proximal colon and the rectal stump is performed.

For ULAR via a pull-through procedure, the procedure is recommended as follows: after the rectum is fully mobilized, the sigmoid colon is transected at 10 cm proximal to the tumor using an endoscopic linear cutter stapler under laparoscopy. Next, after the sigmoid colon stump is grasped with grasping forceps and passes the anus, the proximal sigmoid colon and rectum, including the tumor, are gently reversed and pulled out from the anus through the intestinal canal. As a result, the rectal segment out of the body through the anus has a mucosal

surface turned inside-out. From the outside of the body, the rectal segment is transected at a sufficient resection margin≥ 2 cm from the distal end of the tumor by using a linear cutter stapler under direct vision. Finally, the rectal stump is returned into the pelvic cavity through the anus, and intestinal anastomosis is performed using a tubal anastomosis device.

9.6.1.2 Intersphincteric resection (ISR)

- **Transabdominal ISR**

The rectum is continuously mobilized along with the space between the rectum and the levator hiatus. It is transected with the Contour cutter stapler when the separation enters the space between the internal and external sphincters down to 2 cm below the tumor margin. Then the sigmoid colon is transected at 10 cm proximal to the tumor, and an end-to-end anastomosis of the proximal colon and the rectal stump or the anal canal is performed.

- **Transanal ISR**

During the perineal resection, the mucosa and submucosa of the anal canal are cut open at 0.5 cm above the dentate line under direct vision. After the cut reaches the space between the internal and external sphincters, a dissociation is made upward along the gap until it meets the area separated from the abdominal side. A portion of the internal sphincter above the dentate line and the rectum are resected while the external anal sphincter is fully preserved. The proximal colon is pulled out of the anus through the external sphincter tunnel. Then the sigmoid colon is transected at 10 cm proximal to the tumor, followed by a coloanal anastomosis above the dentate line.

9.6.1.3 Abdominoperineal resection (APR)

- **Conventional APR**

After purse-string suturing the anus during the perineal resection, a fusiform incision is made around the anus, 3 cm away from the anus. The anterior end of the incision reaches the midpoint of the perineum. The posterior end reaches the tip of the coccyx, and both sides reach the medial surface of the ischial tuberosity. In female patients, the posterior vaginal wall can be partially resected if the tumor has infiltrated the posterior vaginal wall. Next, the fat in the ischiorectal space is removed until below the levator ani muscle along the anterior surface of the gluteus maximus, the medial surface of the ischial tuberosity, and the posterior surface of the superficial transverse perineal muscle. Then the anococcygeal ligament is dissected, and the posterior levator ani muscles are cut near the pelvis. After the iliococcygeal and pubococcygeus muscles are cut open anterolaterally up to the lateral surface of the puborectalis muscle, the central tendon, the perineum, the puborectalis, and rectourethral muscles are dissected along with the posterior margin of the superficial transverse perineal muscle. Finally, the pelvic floor peritoneum is sutured without tension, and the perineal incision is sutured, followed by a permanent colostomy at the proximal colon.

- **Extralevator abdominoperineal excision (ELAPE)**

The separation of the rectum stops at the starting point of the levator ani muscle (tendinous arch of the levator ani) without reaching the level of the levator ani muscle or the mesorectum. The levator ani muscles at both sides are cut vertically from the medial side of the tendinous arch of the levator ani to the pelvic floor until the two cuts meet at the tip of the coccyx and then go past the coccyx. Next, the sigmoid colon is transected at 10-15 cm proximal to the tumor, followed by permanent colostomy at the proximal colon. In the perineum group, the separation is performed along with the space between the levator ani muscle and the fatty tissue in the ischioanal space. It continues until it reaches the levator ani muscle dissected during the pelvic operation, connecting the separated areas in the pelvis and perineum.

9.6.2 Laparoscopy-assisted surgery

The indications for the surgery are described in "**6.2.2 Laparoscopic Surgery**". Prophylactic loop transverse colostomy or ileostomy is recommended for all patients who plan to preserve the anal sphincter.

Surgical technique:

1) Anesthesia: As in Open surgery, general anesthesia with endotracheal intubation is commonly used.

2) Position: The patient is placed in the lithotomy position with legs abducted 30° from the midline.

3) Establish pneumoperitoneum: puncture establishes Pneumoperitoneum at or 1 cm above the umbilicus, and the abdominal pressure is kept at 12-15 mmHg.

4) Port site selection: The based on their operation habits.

5) Abdominal exploration: The abdominal cavity is explored from far to near, the lesion site being the last. The exploration is conducted generally in the peritoneum → liver → stomach, gallbladder, pancreas → greater omentum → small intestine → large intestine (except for the tumor site) → the pelvic cavity, and its organs → lymph nodes at the roots of the supplying vessels → primary tumor.

6) The inferior mesenteric vessels: Either the medial or lateral approach can be chosen according to the surgeon's experience. After the bifurcation of the abdominal aorta is identified, the mesentery is mobilized upward along the abdominal aorta from the level of the sacral angle. Then nude inferior mesenteric artery and vein(IMA/IMV). IMA can be ligated at the root or after the branching of the left colonic artery, and the No. 253 lymph nodes are dissected.

7) Mobilization of the sigmoid colon and rectum: The sigmoid colon and its mesentery are mobilized along with the space of the left Toldt's fascia, with particular attention paid to protecting the ureter and gonadal vessels. Sharp dissection is performed along with the space between the deep rectal fascia and the parietal pelvic fascia in the posterior, lateral, and anterior order. In male patients, the anterior rectal wall is separated from the seminal vesicle along with the space of Denonvilliers' fascia. In female patients, the anterior rectal wall is mobilized down to the level of the rectogenital septum. Particular attention should be paid to protecting the pelvic autonomic nerves during the separation of the lateral rectum from its

lateral space. Finally, the rectum should be transected down to the level of the levator ani muscle.

The surgical procedures for segmental resection and/or reconstruction of the rectum are as follows.

9.6.2.1 Laparoscopy-assisted Low or ultralow anterior resection (LAR or ULAR)

After excision and nakation of the mesorectum, the rectum is transected at 2 cm below the margin of the tumor using an endoscopic cutter stapler. The sigmoid colon is transected at 10 cm proximal to the tumor, and an end-to-end anastomosis of its proximal colon and the rectal stump is performed mechanically.

For ULAR via a pull-through procedure, the procedure is recommended as follows: after the rectum is fully mobilized, the sigmoid colon is transected proximal to the tumor (ideally, the stump can be pulled out through the anus by 5 cm) using an endoscopic linear cutter stapler under laparoscopy. After the rectal stump is grasped with grasping forceps passing the anus, the severed rectum, including the tumor, is gently reversed and pulled out from the anus through the intestinal canal. As a result, the rectal segment pulled out of the body from the anus has the mucosal surface turned inside out. Next, from the outside of the body, the rectal segment is transected at a sufficient resection margin using a linear cutter stapler under direct vision. Finally, the rectal stump is returned into the pelvic cavity through the anus, and an anorectal anastomosis is performed.

9.6.2.2 Laparoscopy-assisted ISR

- **Transabdominal ISR**

After separating the rectum until the levator hiatus, anal palpation is conducted to confirm that the lower margin of the tumor is within 2 cm of the levator hiatus. The rectum is then sharply mobilized from the surface of the longitudinal rectal muscle along the medial margin of the puborectalis muscle, and the hiatal ligament is cut off posteriorly. After the separation is conducted along with the sphincter space, a lower tumor margin ≥ 2 cm must be confirmed by anal palpation again to ensure that the cutter stapler can be used.

- **Transanal ISR**

The transabdominal approach described above is converted to the transanal approach if the separation of the sphincter space is difficult, if the rectum segment below the tumor cannot reach the length ≥ 2 cm even after great efforts at separation have been made, or if the pelvis is too narrow to use a cutter stapler. Disinfection of the perineum is immediately performed, followed by placement of surgical drapes and cleaning of the rectum with diluted iodophor. Circular retractors are placed, and the diluted epinephrine solution is injected above the dentate line between the internal and external sphincters. After annularly transecting the internal sphincter at 0.5 cm above the dentate line, mobilization is performed upward along with the internal sphincter space until the pelvic cavity. Next, the distal rectum is purse-string

sutured and pulled out through the anus or an abdominal port. Finally, the sigmoid colon is transected at 10 cm proximal to the tumor, followed by a coloanal anastomosis.

9.6.2.3 Laparoscopy-assisted APR

- **Conventional APR**

The perineal operation is the same as Open surgery.

- **Extralevator abdominoperineal excision (ELAPE)**

The mobilization of the rectum stops at the starting point of the levator ani muscle (tendinous arch of the levator ani) without reaching the level of the levator ani muscle or the mesorectum. The levator ani muscles are cut vertically on both sides from the medial side of the tendinous arch of the levator ani to the pelvic floor with an electric knife until the two cuts meet at the tip of the coccyx and then go past the coccyx. The sigmoid colon is transected with a linear cutter stapler under laparoscopy, and a permanent colostomy is performed in the proximal colon. The operation in the perineum group is the same as the traditional APR surgery.

9.7 Intraoperative observation

Complications associated with laparoscopic surgery included subcutaneous emphysema, hypercapnia, vascular and gastrointestinal injuries associated with puncture, gas embolism, etc.

The common complications of laparoscopic and open surgery include:

(1) Presacral hemorrhage

(2) Active intra-abdominal hemorrhage

(3) Injuries to the surrounding organs, such as the ureter and duodenum.

9.8 Adjuvant therapy

9.8.1 Adjuvant chemotherapy

- **Adjuvant chemotherapy indications**

Patients who receive preoperative chemoradiotherapy before surgery continue to receive adjuvant chemotherapy after surgery. According to the NCCN Guidelines (2013, v.4), the chemotherapy regimens based on 5-FU or analogs are chosen to treat rectal cancer. CapeOx or single-agent capecitabine regimens are recommended. Perioperative chemotherapy should not exceed six months.

- **Regimens:**

(1) CapeOx

Oxaliplatin 130 mg/m^2, intravenous infusion over 2 hours, day 1

Capecitabin	1000 mg/m^2, twice a day for consecutive 14 days
	Repeat every three weeks

(2) Capecitabine

Capecitabin	1250 mg/m^2, twice a day for consecutive 14 days, and repeat every three weeks
**Note:**	For postoperative concurrent adjuvant chemoradiotherapy, capecitabine is given at a dose of 825 mg/m^2, twice a day, and 5 to 7 days a week for a total of 5 weeks.

9.8.2 Adjuvant chemoradiotherapy

- Adjuvant chemoradiotherapy is recommended for patients with stage pT3-4 or N1-2 rectal cancer who do not receive preoperative radiotherapy before surgery. According to the NCCN Guidelines (2013, V4 edition), the chemotherapy regimens based on 5-FU or its analogs are chosen to treat rectal cancer. CapeOx regimens or single-agent capecitabine regimens are recommended. For concurrent chemoradiotherapy, a single-agent capecitabine regimen is recommended. Perioperative chemotherapy should not exceed six months. The radiotherapy protocols follow the principles of radiotherapy.

9.9 Postoperative recovery management

Postoperative observation:

- Time requiring analgesics (h)
- Time to first flatus (h)
- Time to first oral intake of liquid food (h)
- Time to return to normal diet (h)
- Time to first bowel movement (h)
- Residual urine volume in the bladder (ml)
- Postoperative complications
- Length of postoperative hospital stay (d)

9.10 Hospital discharge criteria

1) The patient is in good general condition and has returned to a regular diet with normal intestinal function.

2) The patient has an average body temperature and no positive results in abdominal examinations; in other laboratory tests, the results are expected.

3) The abdominal incision and/or the perineal incision shows II/A or II/B healing.

9.11 Variation of discharge time and the analysis of its causes

1) Perioperative complications and/or syndromes require further diagnosis and treatment, leading to a prolonged hospital stay and increased costs.

2) In radical resection of rectal cancer, the resection method of the rectum is chosen according to the tumor location, size, degree of invasion, etc., leading to differences in the postoperative hospital stay.

9.12. Follow-up plan

Same as in "**7. Follow-up plan**".

10. Pathological assessment

10.1 Macroscopic assessment of mesorectal excision

The quality of TME is assessed using a central pathological evaluation method, which is a double-blinded evaluation by two experienced pathologists based on photographs of fresh specimens. According to the evaluation criteria proposed by Nagtegaal et al., the quality of TME is classified into three levels as follows:

(1) Complete: the mesorectum is intact and has a smooth surface with very few irregularities; no defect more than 5 mm in depth; no coning of the distal margin of the specimen; the upper circumferential resection margin (CRM) appears smooth and flat in the slices of the specimen.

(2) Nearly complete: the mesorectum is almost intact, but its surface is irregular; the distal part of the specimen shows moderate coning; the muscularis propria is absent except for the level where the levator ani muscle is inserted; the upper CRM appears moderately irregular in the slices of the specimen.

(3) Incomplete: the mesorectum appears incomplete with defects deep into the muscularis propria, and/or the CRM is extremely irregular.

10.2 Microscopic pathological examination

The local pathologists did the microscopic pathological assessments at the participating centers. Positive resection margin, including circumferential resection margin (CRM) and distal resection margin (DRM), was defined as the presence of cancer cells within 1 mm from the cut edge.

- Pathohistological type and tumor differentiation
- Distance of surgical resection margins (proximal, distal, and circumferential)
- Positivity of surgical resection margins (proximal, distal, and circumferential)
- Numbers of dissected lymph nodes
- Numbers of metastatic lymph nodes
- Tumor regression grade (TRG) in patients undergoing preoperative chemoradiotherapy
- pTNM or ypTNM stage

11. Data management

11.1 Electronic data management

The electronic data management system (DAS) for electronic data capture (EDC) (DAS for EDC) is used to manage all data of this study.

11.1.1 Design of medical record forms

The form for data collection is designed according to the requirements of the study plan. The research workflow, names of the data forms, and the items collected are defined. In addition, data collection guidelines are developed and finalized after they are reviewed and approved by the sponsoring organization.

11.1.2 Construction of electronic case report forms (eCRFs)

The data manager builds the eCRFs according to the study plan and medical record forms.

11.1.3 Management of roles and rights of participating personnel

In the EDC system, the roles and rights of users are both controlled. All users who visit the EDC system must fill out a user account application form. After confirmation and approval by the sponsor, the system administrator creates a project administrator account and gives the administrator rights. Then, the project administrator creates the accounts for researchers, clinical research coordinators (CRC), clinical research quality inspectors, auditors, and data administrators and grants them corresponding permissions to access the EDC system. For example, a researcher can only see and have the rights to revise the data from his/her research center; a sponsor can only browse the EDC system, and a clinical research quality inspector can read the EDC data of all centers and ask questions but do not have the rights to revise the data.

11.1.4 Data entry

The CRC designated by the principal investigator of each center enters the medical record form into the eCRF timely and accurately. The eCRF does not serve as the original record, and its content originates from the medical record forms.

11.1.5 Data verification

The EDC system automatically conducts logic verification during data entry and sends out questions in real-time if the data are problematic. In addition, the data manager will manually verify the text data and ask questions about the problems in the data.

11.1.6 On-site source data verification

At the study site of each center, the clinical research quality inspector can log in to the DAS for EDC and check the consistency of the eCRF data with source data (e.g., medical records) to ensure 100% accuracy. The inspector can send queries online at any time if a problem is found.

11.1.7 Question answering

The investigators answer questions either online in real-time or offline after downloading the question list. The answers given by the investigators offline will be entered into the EDC

system by a CRC. The data administrator and clinical research quality inspector can either accept the answers or ask further questions, if necessary, to ensure that all data are "clean."

11.1.8 Data locking and export

The data administrator will lock the data after (1) all participants have completed the trial, (2) their medical record data have been entered into the system and reviewed by the principal investigators, sponsors, statistical analysts, and data managers; and (3) the database created is confirmed to be correct. After all the data are locked, the administrator imports the data into the designated database for statistical analysis. The locked data cannot be edited again. After approval, a problem is found after data locking can be corrected in the statistical analysis program. If there is definitive evidence that the locked data must be unlocked, the investigators and the sponsor must sign relevant documents before unlocking.

11.1.9 Unblinding

This study is an open-label trial that does not involve unblinding or emergency unblinding.

11.1.10 eCRF archive

After completing the trial, each participant's eCRF and PDF electronic files are generated and stored on CD-ROMs, which will be kept by the organization in charge of the study for seven years after trial completion.

11.1.11 EDC closure

After completing the trial, the data administrator applies for EDC closure and closes the EDC account (i.e., takes it offline) after canceling all account access rights and a complete data backup when permitted by the sponsor. Within seven years after trial completion, the data management center can open the EDC system by appointment if necessary.

11.2 Data management plan

The data management plan is developed and drafted by the data manager according to the trial plan. The data management plan shall serve as a guiding document for the entire data management process and should be followed strictly in terms of times and methods defined during the entire trial.

The data management plan includes:

- Management of the data management plan, including ownership, cover page layout, content list, etc.
- General information of the study, including the purpose of the study, the overall design of the study, etc.
- Schedule of data management work, which includes the start and end times of each step and is in line with the overall schedule of the trial
- Allocation of the EDC system administrator (Admin), data manager, investigator, CRC, clinical research quality inspector, etc.
- Data management design, including database design and logic verification design
- Data processing protocols

- Data quality control
- EDC closure
- Data security and confidentiality measures
- Contingency plans of the EDC system

12. Statistical analysis

The details of statistical analysis are described in the "Statistical Analysis Plan." Before the database is locked, the statisticians discuss the plan with the principal investigators and finalize the plan according to the characteristics of the data. This plan only provides routine statistical requirements.

12.1 Analysis datasets

- **Modified Intention-to-treat (mITT)**

The mITT population is defined as all randomized subjects who received open surgery or laparoscopic surgery. The modified intention-to-treat (mITT) population will serve as the primary population for efficacy analyses in this study.

- **Per-protocol set (PPS):**

The PPS includes patients who meet the inclusion criteria, do not meet the exclusion criteria, and complete the treatment plan. In other words, the participants who meet the trial plan, have good compliance, and complete the items specified in the eCRF are included in the per-protocol (PP) analysis. PP analysis is mainly applied to the primary outcome measure.

- **Safety Set (SS):**

The SS includes the actual data of participants who have received treatment and have records of safety indicators. The missing data of safety indicators cannot be obtained from carry-over from previous records. The dataset includes some patients excluded from the trial, e.g., those whose age exceeds the inclusion criteria. However, it excludes the patients who have used prohibited drugs that interfere with the safety evaluation. The incidence of adverse reactions is determined by using the number of cases in the SS as a denominator.

12.2 Statistical methods

(1) Case enrollment analysis

- The number of patients, the number of enrolled patients, and the number of patients who complete the trial in each center are listed to generate three analysis datasets (FAS, PPS, and SS).
- The patients who drop out and are excluded are listed, along with the reasons.

(2) Demographic data and baseline analysis

- Descriptive statistical, demographic data, and other baseline characteristic values:
  - The number of cases, mean, standard deviation, median, minimum, and maximum, are calculated for continuous variables.
  - The frequency and composition ratios are calculated for the continuous and categorical data.
  - The inferential statistical results (*P* values) are given as descriptive results.

(3) Outcome analysis

Analysis of primary outcome measures

- The DFS is calculated using the Kaplan-Meier method after the last participant has been enrolled for three years. The median DFS time and its 95% confidence interval are compared between the two groups using the log-rank test. Cox regression is used to calculate the hazard ratio and its one-sided 95% confidence interval between the two groups. According to the preset noninferiority criteria, the inferiority of the treatment to be tested compared with the control treatment is determined.

Analysis of secondary outcome measures

- Pathologic outcomes, including the TME quality, negative CRM, and negative DRM, are presented as numbers (percentage) and compared using the Chi-square test.
- The operation time (min) and estimated blood loss (ml) are compared between the groups by the Wilcoxon method.
- The OS is calculated using the Kaplan-Meier method after the last participant has been enrolled for three years. The median OS time and its 95% confidence interval are compared between the two groups using the log-rank test.
- The OS when the last participant is enrolled for five years is determined by using the Kaplan-Meier method. The median OS time and its 95% confidence interval are compared between the two groups using the log-rank test.
- Time to tumor recurrence is determined using the Kaplan-Meier method. The median time of recurrence and its 95% confidence interval are compared between the two groups using the log-rank test.
- The perioperative recovery time is compared between the groups using the Wilcoxon method.
- The length of postoperative hospital stay (d) is compared between the groups using the Wilcoxon method.
- The incidence of intraoperative complications is compared between the groups by the χ^2 test or Fisher's exact probability method.
- After surgery, the incidence of complications and 30-day mortality rate are compared using the χ^2 test or the Fisher's exact tests for probability.

(4) Safety analysis

- The incidences of adverse events and adverse reactions are calculated.

- The frequency and number of adverse events and reactions in different organ systems are recorded, from which the percentage of adverse events/reactions in each system is obtained.
- A detailed list of all adverse event cases is provided.
- A detailed list of all adverse reaction cases is provided.
- The number and percentage of patients whose laboratory tests, ECG, and physical examination results have changed from average to abnormal or show deteriorated abnormalities after the trial are calculated.
- Patients having abnormal laboratory test results, ECG, and physical examination results are listed, along with the clinical explanations.

12.3 Statistical software and general requirements

- SAS 9.4 is used for analysis
- Generally, two-sided tests are used for statistical analysis, and a *P* value < 0.05 indicates that the difference is statistically significant.

13. On-site inspection

All participating hospitals are subject to on-site inspection twice a year by the contract research structure (CRO). The items to be inspected/methods of inspection include:

- Quality of the eCRF records
- Interviewing participants
- All participating hospitals must cooperate with the clinical research quality inspector and provide necessary convenience.

14. Investigators and research structure

14.1 Principal investigator

Professor. Pan Chi (Fujian Medical University Union Hospital)

Address: No. 29, Xinquan Road, Gulou District, Fuzhou, Fujian

Tel: 0591-83357896 to 8060, 13675089677 (mobile phone)

Email: cp3169@163.com

14.2 Participating centers and co-investigators

Center No.	Name	Center
01	Pan Chi	Fujian Medical University Union Hospital
02	Jianping Wang	The Sixth Affiliated Hospital, Sun Yat-sen University

03	Guoxin Li	Nanfang Hospital, Southern Medical University
04	Huizhong Qiu	Peking Union Medical College Hospital, Chinese Academy of Medical Sciences ＆ Peking Union Medical College
05	Xiangqian Su, Aiwen Wu	Peking University Cancer Hospital and Institute
06	Xiaohui Du	The General Hospital of the People's Liberation Army
07	Chun Song	Cancer Hospital of China Medical University, Liaoning Cancer Hospital & Institute
08	Ming Zhong	Renji Hospital, Shanghai Jiao Tong University School of Medicine
09	Kaixiong Tao	Union Hospital, Tongji Medical College, Huazhong University of Science and Technology
10	Kefeng Ding	The Second Affiliated Hospital, School of Medicine, Zhejiang University
11	Ziqiang Wang	West China Hospital, Sichuan University
12	Yong Feng	Shengjing Hospital, China Medical University
13	Chunkang Yang, Weidong Zang, Feng Huang	Fujian Cancer Hospital
14	Kai Ye	The Second Affiliated Hospital, Fujian Medical University
15	Jun You	The First Affiliated Hospital, Xiamen University
16	Yincong Guo	Zhangzhou Affiliated Hospital, Fujian Medical University
17	Dongbo Xu	Longyan Affiliated Hospital, Fujian Medical University
18	Zhizhong Pan	Sun Yat-sen University Cancer Center
19	Xinxiang Li	Fudan University Cancer Center
20	Jianmin Xu	Zhongshan Hospital, Fudan University
21	Weiping Chen	Cancer Hospital of the University of Chinese Academy of Sciences ＆ Zhejiang Cancer Hospital
22	Zhiguo Xiong	Hubei Provincial Cancer Hospital

14.3 Research coordinators

Ying Huang, Associate professor (Fujian Medical University Union Hospital)

Address: No. 29, Xinquan Road, Gulou District, Fuzhou, Fujian

Mobile phone: 13365910923

Email: hy9033sy@sina.com

Weizhong Jiang, Chief physician (Fujian Medical University Union Hospital)

Address: No. 29, Xinquan Road, Gulou District, Fuzhou, Fujian

Mobile phone: 13763828825

Email: jiangwz362100@163.com

14.4 On-site inspector

Beijing Highland Med-Tech Development Co., Ltd. Zhen Li

15. Ethical rules of this study

15.1 Ethical principles

The study plan is designed and implemented following the relevant provisions of the Declaration of Helsinki to protect the rights and interests of all participants.

15.2 Ethical review process

The study plan is chosen by the principal investigator and the sponsor through discussion and is implemented after the ethics committee grants approval before the beginning of the trial.

15.3 Responses to ethical review decisions

After necessary revision, the ethics committee may approve, disapprove the study plan, and terminate/suspend the trial after it has been approved. The research team will revise the plan according to the ethical review comments and implement it according to the final approved plan.

15.4 Registration of this study

Before its beginning, the study was registered at ClinicalTrials.gov with registration number NCT01899547.

15.5 Privacy protection of participants

Investigators must ensure the privacy of participants. Participants' names and patient numbers shall not appear in the documents submitted to the sponsor, in which their trial identity number can only represent the participants. Investigators must keep the enrollment

form meticulously with the participants' names and addresses corresponding to trial identity numbers.

15.6 Modification of the study plan

After the ethics committee approves the study plan, significant modifications can be made during the trial process only after an "Explanation of the Modifications to the Study Plan" is written and signed by the principal investigator of the organization study and is approved by the ethics committee. Nonfundamental modifications can be made after the principal investigator, statistician, and methodologist of the organization in charge of the study discuss and sign the modification form.

16. Adverse events

16.1 Definitions of adverse events

Adverse events refer to any medical phenomena when participants are treated with different surgical procedures or adjuvant chemoradiotherapy regardless of whether the phenomena are causally related to the treatment. This term also covers laboratory test results or other diagnostic procedures considered clinically relevant (e.g., those requiring unscheduled diagnostic procedures or treatment or resulting in withdrawal from the study).

Adverse events may include:

- The symptoms or disease signs or accompanying symptoms deteriorate under treatment
- The treatment efficacy is affected, which may be related to the research procedures
- Any combination of the factors above

Hospitalization before inclusion in the study will not be considered an adverse event. Such criteria are also suitable for patients hospitalized for less than 12 hours; part of the regular treatment or monitoring of the disease to be studied, or the patient is hospitalized not because of deterioration of the disease.

An adverse event is considered to have a severe consequence if it:

- Leads to death
- Is life-threatening
- Requires hospitalization or prolongs hospitalization (the exceptions are listed below)
- Causes permanent or severe disability or incapacity to work
- It is a significant medical event

Death:

If death results from treatment, the cause of death should be considered a serious adverse event. Death resulting from tumor development itself is not included.

An exception to this rule is "sudden death" without a clear cause. In this case, sudden death should be regarded as an adverse event and "fatal" as the cause of being "serious."

Life-threatening:

An adverse event is defined as "serious." "Life threat" refers to adverse events in which a participant is at risk of death due to treatment. It does not refer to adverse events that, if more serious, may lead to the hypothesis of death.

Hospitalization:

Any treatment-related adverse reaction that leads to hospitalization or prolongation of hospitalization is automatically considered serious except for these situations: the hospital stay is shorter than 12 hours, the hospitalization is planned before the beginning of the study (e.g., elective or planned surgery before the beginning of the study), or hospitalization is not related to adverse events (e.g., admission to a social welfare medical institution for short-term care).

During hospitalization, any interventional treatment may meet the criteria of medical importance and, therefore, may be reported as a severe adverse event based on clinical judgment. In addition, when local regulatory authorities explicitly require stricter definitions, local regulations are judged by precedent.

Disability:

Disability refers to the substantial destruction of a person's ability to live a normal life due to treatment.

Significant medical events:

Any treatment-related adverse event can be considered severe because it may endanger the participant and require interventions to prevent another difficult situation from occurring. A significant medical event may be indicative of a seriously ill state. Therefore, it is essential to ensure that special attention is paid to reporting significant medical events, which might be more critical than the reports on other issues.

16.2 Expected adverse events

Operation-related adverse events:

The complications specific to laparoscopic surgery include subcutaneous emphysema, hypercapnia, vascular and gastrointestinal injuries associated with a puncture, and gas embolism.

The common complications of laparoscopic surgery and open surgery mainly include:

1) Presacral hemorrhage
2) Anastomotic leakage
3) Anastomotic bleeding
4) Active intra-abdominal bleeding
5) Intestinal adhesion and intestinal obstruction
6) Internal hernia
7) Injuries to the surrounding organs, including the ureter and duodenum
8) Intestinal fistula and lymphatic leakage
9) Urination disorder and sexual dysfunction
10) Difficulty in bowel movement or frequent bowel movements
11) Complications of colostomy
12) Incision complications (infection, effusion, dehiscence, poor healing, etc.)
13) Pneumonia, urinary tract infection, renal failure, liver failure, cardiovascular and cerebrovascular events (including thrombosis, embolism, etc.)
14) Others.

Adverse events caused by deterioration of the primary disease:

Adverse events related to various forms of deterioration of the primary disease are recorded according to the "Short Name" in Common Terminology Criteria for Adverse Events (CTCAE) v4.0. They include:

1) Adverse events caused by primary lesions and peritoneal disseminated lesions:

Gastrointestinal tract: loss of appetite, constipation, dehydration, abdominal fullness, heartburn, nausea, gastrointestinal obstruction [stomach, duodenum, ileum, colon, and small intestine (not subdivided)], gastrointestinal perforation (stomach, duodenum, jejunum, ileum, and colon), gastrointestinal stenosis (stomach, duodenum, jejunum, ileum, and colon), vomiting, hyponatremia, gastrointestinal bleeding (stomach, duodenum, jejunum, ileum, and colon)

2) Adverse events caused by liver metastasis:

Abnormal metabolic/clinical examination results: aspartate aminotransferase (AST), alanine aminotransferase (ALT), bilirubin, and alkaline phosphatase

3) Adverse events caused by lung metastasis:

Lung/upper respiratory tract: atelectasis, dyspnea, hypoxemia, and airway occlusion (bronchi)

4) Adverse events caused by other metastatic lesions:

Pain: pain (metastasis sites) and hypercalcemia

5) Adverse events related to the deterioration of general condition:

- General conditions: fatigue, weight loss, and cachexia
- Blood/bone marrow: abnormal hemoglobin and platelet levels
- Cardiovascular system: hypotension
- Lymphatic system: edema in the head and neck, limbs, body trunk/genitals, and viscera
- Metabolism/clinical test results: hypoproteinemia, AST, ALT, acidosis, creatinine, hyperglycemia, hypoglycemia, hypernatremia, hyponatremia, hyperkalemia, hypokalemia, and other electrolyte disorders
- Lung/ upper respiratory tract: pleural effusion (nonmalignant), dyspnea, hypoxemia, and lung infection
- Genitourinary organs: cystitis, renal failure, and oliguria/anuria

16.3 Grading of adverse events

In this study, adverse events are assessed and graded according to the Chinese version of CTCAE v4.02 and the Accordion Severity Grading System. Adverse events are graded based on their severity. CTCAE provides specific clinical descriptions of the severity of each adverse event (grades 1 to 5) according to the following general rules:

Grade 1: mild; asymptomatic or minor; only clinically or diagnostically determined; no treatment required

Grade 2: moderate; requiring minor, local or noninvasive treatment; restriction of instrumental activities of daily living (ADL) *corresponding to age

Grade 3: severe or medically significant, but not immediately life-threatening; leading to hospitalization or prolonged hospitalization; leading to disability; restriction of personal ADL

Grade 4: life-threatening; emergency treatment required

Grade 5: adverse event-related deaths

ADL: * Instrumental ADL refers to cooking, shopping for clothes, using the telephone, managing finances, etc.

** Personal ADL refers to bathing, dressing/undressing, eating, washing, taking medication, etc., as well as not being bedridden.

16.4 Recording of adverse events

All adverse events must be recorded on the adverse event report form attached to the eCRF. For each adverse event, the investigator must assess and record its severity, duration, relationship to the surgery or the studied drug, measures that have been taken, and the outcome of the event.

16.5 Reporting of adverse events

- For any serious adverse event or unexpected adverse event, the investigator must report the event to the Research Committee/PI (Prof. Pan Chi), the ethics committee responsible for dealing with serious adverse events, and the local personnel in charge of medical safety management within 24 hours after knowing of the adverse event, regardless whether the event is related to the treatment.
- The outcomes (regression, death, etc.) of serious adverse events that have been reported will be followed up and recorded. According to the national regulations, the study's organization reports the data to the relevant authorities. In any case, all personnel of the study must comply with local legal provisions and requirements.

17. Confirmation of raw data

17.1 Raw data

The following items 1-4 are the relevant raw data for this study, and items 5 and 6 are recorded directly in the eCRF and used as raw data. In addition, after approval by the investigators, a raw data list is created for each center according to the clinical research quality assurance plan, and a copy is submitted to the sponsoring organization.

1) Medical records: case registration forms, informed consent forms, and the symptoms, physical examination results, and treatments of patients
2) Examination reports (electrocardiogram, ultrasound, imaging, pathology): biopsy and pathological examination of gross specimens, endorectal ultrasound, MRI, CT or X-ray, etc.
3) Data reported by automatic testing devices: complete blood count, biochemical parameters, tumor markers, and other laboratory test results
4) Imaging data of surgery and specimens: unedited laparoscopic surgery videos, intraoperative photos, postoperative specimens, etc.
5) Information on the occurrence, severity, course, and outcomes of adverse events, as well as their causal relationships with the study and the corresponding explanations
6) An explanation for discontinuation and outcomes of the study

17.2 Quality assurance of raw data

The quality inspector compares the eCRF with raw data to confirm the items below. If there is any discrepancy with the raw data, the PI will be asked to give a written explanation.

1) The data required by the study plan are correctly recorded in the eCRF and are consistent with the raw data.
2) Any changes in the treatment protocols or treatment method should be fully recorded in the eCRF.
3) Adverse events are recorded in the eCRF according to the study plan.
4) The observations and examinations that cannot be performed because the participant misses the follow-up visits are recorded in the eCRF.

5) All withdrawals and dropouts after enrollment are recorded in the eCRF with an explanation of the reasons.

18. Quality management and control

18.1 Clinical research quality management

The sponsor shall implement the relevant standard operation procedure (SOP) and the quality assurance plan of this study following clinical research requirements. The measures below are taken for quality management.

1) The principal investigators are called together for a meeting and given detailed explanations of the study plan.
2) All participating centers are regularly inspected.
3) Instructions for filling out or revising the eCRF are formulated.
4) The records on the eCRF are inspected and verified.

18.2 Quality management of participating centers, and surgeon eligibility

1) Hospitals with more than 30 laparoscopic TME surgeries per year were qualified to enroll in this study.

2) The leading organization, participating hospitals, and investigators must fulfill their duties and strictly follow the study plan. A detailed SOP is formulated, and a series of measures are taken, including auditing, inspection, and supervision, to ensure that all relevant personnel in all aspects are fully responsible and cooperative and strictly follow the requirements of good clinical practice and SOP and ensure all data and records authentic and reliable.

3) The principal investigators of the study are the leading researchers of each center.

4) After treatment, diagnosis confirmation and outcome assessment are completed by at least one investigator with the title of attending physician or above, and the arrangement should be kept relatively consistent.

5) The investigators fill out the eCRF according to the requirements. The form is carefully filled out item by item at the first visit and after the patient's treatment. The principal investigators should conduct regular inspections. After the completion of the trial, personnel at all levels sign the forms after final confirmation.

6) The leading organization should keep in touch with each research center, and the research centers should communicate with each other regularly. The problems encountered in the early stage of the study should be brought up and discussed promptly. If the study plan needs to be modified, the chief research expert from the leading organization shall organize a meeting to discuss and revise the study plan.

The revised plan needs to be re-reviewed and approved by the ethics committee before implementation.

7) An academic credentialing committee accredited surgeons with experience of more than 100 laparoscopic TME surgeries after a blinded review of at least two unedited videos of laparoscopic TME surgeries.

18.3 Surgical quality management

1) If the anal sphincter is preserved or partially preserved, the distance (cm) between the anastomosis and the anal verge must be recorded after the operation.

2) Surgical imaging data: The unedited video of each laparoscopic surgery is saved and backed up to the sponsoring organization for unified storage within 12 months after surgery. The Academic Credentialing Committee conducts a review and monitoring of the surgical quality of laparoscopic surgery per 6 months.

3) Intraoperative photographs: The images showing the dissection of the No. 253 lymph nodes are routinely collected during surgery using a digital camera with 8 million or more pixels or a laparoscopic surgery video-recording system (**Fig. 1**).

4) Postoperative specimen photographs:

- After the surgical specimen is taken out of the body, the integrity of the mesorectum is checked and photographed by a digital camera with 8 million pixels (**Fig. 2**).

- Cut the intestinal wall along the anterior wall of the rectum and avoid the lesion. The mucous membrane of the cut specimen was placed upward on a plate or similar object, which was stretched and fixed in a nearly physiological state, and photographed (placed with a ruler), measured, and recorded (**Fig. 3**).

5) Data recorded: the distance between the upper margin of the tumor and the opening-side margin of the resected rectum, the distance between the lower margin of the tumor and the anus-side margin of the resected rectum (or the resected skin margin), the size of the tumor, and the ratio of the maximum diameter of the tumor to the transverse diameter of the intestinal canal.

6) **Numbering of surgical and specimen imaging data:** The video of the laparoscopic surgery is given the same number as **the patient's randomization number**. The intraoperative photos showing the dissection of the No. 253 lymph nodes and postoperative specimen photos are numbered as the participant's randomization number plus Fig. 1, Fig. 2, or Fig. 3. For example, the photos of patient 001 are numbered 001 Fig. 1, 001 Fig. 2, and 001 Fig. 3 accordingly.

7) The intraoperative photos showing the dissection of the No. 253 lymph nodes and postoperative specimen photos are uploaded to the EDC system within one week after surgery.

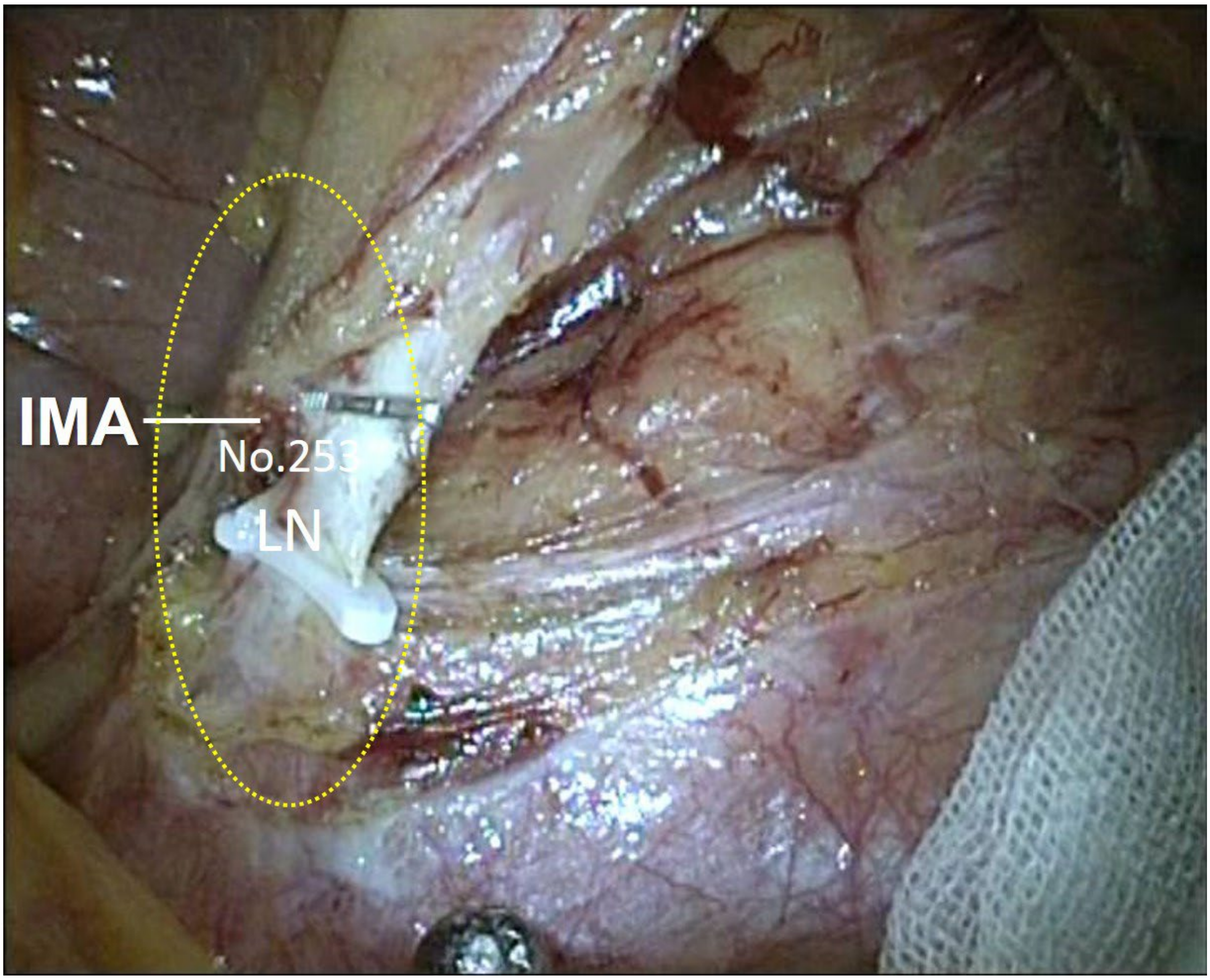

Fig. 1 Dissection of the No. 253 lymph nodes

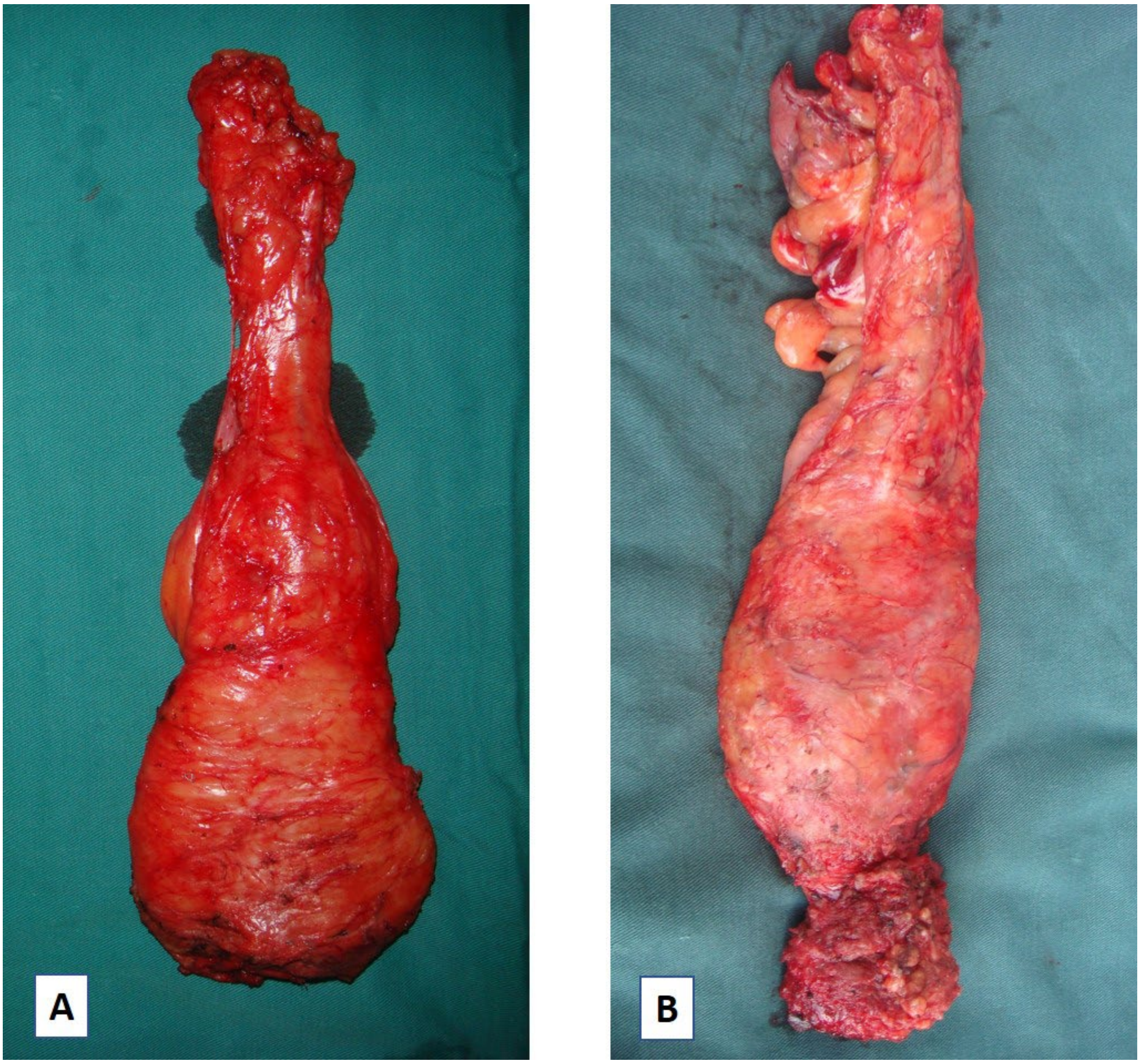

Fig. 2 Macroscopic assessment of mesorectum. (A) low anterior resection (LAR) and (B) abdominoperineal resection (APR).

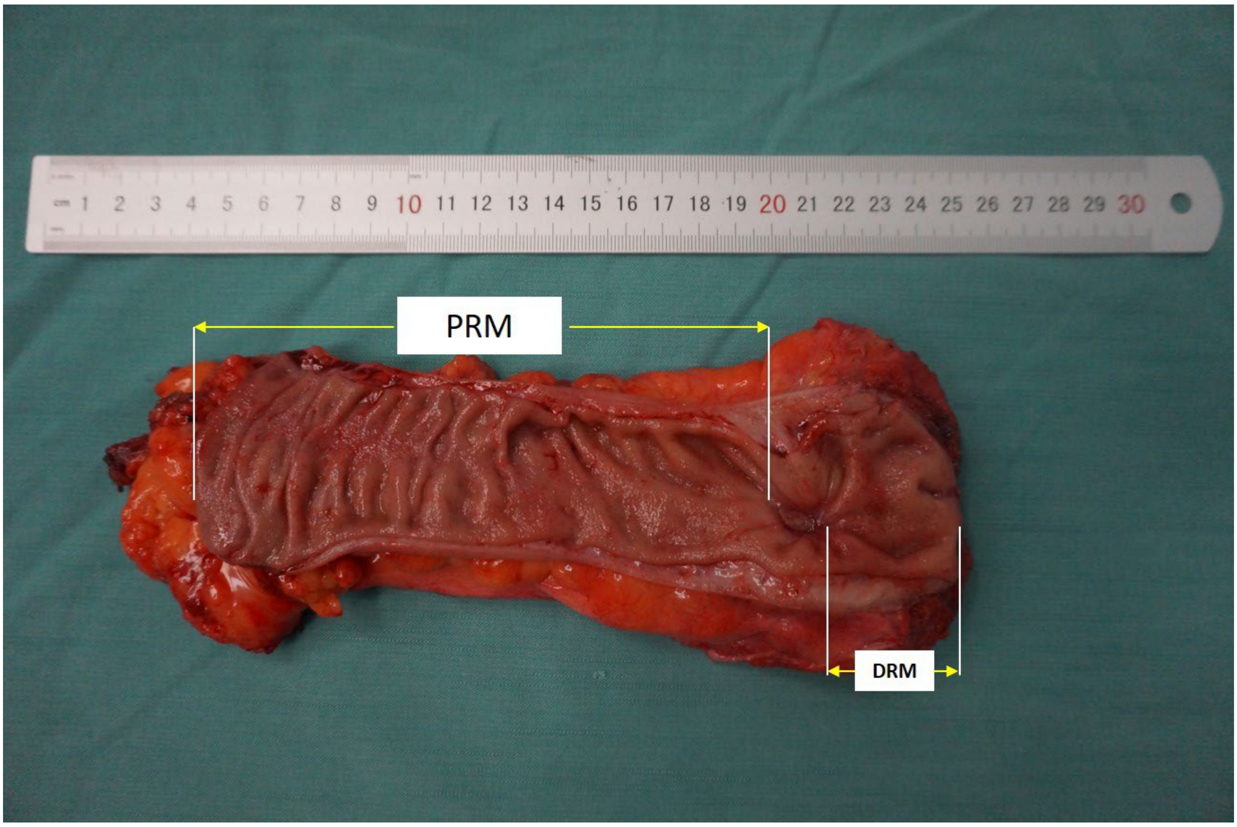

Fig. 3 Distances of proximal resection margin (PRM), distal resection margin (DRM), and characteristics of the tumor lesion

19. Publication policy

This study aims to publish the findings in research papers in professional medical journals and abstracts/introductions at professional medical conferences. Any individual investigator must obtain permission from the leading organization to publish papers based on the findings of this study or based on the participants recruited by himself/herself.

In the publications and research reports, the names of investigators will be listed according to the number of patients admitted/treated by the investigator and who have complete and valid research data.

20. Changes to the Study protocol

The original and final versions of the study protocol and the changes between each version.

Version	Date	Brief Description of Change
1.0	August14,2013	-
2.0	September 20, 2013	Added eight participating centers, as follows:<br>● The Sixth Affiliated Hospital, Sun Yat-sen University<br>● Peking Union Medical College Hospital, Chinese Academy of Medical Sciences ＆ Peking Union Medical College<br>● Peking University Cancer Hospital and Institute<br>● Cancer Hospital of China Medical University, Liaoning Cancer Hospital & Institute<br>● Union Hospital, Tongji Medical College, Huazhong University of Science and Technology<br>● The Second Affiliated Hospital, School of Medicine, Zhejiang University<br>● West China Hospital, Sichuan University<br>● Shengjing Hospital, China Medical University
3.0	May 18, 2015	Added five participating centers, as follows:<br>● Fudan University Cancer Center<br>● Zhongshan Hospital, Fudan University<br>● Cancer Hospital of the University of Chinese Academy of Sciences ＆ Zhejiang Cancer Hospital<br>● Hubei Provincial Cancer Hospital<br>● Sun Yat-sen University Cancer Center

21. References

1. World Health Organization. WHO Mortality Database. Available at: http://www. Dep. iarc.fr/. Accessed January 15, 2011.
2. Heald RJ, Husband EM, Ryall RD. The mesorectum in rectal cancer surgery--the clue to pelvic recurrence?. Br J Surg. 1982;69(10):613-616. doi:10.1002/bjs. 1800691019.
3. Wang JP. Diagnosis and treatment of middle-low rectal cancer in the status quo and outlook. Chinese Journal of Practical Surgery, 2009, 29(4):287-290.
4. Guillou PJ, Quirke P, Thorpe H, et al. Short-term endpoints of conventional versus laparoscopic-assisted surgery in patients with colorectal cancer (MRC CLASICC trial): multicentre, randomised controlled trial. Lancet. 2005;365(9472):1718-1726. doi:10.1016/S0140-6736(05)66545-2.
5. Jayne DG, Brown JM, Thorpe H, et al. Bladder and sexual function following resection for rectal cancer in a randomized clinical trial of laparoscopic versus open technique. Br J Surg. 2005;92(9):1124-1132. doi:10.1002/bjs.4989.
6. Jayne DG, Guillou PJ, Thorpe H, et al. Randomized trial of laparoscopic-assisted resection of colorectal carcinoma: 3-year results of the UK MRC CLASICC Trial Group. J Clin Oncol. 2007;25(21):3061-3068. doi:10.1200/JCO.2006.09.7758.
7. Jayne DG, Thorpe HC, Copeland J, et al. Five-year follow-up of the Medical Research Council CLASICC trial of laparoscopically assisted versus open surgery for colorectal cancer. Br J Surg. 2010;97(11):1638-1645. doi:10.1002/bjs.7160.
8. Taylor GW, Jayne DG, Brown SR, et al. Adhesions and incisional hernias following laparoscopic versus open surgery for colorectal cancer in the CLASICC trial. Br J Surg. 2010;97(1):70-78. doi:10.1002/bjs.6742.
9. Ng SS, Leung KL, Lee JF, et al. Laparoscopic-assisted versus open abdominoperineal resection for low rectal cancer: a prospective randomized trial [published correction appears in Ann Surg Oncol. 2009 Jan;16(1):229]. Ann Surg Oncol. 2008;15(9):2418-2425. doi:10.1245/s10434-008-9895-0.
10. Miyajima N, Fukunaga M, Hasegawa H, et al. Results of a multicenter study of 1,057 cases of rectal cancer treated by laparoscopic surgery. Surg Endosc. 2009;23(1):113-118. doi:10.1007/s00464-008-0078-7.
11. Gao F, Cao YF, Chen LS. Meta-analysis of short-term outcomes after laparoscopic resection for rectal cancer. Int J Colorectal Dis. 2006;21(7):652-656. doi:10.1007/ s00384-005-0079-0.
12. Xu ZR, Chi P. Comparison of the incidence of postoperative complications following laparoscopic and open colorectal cancer resection [J]. Chinese Journal of Gastrointestinal Surgery, 2012, 15(8):810-813.
13. Aziz O, Constantinides V, Tekkis PP, et al. Laparoscopic versus open surgery for rectal cancer: a meta-analysis. Ann Surg Oncol. 2006;13(3):413-424. doi:10.1245/ASO. 2006. 05.045.
14. Laurent C, Leblanc F, Bretagnol F, et al. Long-term wound advantages of the laparoscopic approach in rectal cancer. Br J Surg. 2008;95(7):903-908. doi:10.1002/bjs.6134.

15. Morino M, Allaix ME, Giraudo G, et al. Laparoscopic versus open surgery for extraperitoneal rectal cancer: a prospective comparative study. Surg Endosc. 2005;19(11):1460-1467. doi:10.1007/s00464-004-2001-1.
16. Lezoche E, Guerrieri M, De Sanctis A, et al. Long-term results of laparoscopic versus open colorectal resections for cancer in 235 patients with a minimum follow-up of 5 years. Surg Endosc. 2006;20(4):546-553. doi:10.1007/s00464-005-0338-8.
17. Li S, Chi P, Lin H, et al. Long-term outcomes of laparoscopic surgery versus open resection for middle and lower rectal cancer: an NTCLES study. Surg Endosc. 2011;25(10):3175-3182. doi:10.1007/s00464-011-1683-4.
18. Breukink SO, Pierie JP, Grond AJ, et al. Laparoscopic versus open total mesorectal excision: a case-control study. Int J Colorectal Dis. 2005;20(5):428-433. doi:10.1007/s00384-004-0715-0.
19. Bretagnol F, Lelong B, Laurent C, et al. The oncological safety of laparoscopic total mesorectal excision with sphincter preservation for rectal carcinoma. Surg Endosc. 2005;19(7):892-896. doi:10.1007/s00464-004-2228-x.
20. Anderson C, Uman G, Pigazzi A. Oncologic outcomes of laparoscopic surgery for rectal cancer: a systematic review and meta-analysis of the literature. Eur J Surg Oncol. 2008;34(10):1135-1142. doi:10.1016/j.ejso.2007.11.015.
21. Liang Y, Li G, Chen P, et al. Laparoscopic versus open colorectal resection for cancer: a meta-analysis of results of randomized controlled trials on recurrence. Eur J Surg Oncol. 2008;34(11):1217-1224. doi:10.1016/j.ejso.2007.11.004.
22. Konishi T, Watanabe T, Kishimoto J, et al. Elective colon and rectal surgery differ in risk factors for wound infection: results of prospective surveillance. Ann Surg. 2006;244(5):758-763. doi:10.1097/01.sla.0000219017.78611.49.
23. Lin HY, Chi P. Comparison of urinary function and sexual function between laparoscopic and open radical resection for rectal cancer [J]. Chinese Journal of Gastrointestinal Surgery, 2011, 14(4):289-290.
24. Zheng MH, Hu YY, Lu AG, et al. Clinical comparison of laparoscopic and open total mesorectal excision for lower rectal cancer. Chinese Journal of Gastrointestinal Surgery, 2004, 7(3): 177-180.
25. Zheng MH. Thoughts on the current situation of minimally invasive gastrointestinal surgery. Chinese Journal of Digestive Surgery, 2012,11(1): 22-24.
26. Zheng MH. Whether laparoscopic operation will be the gold standard for the surgical treatment of gastrointestinal neoplasms. Chinese Journal of Digestive Surgery, 2012,11(3):161-164.
27. American College of Surgeons. Laparoscopic-Assisted Resection or Open Resection in Treating Patients With Stage IIA, Stage IIIA, or Stage IIIB Rectal Cancer. http://clinicaltrials.gov/ct2/show/NCT00726622. It was accessed on September 2, 2011.
28. COLOR II: Laparoscopic Versus Open Rectal Cancer Removal. http://clinicaltrials.gov/ ct2/show/ NCT00297791. Accessed September 2, 2011.
29. Kang SB, Park JW, Jeong SY, et al. Open versus laparoscopic surgery for mid or low rectal cancer after neoadjuvant chemoradiotherapy (COREAN trial): short-term

outcomes of an open-label randomised controlled trial. Lancet Oncol, 2010,11:637-645.
30. National Comprehensive Cancer Network. NCCN Clinical Practice Guidelines in Oncology, Rectal Cancer, Version 4.2013.https//www.nccn.org/professionals /physician_gls/pdf/rectal.pdf [accessed 1 August 2013].
31. Dindo D, Demartines N, Clavien PA. Classification of surgical complications: a new proposal with evaluation in a cohort of 6336 patients and results of a survey. Ann Surg. 2004;240(2):205-213. doi:10.1097/01.sla.0000133083.54934. ae.
32. Nagtegaal ID, van de Velde CJ, van der Worp E, et al. Macroscopic evaluation of rectal cancer resection specimen: clinical significance of the pathologist in quality control. J Clin Oncol. 2002;20(7):1729-1734. doi:10.1200/JCO.2002.07.010.
33. Watanabe T, Itabashi M, Shimada Y, et al. Japanese Society for Cancer of the Colon and Rectum (JSCCR) guidelines 2010 for the treatment of colorectal cancer. Int J Clin Oncol. 2012;17(1):1-29. doi:10.1007/s10147-011-0315-2.
34. Hofheinz RD, Wenz F, Post S, et al. Chemoradiotherapy with capecitabine versus fluorouracil for locally advanced rectal cancer: a randomised, multicentre, non-inferiority, phase 3 trial. Lancet Oncol. 2012;13(6):579-588. doi:10.1016/ S1470-2045(12)70116-X.
35. Oken MM, Creech RH, Tormey DC, et al. Toxicity and response criteria of the Eastern Cooperative Oncology Group. Am J Clin Oncol. 1982;5(6):649-655.
36. Dripps RD, Lamont A, Eckenhoff JE. The role of anesthesia in surgical mortality. JAMA. 1961;178:261-266. doi:10.1001/jama.1961.03040420001001.
37. Dworak O, Keilholz L, Hoffmann A. Pathological features of rectal cancer after preoperative radiochemotherapy. Int J Colorectal Dis. 1997;12(1):19-23. doi:10.1007/s003840050072.
38. Aaronson NK, Ahmedzai S, Bergman B, et al. The European Organization for Research and Treatment of Cancer QLQ-C30: a quality-of-life instrument for use in international clinical trials in oncology. J Natl Cancer Inst. 1993;85(5):365-376. doi:10.1093/jnci/85.5.365.
39. Gujral S, Conroy T, Fleissner C, et al. Assessing quality of life in patients with colorectal cancer: an update of the EORTC quality of life questionnaire. Eur J Cancer. 2007;43(10):1564-1573. doi:10.1016/j.ejca.2007.04.005.
40. Barry MJ, Fowler FJ Jr, O'Leary MP, et al. The American Urological Association symptom index for benign prostatic hyperplasia. The Measurement Committee of the American Urological Association. J Urol. 1992;148(5):1549-1564. doi:10.1016/s0022-5347(17)36966-5.
41. Rosen RC, Riley A, Wagner G, Osterloh IH, Kirkpatrick J, Mishra A. The international index of erectile function (IIEF): a multidimensional scale for assessment of erectile dysfunction. Urology. 1997 Jun;49(6):822-30. doi: 10.1016/s0090-4295(97)00238-0. PMID: 9187685.
42. Rosen R, Brown C, Heiman J, et al. The Female Sexual Function Index (FSFI): a multidimensional self-report instrument for the assessment of female sexual function. J Sex Marital Ther. 2000;26(2):191-208. doi:10.1080/009262300278597.

43. Jorge JM, Wexner SD. Etiology and management of fecal incontinence. Dis Colon Rectum. 1993;36(1):77-97. doi:10.1007/BF02050307.

Appendix 1: Trial flow

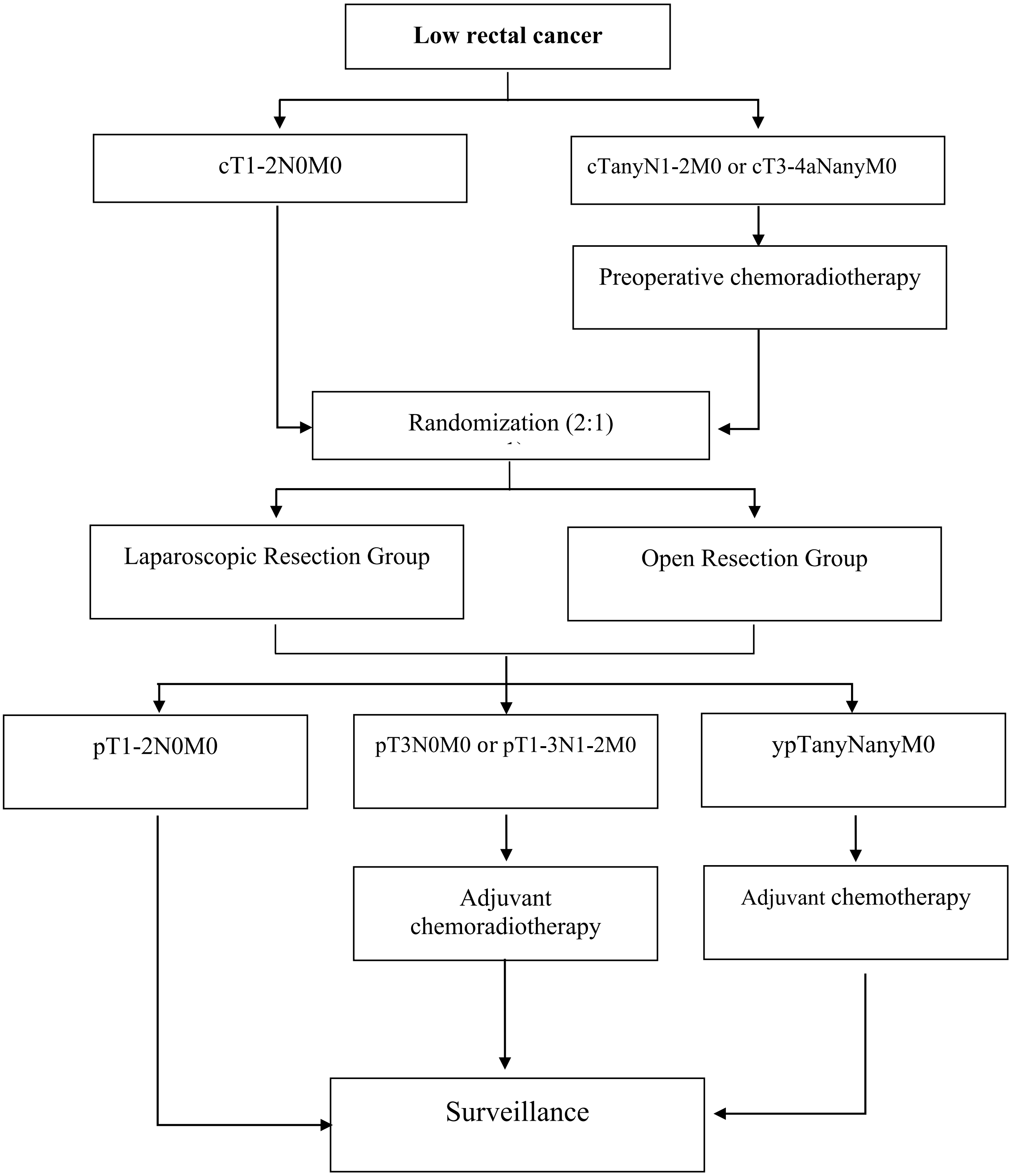

Appendix 2: Assessment schedule and process

Assessment schedule for the screening/treatment period

	Screening: 14 days through 1 day before neoadjuvant radiotherapy	Screening: 5 days before randomization	Adjuvant therapy	
			Adjuvant chemotherapy	Adjuvant radiotherapy
**Treatment plan**			7-1 days before each chemotherapy session	7-1 days before radiotherapy
Informed consent		×		
Demographic data	×	×		
Medical history (a)	×	×		
General physical examination	×	×	×	×
Height, weight, body surface area (BSA)	×	×	×	×
ECOG PS score	×	×	×	×
Concomitant diseases & Treatment	×	×		
Complete blood count	×	×	×	×
Blood biochemistry	×	×	×	×
Coagulation test		×		
Pregnancy test (if applicable)		×		
Tumor markers (b)	×	×		
ECG	×	×	×	×
Echocardiography (c)		×		
Pulmonary function (c)		×		
Chest X-ray or CT	×	×		
Contrast-enhanced MRI of the pelvis (d)	×	×		
Contrast-enhanced MRI of the liver (d)	×	×		
Endorectal ultrasound (d)	×	×		
EORTC QLQ-C30v.3 score		×		
EORTC QLQ-CR29 score		×		
The residual urine volume in the bladder		×		
I-PSS score		×		
IIEF-5 score		×		
FSFI score		×		
Adverse events and treatment			×	×

Note:

(a) Participant number, case number, date of informed consent, sex, age, clinical stage, tissue type, medical history, complications, and clinical diagnosis.

(b) Tumor markers (CEA and CA199) of patients receiving neoadjuvant chemoradiotherapy: The CEA and CA199 levels are measured within 14 days before treatment, before the third session of preoperative chemotherapy, and within 14 days before surgery. They are also measured once every three months before and during adjuvant chemotherapy.

(c) Echocardiography and pulmonary function examination should be performed if necessary by the investigator.

(d) Imaging examination of patients undergoing neoadjuvant chemoradiotherapy: The imaging examination should be given within 14 days before treatment and 14 days before surgery. It is given once every three months during adjuvant therapy. In the hospitals where endorectal ultrasound is available, endorectal ultrasound should be jointly used for clinical staging.

Assessment schedule for the treatment/follow-up period

	Postoperative time (f)									
	**Day**	**Month**								
	**5**	**3**	**6**	**9**	**12**	**15**	**18**	**21**	**24**	**30 – 60 (g)**
Tumor markers		×	×	×	×	×	×	×	×	×
Chest X-ray (a)		×	×	×	×	×	×	×	×	×
Abdominal/pelvic ultrasound (a)		×	×	×	×	×	×	×	×	×
Contrast-enhanced MRI or CT of the liver or pelvis (b)					×				×	×
Fiberoptic colonoscopy (c)		×			×					×
EORTC QLQ-C30 v.3 score		×	×		×					
EORTC QLQ-CR29 score		×	×		×					
The residual urine volume in the bladder	×	×	×		×					
I-PSS score		×	×		×					
IIEF-5 score		×	×		×					
FSFI score		×	×		×					
Assessment of anorectal function					×					
Survival (d)		×	×	×	×	×	×	×	×	×
Other cancer treatments (e)	**Record when it occurs**									

Note:

(a) If the patient shows signs of tumor recurrence (e.g., clinical symptoms), additional tumor assessment is required. All assessments to confirm the recurrence/new onset of tumors should be recorded in the eCRF, including tumor assessments not included in the study plan.

(b) MRI or CT: The use of oral or intravenous contrast agents should be kept as consistent as possible. The same investigator or radiologist assessed each patient's scan results as consistently as possible during the study.

(c) Patients whose preoperative fiberoptic colonoscopy did not pass the lesion should undergo the fiberoptic colonoscopy three months after surgery.

(d) Tumor recurrence and the survival of patients are followed up until death. The last known survival date or data from the last two years were used for the analysis of patients who survived.

(e) **Other cytotoxic agents, drugs to be studied, and active or passive immunotherapy for rectal cancer are not allowed during the treatment or follow-up period without tumor recurrence.**

(f) The examinations can be done within ± four weeks of the planned date, but the 6-month tumor assessment must be done after the treatment of all studied drugs is completed.

(g) The examinations are done once every six months. Fiberoptic colonoscopy is done once a year and again within one year if any abnormality is detected. Fiberoptic colonoscopy is done again within three years if no polyps are found and then once every five years. It is recommended that colorectal adenomas found in follow-up examinations be surgically removed.

Appendix 3: ECOG Performance Status

GRADE	ECOG PERFORMANCE STATUS
0	Fully active, able to carry on all pre-disease performance without restriction
1	Restricted in physically strenuous activity but ambulatory and able to carry out work of a light or sedentary nature, e.g., light housework, office work
2	Ambulatory and capable of all self-care but unable to carry out any work activities; up and about more than 50% of waking hours
3	Capable of only limited self-care; confined to bed or chair more than 50% of waking hours
4	Completely disabled; cannot carry on any self-care; totally confined to bed or chair
5	Dead

Appendix 4: The ASA classification of physical status

Class	Definition
1	A normally healthy patient
2	A patient with mild systemic disease
3	A patient with a severe systematic disease that is not incapacitating
4	A patient with incapacitating severe disease that is a constant threat to life
5	A moribund patient who is not expected to survive for 24 h with or without an operation

The addition of 'E' denotes emergency surgery

Appendix 5: Tumor regression grading

The histological regression grade of the primary tumor (TRG) and the regional lymph nodes (LRG) were assessed according to the proposals of Dworak et al.

Grade 0: no regression;
Grade 1: dominant tumor mass with obvious fibrosis and/or vasculopathy;
Grade 2: dominantly fibrotic changes with few tumor cells or groups (easy to find);
Grade 3: very few (difficult to find microscopically) tumor cells in fibrotic tissue with or without mucous substance;
Grade 4: no tumor cells, only fibrotic mass (total regression or response)

Appendix 6: Quality of life (QoL)

6.1 EORTC QLQ-C30 (Version 3.0)

We are interested in some things about you and your health. Please answer all of the questions yourself by circling the number that best applies to you. There are no "right" or "wrong" answers. The information that you provide will remain strictly confidential.

	**Not at All**	**A Little**	**Quite a Bit**	**Very Much**
1. Do you have any trouble doing strenuous activities, like carrying a heavy shopping bag or a suitcase?	1	2	3	4
2. Do you have any trouble taking a long walk?	1	2	3	4
3. Do you have any trouble taking a short walk outside of the house?	1	2	3	4
4. Do you need to stay in bed or a chair during the day?	1	2	3	4
5. Do you need help with eating, dressing, washing yourself or using the toilet?	1	2	3	4

**During the past week:**	**Not at All**	**A Little**	**Quite a Bit**	**Very Much**
6. Were you limited in doing either your work or other daily activities?	1	2	3	4
7. Were you limited in pursuing your hobbies or other leisure time activities?	1	2	3	4
8. Were you short of breath?	1	2	3	4
9. Have you had pain?	1	2	3	4
10. Did you need to rest?	1	2	3	4
11. Have you had trouble sleeping?	1	2	3	4
12. Have you felt weak?	1	2	3	4
13. Have you lacked appetite?	1	2	3	4
14. Have you felt nauseated?	1	2	3	4
15. Have you vomited?	1	2	3	4
16. Have you been constipated?	1	2	3	4

**During the past week:**	**Not at All**	**A Little**	**Quite a Bit**	**Very Much**

17. Have you had diarrhea?	1	2	3	4
18. Were you tired?	1	2	3	4
19. Did pain interfere with your daily activities?	1	2	3	4
20. Have you had difficulty in concentrating on things, like reading a newspaper or watching television?	1	2	3	4
21. Did you feel tense?	1	2	3	4
22. Did you worry?	1	2	3	4
23. Did you feel irritable?	1	2	3	4
24. Did you feel depressed?	1	2	3	4
25. Have you had difficulty remembering things?	1	2	3	4
26. Has your physical condition or medical treatment interfered with your family life?	1	2	3	4
27. Has your physical condition or medical treatment interfered with your social activities?	1	2	3	4
28. Has your physical condition or medical treatment caused you financial difficulties?	1	2	3	4

For the following questions, please circle the number between 1 and 7 that best applies to you

29. How would you rate your overall health during the past week?

1	2	3	4	5	6	7
Very poor						Excellent

30. How would you rate your overall Quality of life(QoL) during the past week?

1	2	3	4	5	6	7
Very poor						Excellent

6.2 EORTC QLQ-CR29

Patients sometimes report that they have the following symptoms or problems. Please indicate the extent to which you have experienced these symptoms or problems during the past week. Please answer by circling the number that best applies to you.

**During the past week:**	**Not at All**	**A Little**	**Quite a Bit**	**Very Much**
31. Did you urinate frequently during the day?	1	2	3	4
32. Did you urinate frequently during the night?	1	2	3	4

33. Have you had any unintentional release (leakage) of urine?	1	2	3	4
34. Did you have pain when you urinated?	1	2	3	4
35. Did you have abdominal pain?	1	2	3	4
36. Did you have pain in your buttocks/anal area/rectum?	1	2	3	4
37. Did you have a bloated feeling in your abdomen?	1	2	3	4
38. Have you had blood in your stools?	1	2	3	4
39. Have you had mucus in your stools?	1	2	3	4
40. Did you have a dry mouth?	1	2	3	4
41. Have you lost hair as a result of your treatment?	1	2	3	4
42. Have you had problems with your sense of taste?	1	2	3	4

**During the past week:**	**Not at All**	**A Little**	**Quite a Bit**	**Very Much**
43. Were you worried about your health in the future?	1	2	3	4
44. Have you worried about your weight?	1	2	3	4
45. Have you felt physically less attractive as a result of your disease or treatment?	1	2	3	4
46. Have you been feeling less feminine/masculine as a result of your disease or treatment?	1	2	3	4
47. Have you been dissatisfied with your body?	1	2	3	4
48. Do you have a stoma bag (colostomy/ileostomy)? (please circle the correct answer)	Yes		No	

During the past 4 weeks:

Not at A Quite
Very AllLittle a Bit
Much

Answer these questions ONLY IF YOU HAVE A STOMA BAG. If not, please continue below:

49. Have you had an unintentional release of gas/flatulence from your stoma bag?	1	2	3	4
50. Have you had leakage of stools from your stoma bag?	1	2	3	4
51. Have you had sore skin around your stoma?	1	2	3	4
52. Did frequent bag changes occur during the day?	1	2	3	4

53. Did frequent bag changes occur during the night?	1	2	3	4
54. Did you feel embarrassed because of your stoma?	1	2	3	4
55. Did you have problems caring for your stoma?	1	2	3	4

<u>Answer these questions ONLY IF YOU DO NOT HAVE A STOMA BAG:</u>

49. Have you had unintentional release of gas/flatulence from your back passage?	1	2	3	4
50. Have you had leakage of stools from your back	1	2	3	4
51. Have you had sore skin around your anal	1	2	3	4
52. Did frequent bowel movements occur during the	1	2	3	4
53. Did frequent bowel movements occur during the	1	2	3	4
54. Did you feel embarrassed because of your bowel	1	2	3	4

During the past 4 weeks:

	Not at	A Very Much	All Quite Little	a Bit
- **<u>For men only:</u>**				
56. To what extent were you interested in sex?	1	2	3	4
57. Did you have difficulty getting or maintaining an	1	2	3	4
- **<u>For women only:</u>**				
58. To what extent were you interested in sex?	1	2	3	4
59. Did you have pain or discomfort during	1	2	3	4

Appendix 7: The International Prostate Symptom Score

In the past month:	Not at All	Less than 1 in 5 Times	Less than Half the Time	About Half the Time	More than Half the Time	Almost Always	Your score
**1. Incomplete Emptying**<br>How often have you had the sensation of not emptying your bladder?	0	1	2		4	5	
**2. Frequency**<br>How often have you had to urinate less than every two hours?	0	1	2	3	4	5	
**3. Intermittency**<br>How often have you found you stopped and started again several times when you urinated?	0	1	2	3	4	5	
**4. Urgency**<br>How often have you found it difficult to postpone urination?	0	1	2	3	4	5	
**5. Weak Stream**<br>How often have you had a weak urinary stream?	0	1	2	3	4	5	
**6. Straining**<br>How often have you had to strain to start urination?	0	1	2	3	4	5	
	**None**	**1 Time**	**2 Times**	**3 Times**	**4 Times**	**5 Times**	
**7. Nocturia**<br>How many times did you typically get up at night to urinate?	0	1	2	3	4	5	
**Total I-PASS Score**							

Score: 1-7: *Mild* 8-19: *Moderate* 20-35: *Severe*

**Quality of life(QoL) Due to Urinary Symptoms**	Delighted	Pleased	Mostly Satisfied	Mixed	Mostly Dissatisfied	Unhappy	Terrible

If you were to spend the rest of your life with your urinary condition just the way it is now, how would you feel about that?	**0**	**1**	**2**	**3**	**4**	**5**	**6**

Appendix 8: The International Index of Erectile Function Questionnaire (IIEF)

Please complete and bring this questionnaire to your appointment (circle your answers and add up the total).
The first five questions refer to erectile function

1. Over the last month, how often were you able to get an erection during sexual activity?

0 No sexual activity
5 Almost always or always
4 Most times (much more than half the time)
3 Sometimes (about half the time)
2 A few times (much less than half the time)
1 Almost never or never

Q2. Over the last month, when you had erections with sexual stimulation, how often were your erections hard enough for penetration?

0 No sexual activity
5 Almost always or always
4 Most times (much more than half the time)
3 Sometimes (about half the time)
2 A few times (much less than half the time)
1 Almost never or never

Q3. Over the last month, when you attempted intercourse, how often were you able to penetrate your partner?

0 No sexual activity
5 Almost always or always
4 Most times (much more than half the time)
3 Sometimes (about half the time)
2 A few times (much less than half the time)
1 Almost never or never

Q4. Over the last month, during sexual intercourse, how often were you able to maintain your erection after you had penetrated your partner?

0 No sexual activity
5 Almost always or always
4 Most times (much more than half the time)
3 Sometimes (about half the time)
2 A few times (much less than half the time)
1 Almost never or never

Q5. Over the last month, during sexual intercourse, how difficult was it to maintain your erection to completion of intercourse?

0 No sexual activity
1 Extremely difficult

2 Very difficult

3 Difficult

4 Slightly difficult

5 No difficult

The next three questions refer to satisfaction with intercourse

Q6. Over the last month, how many times have you attempted sexual intercourse?

0 No attempts

1 1-2 times

2 3-4 times

3 5-6 times

4 7-10 times

5 11-20 times

Q7. Over the last month, when you attempted sexual intercourse, how often was it satisfactory for you?

0 Did not attempt intercourse

5 Almost always or always

4 Most times (much more than half the time)

3 Sometimes (about half the time)

2 A few times (much less than half the time)

1 Almost never or never

Q8. Over the last month, how much have you enjoyed sexual intercourse?

0 No intercourse

5 Very highly enjoyable

4 Highly enjoyable

3 Fairly enjoyable

2 Not very enjoyable

1 No enjoyment

The next two questions refer to the orgasmic function

Q9. Over the last month, when you had sexual stimulation or intercourse, how often did you ejaculate?

0 No sexual stimulation/intercourse

5 Almost always or always

4 Most times (much more than half the time)

3 Sometimes (about half the time)

2 A few times (much less than half the time)

1 Almost never or never

Q10. Over the last month, when you had sexual stimulation or intercourse, how often did you have the feeling of orgasm (with or without ejaculation)?

0 No sexual stimulation/intercourse

5 Almost always or always

4 Most times (much more than half the time)

3 Sometimes (about half the time)

2 A few times (much less than half the time)

1 Almost never or never

The next two questions ask about sexual desire. In this context, sexual desire is defined as a feeling that may include wanting to have a sexual experience (for example, masturbation or sexual intercourse), thinking about having sex or feeling frustrated due to lack of sex.

Q11. Over the last month, how often have you felt sexual desire?

5 Almost always or always

4 Most times (much more than half the time)

3 Sometimes (about half the time)

2 A few times (much less than half the time)

1 Almost never or never

Q12. Over the last month, how would you rate your level of sexual desire?

5 Very high

4 High

3 Moderate

2 Low

1 Very low or not at all

The next two questions refer to overall sexual satisfaction.

Q13. Over the last month, how satisfied have you been with your overall sex life?

5 Very satisfied

4 Moderately satisfied

3 About equally satisfied and dissatisfied

2 Moderately dissatisfied

1 Very dissatisfied

Q14. Over the last month, how satisfied have you been with your sexual relationship with your partner?

5 Very satisfied

4 Moderately satisfied

3 About equally satisfied and dissatisfied

2 Moderately dissatisfied

1 Very dissatisfied

The last question refers to erectile function

Q15. Over the last month, how do you rate your confidence that you can get and keep your erection?

5 Very high

4 High

3 Moderate
2 Low
1 Very low

What the Scores Mean

All the questions break down into five specific areas, as follows. Add your scores to the appropriate column.

All the questions break down into five specific areas, as follows. Add your scores to the appropriate column.

Area	Questions	Score Range	Maximum Score	Your Score
Erectile Function	1-5 & 15	0-5	30	
Orgasmic Function	9-10	0-5	10	
Sexual Desire	11-12	1-5	10	
Intercourse Satisfaction	6-8	0-5	15	
Overall Satisfaction	13-14	1-5	10	

Appendix 9: The Female Sexual Function Index

INSTRUCTIONS: These questions ask about your sexual feelings and responses during the past 4 weeks. Please answer the following questions as honestly and clearly as possible. Your responses will be kept completely confidential. In answering these questions, the following definitions apply:

Sexual activity can include caressing, foreplay, masturbation, and vaginal intercourse.

Sexual intercourse is defined as penile penetration (entry) of the vagina.

Sexual stimulation includes situations like foreplay with a partner, self-stimulation (masturbation), or sexual fantasy.

CHECK ONLY ONE BOX PER QUESTION.

Sexual desire or interest is a feeling that includes wanting to have a sexual experience, feeling receptive to a partner's sexual initiation, and thinking or fantasizing about having sex.

1. Over the past 4 weeks, how **often** did you feel sexual desire or interest?
   - ☐ Almost always or always
   - ☐ Most times (more than half the time)
   - ☐ Sometimes (about half the time)
   - ☐ A few times (less than half the time)
   - ☐ Almost never or never
2. Over the past 4 weeks, how would you rate your **level** (degree) of sexual desire or interest?
   - ☐ Very high
   - ☐ High
   - ☐ Moderate
   - ☐ Low
   - ☐ Very low or none at all

Sexual arousal is a feeling that includes both physical and mental aspects of sexual excitement. It may include feelings of warmth or tingling in the genitals, lubrication (wetness), or muscle contractions.

3. Over the past 4 weeks, how **often** did you feel sexually aroused ("turned on") during sexual activity or intercourse?
   - ☐ No sexual activity
   - ☐ Almost always or always
   - ☐ Most times (more than half the time)
   - ☐ Sometimes (about half the time)
   - ☐ A few times (less than half the time)
   - ☐ Almost never or never
4. Over the past 4 weeks, how would you rate your **level** of sexual arousal ("turn on") during sexual activity or intercourse?
   - ☐ No sexual activity
   - ☐ Very high
   - ☐ High
   - ☐ Moderate
   - ☐ Low
   - ☐ Very low or none at all
5. Over the past 4 weeks, how **confident** were you about becoming sexually aroused during sexual activity or intercourse?
   - ☐ No sexual activity
   - ☐ Very high confidence
   - ☐ High confidence
   - ☐ Moderate confidence
   - ☐ Low confidence
   - ☐ Very low or no confidence
6. Over the past 4 weeks, how often have you been satisfied with your arousal (excitement) during sexual activity or intercourse?
   - ☐ No sexual activity
   - ☐ Almost always or always
   - ☐ Most times (more than half the time)
   - ☐ Sometimes (about half the time)
   - ☐ A few times (less than half the time)
   - ☐ Almost never or never
7. Over the past 4 weeks, how **often** did you become lubricated ("wet") during sexual activity or intercourse?
   - ☐ No sexual activity
   - ☐ Almost always or always
   - ☐ Most times (more than half the time)
   - ☐ Sometimes (about half the time)
   - ☐ A few times (less than half the time)
   - ☐ Almost never or never

8. Over the past 4 weeks, how **difficult** was it to become lubricated ("wet") during sexual activity or intercourse?
   - ☐ No sexual activity
   - ☐ Extremely difficult or impossible
   - ☐ Very difficult
   - ☐ Difficult
   - ☐ Slightly difficult
   - ☐ Not difficult
9. Over the past 4 weeks, how **often** did you maintain your lubrication ("wetness") until completion of sexual activity or intercourse?
   - ☐ No sexual activity
   - ☐ Almost always or always
   - ☐ Most times (more than half the time)
   - ☐ Sometimes (about half the time)
   - ☐ A few times (less than half the time)
   - ☐ Almost never or never
10. Over the past 4 weeks, how **difficult** was it to maintain your lubrication ("wetness") until completion of sexual activity or intercourse?
    - ☐ No sexual activity
    - ☐ Extremely difficult or impossible
    - ☐ Very difficult
    - ☐ Difficult
    - ☐ Slightly difficult
    - ☐ Not difficult
11. Over the past 4 weeks, when you had sexual stimulation or intercourse, how **often** did you reach orgasm (climax)?
    - ☐ No sexual activity
    - ☐ Almost always or always
    - ☐ Most times (more than half the time)
    - ☐ Sometimes (about half the time)
    - ☐ A few times (less than half the time)
    - ☐ Almost never or never
12. Over the past 4 weeks, when you had sexual stimulation or intercourse, how **difficult** was it for you to reach orgasm (climax)?
    - ☐ No sexual activity
    - ☐ Extremely difficult or impossible
    - ☐ Very difficult
    - ☐ Difficult
    - ☐ Slightly difficult
    - ☐ Not difficult

13. Over the past 4 weeks, how **satisfied** were you with your ability to reach orgasm (climax) during sexual activity or intercourse?
    - ☐No sexual activity
    - ☐Very satisfied
    - ☐Moderately satisfied
    - ☐About equally satisfied and dissatisfied
    - ☐Moderately dissatisfied
    - ☐Very dissatisfied
14. Over the past 4 weeks, how **satisfied** have you been with the amount of emotional closeness during sexual activity between you and your partner?
    - ☐No sexual activity
    - ☐Very satisfied
    - ☐Moderately satisfied
    - ☐About equally satisfied and dissatisfied
    - ☐Moderately dissatisfied
    - ☐Very dissatisfied
15. Over the past 4 weeks, how **satisfied** have you been with your sexual relationship with your partner?
    - ☐ Very satisfied
    - ☐ Moderately satisfied
    - ☐ About equally satisfied and dissatisfied
    - ☐ Moderately dissatisfied
    - ☐ Very dissatisfied
16. Over the past 4 weeks, how **satisfied** have you been with your overall sexual life?
    - ☐ Very satisfied
    - ☐ Moderately satisfied
    - ☐ About equally satisfied and dissatisfied
    - ☐ Moderately dissatisfied
    - ☐ Very dissatisfied
17. Over the past 4 weeks, how **often** did you experience discomfort or pain during vaginal penetration?
    - ☐Did not attempt intercourse
    - ☐Almost always or always
    - ☐Most times (more than half the time)
    - ☐Sometimes (about half the time)
    - ☐A few times (less than half the time)
    - ☐Almost never or never

18. Over the past 4 weeks, how **often** did you experience discomfort or pain following vaginal penetration?
    - ☐ Did not attempt intercourse
    - ☐ Almost always or always
    - ☐
    - ☐
    - ☐
    - ☐

Most times (more than half the time)

Sometimes (about half the time)

A few times (less than half the time)

Almost never or never

19. Over the past 4 weeks, how would you rate your **level** (degree) of discomfort or pain during or following vaginal penetration?

- ☐ Did not attempt intercourse
- ☐ Very high
- ☐ High
- ☐ Moderate
- ☐ Low
- ☐ Very low or none at all

Thank you for completing this questionnaire

Appendix 10: The Wexner score

Type of incontinence	***Frequency***				
	Never	***Rarely***	***Sometimes***	***Usually***	***Always***
Solid	0	1	2	3	4
Liquid	0	1	2	3	4
Gas	0	1	2	3	4
Wears pad	0	1	2	3	4
Lifestyle alteration	0	1	2	3	4

Never, 0; rarely, <1/month; sometimes, <1/week, >1/month; usually, <1/day, >1/week; always,>1/day. 0, perfect; 20, complete incontinence.

Appendix 11: TNM Staging

Table 1. Definitions for T, N, M

Primary Tumor (T)

TX Primary tumor cannot be assessed
T0 No evidence of primary tumor
Tis Carcinoma in situ: intraepithelial or invasion of lamina propria
T1 Tumor invades submucosa
T2 Tumor invades muscularis propria
T3 Tumor invades through the muscularis propria into the pericolorectal tissues
T4a Tumor penetrates to the surface of the visceral peritoneum
T4b Tumor directly invades or is adherent to other organs or structures

Regional Lymph Nodes (N)

NX Regional lymph nodes cannot be assessed
N0 No regional lymph node metastasis
N1 Metastasis in 1-3 regional lymph nodes
N1a Metastasis in one regional lymph node
N1b Metastasis in 2-3 regional lymph nodes
N1c Tumor deposit(s) in the subserosa, mesentery, or nonperitonealized pericolic or perirectal tissues without regional nodal metastasis
N2 Metastasis in four or more regional lymph nodes
N2a Metastasis in 4-6 regional lymph nodes
N2b Metastasis in seven or more regional lymph nodes

Distant Metastasis (M)

M0 No distant metastasis
M1 Distant metastasis
M1a Metastasis confined to one organ or site (e.g., liver, lung, ovary, nonregional node)
M1b Metastases in more than one organ/site or the peritoneum

Table 2. Anatomic Stage/Prognostic Groups

**Stage**	**T**	**N**	**M**	**Dukes***	**MAC***
0	Tis	N0	M0	-	-
I	T1	N0	M0	A	A
	T2	N0	M0	A	B1
IIA	T3	N0	M0	B	B2
IIB	T4a	N0	M0	B	B2
IIC	T4b	N0	M0	B	B3
IIIA	T1-T2	N1/N1c	M0	C	C1
	T1	N2a	M0	C	C1
IIIB	T3-T4a	N1/N1c	M0	C	C2
	T2-T3	N2a	M0	C	C1/C2
	T1-T2	N2b	M0	C	C1
IIIC	T4a	N2a	M0	C	C2
	T3-T4a	N2b	M0	C	C2
	T4b	N1-N2	M0	C	C3
IVA	Any T	Any N	M1a	-	-
IVB	Any T	Any N	M1b	-	-

Note: cTNM is the clinical classification, pTNM is the pathologic classification. The y prefix is used for those cancers that are classified after neoadjuvant pretreatment (e.g., ypTNM). Patients who have a complete pathologic response are ypT0N0cM0 that may be similar to Stage Group 0 or I. The r prefix is to be used for those cancers that have recurred after a disease-free interval (rTNM).

*Dukes B is a composite of better (T3 N0 M0) and worse (T4 N0 M0) prognostic groups, as is Dukes C (Any TN1 M0 and Any T N2 M0). MAC is the modified Astler-Coller classification

a Tis includes cancer cells confined within the glandular basement membrane (intraepithelial) or mucosal lamina propria (intramucosal) with no extension through the muscularis mucosae into the submucosa.

b Direct invasion in T4 includes invasion of other organs or other segments of the colorectum as a result of direct extension through the serosa, as confirmed on microscopic examination (for example,

invasion of the sigmoid colon by a carcinoma of the cecum) or, for cancers in a retroperitoneal or subperitoneal location, direct invasion of other organs or structures by virtue of extension beyond the muscularis propria (i.e., respectively, a tumor on the posterior wall of the descending colon invading the left kidney or lateral abdominal wall; or a mid or distal rectal cancer with invasion of the prostate, seminal vesicles, cervix, or vagina).
c Tumor that is adherent to other organs or structures, grossly, is classified cT4b. However, if no tumor is present in the adhesion, microscopically, the classification should be pT1-4a depending on the anatomical depth of wall invasion. The V and L classifications should be used to identify the presence or absence of vascular or lymphatic invasion, whereas the PN site-specific factor should be used for perineural invasion.

Laparoscopy-Assisted Surgery for Carcinoma of the Low Rectum: A Prospective, Multi-Center, Randomized, Open-Label, Parallel-Group, Non-Inferiority Clinical Trial (LASRE Trial)

Statistical Analysis Plan

Version 1.0

Authors: Jihan Huang, Jia Li, Fang Yin, Jingjing Wang, Hongxia Liu, Qingshan Zheng

Author for correspondence:
Dr. Jihan Huang

Center for Drug Clinical Research, Shanghai University of Traditional Chinese Medicine Shanghai, China;

Email: huangjihan@shutcm.edu.cn

Contents

1 Background..3

2 Objective...4

3 Study design..4

4 Inclusion criteria..4

5 Exclusion criteria...5

6 Surgical treatment plan...5

6.1 Open surgery...6

6.2 Laparoscopic surgery...6

7 Study endpoints..7

7.1 Primary outcome measure..7

7.2 Secondary outcome measures...7

7.3 Other pre-specified outcome measures..7

8 Statistical analyses...8

8.1 Sample size estimation...8

8.2 Analysis datasets..8

8.3 Statistical methods...9

8.4 Statistical software and general requirements.......................................10

9 Proposed format of statistical results in the publication of the main results............11

10 Statistical conclusions..29

11 References..29

List of abbreviations and definition of terms

Abbreviations	Definition of terms
**APR**	Abdominoperineal resection
**ASA**	American Society of Anesthesiologists
**BMI**	Body mass index
**CI**	Confidence interval
**CRMs**	Circumferential resection margins
**DFS**	Disease-free survival
**DRMs**	Distal resection margins
**ECOG**	Eastern Cooperative Oncology Group
**FSFI**	Female sexual function index
**HR**	Hazard ratio
**IIEF-5**	International Index of Erectile Function-5
**IMA**	Inferior mesenteric artery
**I-PSS**	International prostate symptom score
**IQR**	Interquartile range
**ISR**	Intersphincteric resection
**ITT**	Intention to treat
**LAR**	low anterior resection
**Max**	Maximum
**Min**	Minimum
**mITT**	Modified intention-to-treat
**OS**	Overall survival
**pCR**	Pathological complete response
**PPS**	Per-protocol set
**PRM**	Proximal resection margin
**QoL**	Quality of life
**SAE**	Serious adverse events
**SDs**	Standard deviations
**SS**	Safety set
**TME**	Total mesorectal excision
**ULAR**	Ultralow anterior resection

1 Background

According to the World Health Organization (WHO) report in 2005, there are 400,000 new colorectal cancer patients annually in China. Colorectal cancer has become the third-leading cause of cancer-related death in Chinese patients, with an annual mortality rate of 16.83 per 100,000. Low and mid rectal cancers account for approximately 70% to 80% of rectal cancers in China. Total mesorectal excision (TME) is still the primary treatment for rectal cancer. Recent studies have shown that laparoscopy-assisted resection is safe for treating low rectal cancer. It has the advantages of minimally invasive procedures: faster postoperative recovery, less pain, shorter hospital stay, and long-term efficacy comparable to open surgery.

Randomized control trials with large samples were launched to investigate laparoscopic and open surgery for colorectal cancer in European and North American countries, including the CLASSIC trial, the COLOR trial, and the COST study. These studies compared the completeness of tumor resection, long-term efficacy, and patient's Quality of life (QoL) between the two surgical approaches. The results showed that laparoscopic resection did not increase complications or operative mortality compared with open resection in treating rectal cancer. The two surgeries were comparable in the distance and negative rate of pathological resection margins (proximal, distal, and circumferential) and the number of dissected lymph nodes. Many meta-analyses have found no significant differences between the two surgical approaches in local recurrence rate, distant metastasis rate, or disease-free survival (DFS) rate.

Recent studies have also shown that laparoscopic total mesorectal excision (TME) for rectal cancer after preoperative chemoradiotherapy is safe without increasing the incidence of surgery-related complications or mortality. Moreover, with the accumulation of surgical experience and skills, the conversion rate from laparoscopic surgery to open surgery gradually decreases. However, the interpretation of these findings needs to be further discussed.

The 2013 National Comprehensive Cancer Network (NCCN) Guidelines v.4 for colon cancer recommended laparoscopic surgery to treat colon cancer, which should be performed by experienced surgeons. However, limited by the lack of data from randomized controlled studies with large samples, the 2013 NCCN Guidelines v.4 only recommended laparoscopic radical resection of rectal cancer for clinical research.

TME has been recognized as the "gold standard" for radical resection of mid and low rectal cancer. Due to the unique anatomical characteristics of low rectal cancer (within 5 cm from the dentate line), open surgery cannot avoid the disadvantage of the limited exposure to the pelvic floor. In contrast, the 30° lens of the laparoscope has advantages in visualizing the deep pelvic structure; in patients with low rectal cancer, the pelvic operating field can be visualized to ease the separation and nudization of the lower end of the rectum. The benefits of neoadjuvant chemoradiotherapy for patients with resectable cT3/T4aN0 M0 or cT1-4aN1-2M0 low-rectal cancer have not been fully confirmed. In these patients, laparoscopic surgery's safety and long-term efficacy still need to be evaluated and compared with open surgery.

ClinicalTrials.gov, an international clinical trial registry platform, reveals seven ongoing large-scale prospective randomized controlled trials concerning laparoscopic resection for rectal cancer across the world (Table 1). However, there is no randomized controlled trial of laparoscopic surgery, especially addressing low rectal cancer.

Thus, we designed this trial to assess the perioperative safety and long-term efficacy of laparoscopic surgery for low rectal cancer compared with open surgery. The findings from this study will provide high-level evidence for clinical decision-making for surgical treatment in patients with low rectal cancer.

2 Objective

To evaluate the perioperative safety and long-term efficacy of laparoscopy-assisted surgery for low rectal cancer compared to open surgery.

3 Study design

This study is a prospective, multicenter, randomized, open-label, parallel-group, non-inferiority clinical trial.

4 Inclusion criteria

1) Aged 18-75 years;

2) Pathological diagnosis of rectal adenocarcinoma (including highly and moderately differentiated tubular adenocarcinoma, papillary adenocarcinoma, poorly differentiated tubular adenocarcinoma, mucinous adenocarcinoma, and signet-ring cell carcinoma);

3) The lower margin of the tumor is < 5 cm from the dentate line at the time of initial diagnosis by rigid proctoscopy;

4) cT1-3N0-2M0 or cT4aN0-2M0 adenocarcinoma after neoadjuvant chemoradiotherapy. Patients with pelvic lateral lymph nodes are ineligible;

5) Primary tumor < 6 cm in size;

6) No other concurrent primary cancers;

7) Adequate function of main organs, allowing surgical treatment;

8) Patients and their family members can understand the study plan, are willing to participate, and agree to give written informed consent.

5 Exclusion criteria

1) Aged < 18 or > 75 years;

2) Concurrent or previous malignancies within five years;

3) Need for emergency surgery due to intestinal obstruction, intestinal perforation, intestinal hemorrhage, etc. ;

4) Previous history of colorectal surgery that might affect the reconstruction of the digestive tract;

5) Need to remove other organs in addition to the rectum;

6) ASA classification IV or V;

7) Current pregnancy or lactation:

- Women of childbearing age with a positive pregnancy test at baseline or who have not taken a pregnancy test; postmenopausal women must be at least 12 months postmenopausal;
- Sexually active men and women (of reproductive age) who are unwilling to take contraceptive measures during the study period;

8) Severe mental illness;

9) Inability to tolerate surgery due to severe emphysema, interstitial pneumonia, ischemic heart disease, etc.;

10) Continuous systemic steroid therapy within the last month;

11) Contraindications to laparoscopic surgery;

12) Patients and their family members cannot understand the conditions and objectives of this study.

6 Surgical treatment plan

6.1 Open surgery

This surgery includes standard TME and dissection of the No. 253 lymph nodes. The inferior mesenteric artery (IMA) is ligated either from its base directly or after the branching of the left colon artery. The distal resection margin (DRM) of the rectum is at least 1-2 cm away from the tumor. The surgical method is chosen as follows:

1) Transabdominal low anterior resection (LAR) or ultralow anterior resection (ULAR) may be chosen if the lower margin of the tumor is $\geq$ 2 cm from the proximal side of the anorectal ring;

2) In cT1 and T2 patients, as well as T3 patients whose external anal sphincters are not invaded by the tumor (including patients who have undergone neoadjuvant therapy), intersphincteric resection (ISR) through either the transpelvic approach or transanal approach may be chosen when the lower margin of tumor is < 2 cm from the proximal end of the anorectal ring;

3) cT1 to T3 tumors that are $\geq$ 5 cm from the anal verge are confined to the intestinal wall within a depth $\leq$3 cm and affect 1/2 of the circumferential wall of the rectum without invading the bladder, prostate, or vagina can be treated with ULAR via a pull-through procedure;

4) Abdominoperineal resection (APR) can be performed in patients with external anal sphincter invasion;

5) Extralevator abdominoperineal excision (ELAPE) can be performed via the pelvic approach in patients with levator ani muscle invasion;

For patients receiving sphincter preservation or partial sphincter preservation, loop transverse colostomy or ileostomy is recommended.

6.2 Laparoscopic surgery

The indications of laparoscopic operation are the same as those of open operation. The operation follows the principle of TME and includes the dissection of the No. 253 lymph nodes. The IMA is ligated either from its base directly or after the branching of the left colon artery.

The rectal DRM should be at least 1 to 2 cm away from the tumor. The principles for selecting the intestinal segmental resection and/or reconstruction methods are the same as in "**6.1 Open surgery**". Prophylactic transverse colostomy or ileostomy is recommended for patients who undergo anal sphincter–preserving surgery.

7 Study endpoints

7.1 Primary outcome measure

3-year DFS rate (final calculation is done 3 years after the last participant is enrolled)

7.2 Secondary outcome measures

■ Pathologic outcomes

Pathologic outcomes are defined as TME quality, negative CRM and negative DRM, length of proximal resection margin (PRM), length of DRM, and the number of retrieved lymph nodes.

■ 30-day postoperative complications

■ 30-day postoperative mortality

■ 3-year and 5-year OS rates (calculated 3 and 5 years after the last participant is enrolled)

■ Locoregional recurrence rate

7.3 Other pre-specified outcome measures

■ Operative outcomes:

1) Operation time (min)
2) Estimated blood loss (ml)
3) Conversion to open surgery (only applicable to the laparoscopic resection group)
4) Events of intraoperative complications

■ Postoperative recovery outcomes

1) Time to first flatus (h)

2) Time to the first defecation (h)
3) Time to first liquid food (h)
4) Time to normal diet (h)
5) Duration of analgesic use (h)
6) Length of postoperative hospital stay (d)

■ The quality of life (QoL)

☐ Quality of life (QoL) assessment: the EORTC QLQ C30 and EORTC QLQ CR29 scores

☐ Bladder function assessment:

- The residual urine volume (1st measurement: after the urethral catheter is removed on the fifth day after the operation, the residual urine volume in the bladder is determined by B-ultrasound after the patient urinates as much as possible for the first time)
- I-PSS

☐ Patient self-reported sexual function

Patient self-reported sexual function as assessed by the International Index of Erectile Function (IIEF-5) Female Sexual Function Index (FSFI) for male and female sexual function.

8 Statistical analyses

8.1 Sample size estimation

The sample size was estimated using a log-rank test based on a 3-year DFS with a noninferiority margin of 10%. At the time of this trial design, a noninferiority margin of 10% was determined to be clinically acceptable. Considering distinct prognoses in patients with stage I versus II/III disease, the sample size was calculated separately. Assumptions for the 3-year DFS rate in the open surgery group were 94.3% and 75.2% for clinical stage I and II/III diseases, respectively, with 359 patients with stage I disease (laparoscopic 240, open 119), 609 patients with stage II/III disease (laparoscopic 406, open 203), and a total of 968 patients (laparoscopic 646, open 322) were required to provide an 80% power with a one-sided α of 2.5%. One thousand sixty-five patients were aimed to enroll to allow for exclusions after

randomization to maintain the required statistical power. All calculations allowed for a 20% dropout. The planned and follow-up period were three years and five years, respectively.

8.2 Analysis datasets

(1) Modified intention-to-treat (mITT): The mITT population is defined as all randomized subjects except those excluded after randomization. The modified intention-to-treat (mITT) population will serve as the primary population for efficacy analyses in this study.

(2) Per-protocol set (PPS): The PPS includes patients who underwent the assigned surgery only. In other words, the participants who meet the trial plan, have good compliance, and complete the items specified in the eCRF are included in the per-protocol (PP) analysis. PP analysis is mainly applied to the primary outcome measure.

(3) Safety set (SS): The SS includes the actual data of participants who have received treatment and have records of safety indicators. The missing data of safety indicators cannot be obtained from carry-over from previous records. The dataset includes some patients excluded from the trial, e.g., those whose age exceeds the inclusion criteria. The incidence of adverse reactions is determined by using the number of cases in the SS as a denominator.

8.3 Statistical methods

8.3.1 Analysis Populations

(1) The number of enrolled patients and the number of patients who complete the trial in each center are listed to generate three analysis datasets (mITT, PPS, and SS).

(2) The patients who drop out and are excluded are listed, along with the reasons.

8.3.2 Demographic and baseline Characteristics

Demographic and baseline characteristic values were summarized using descriptive statistics:

(1) The number of cases, mean, standard deviation, median, and interquartile range (IQR), are calculated for continuous variables. Use means and standard deviations (SDs) for normally distributed data and medians and interquartile range (IQR) for data that are not normally distributed.

(2) The frequency and percentages are calculated for the categorical variables.

(3) The inferential statistical results (P values) are given as descriptive results.

8.3.3 Outcome analysis

- **Analysis of primary outcome measures**

The DFS is calculated using the Kaplan-Meier method after the last participant has been enrolled for three years. The median DFS time and its 95% confidence interval are compared between the two groups using the log-rank test. Cox regression is used to calculate the hazard ratio and its one-sided 95% confidence interval between the two groups. According to the prespecified noninferiority criteria, the inferiority of the treatment to be tested compared with the control treatment is determined.

- **Analysis of secondary outcome and other pre-specified outcome measures**

(1) Pathologic outcomes, including the TME quality, negative CRM, and negative DRM, are presented as numbers (percentage) and compared using the Chi-square test.

(2) The operation time (min) and estimated blood loss (ml) are compared between the groups by the Wilcoxon method.

(3) The OS is calculated using the Kaplan-Meier method after the last participant has been enrolled for three years. The median OS time and its 95% confidence interval are compared between the two groups using the log-rank test.

(4) The OS when the last participant is enrolled for five years is determined by using the Kaplan-Meier method. The median OS time and its 95% confidence interval are compared between the two groups using the log-rank test.

(5) Time to tumor recurrence is determined using the Kaplan-Meier method. The median time of recurrence and its 95% confidence interval are compared between the two groups using the log-rank test.

(6) The perioperative recovery time is compared between the groups using the Wilcoxon method.

(7) The length of postoperative hospital stay (d) is compared between the groups using the Wilcoxon method.

(8) The incidence of intraoperative complications is compared between the groups by the $\chi 2$ test or Fisher's exact probability method.

(9) After surgery, the incidence of complications and 30-day mortality rate are compared using the $\chi 2$ test or the Fisher's exact tests for probability.

8.3.4 Safety analysis

(1) The incidences of adverse events and adverse reactions are calculated.

(2) The frequency and number of adverse events and reactions will be presented by system organ class and preferred term (MedDRA).

8.4 Statistical software and general requirements

(1) SAS 9.4 is used for analysis (SAS Institute Inc., Cary, NC, USA).

(2) Generally, two-sided tests are used for statistical analysis, and a P value < 0.05 indicates that the difference is statistically significant.

9 Proposed format of statistical results in the publication of the main results

Table 1. Summary of Analysis Population All Subjects Randomized

	Laparoscopic Surgery Group	Open Surgery Group	Total
**Enrollment subjects**	XXX	XXX	XXX
**Complete subjects**	XXX	XXX	XXX
**mITT subjects**	XXX	XXX	XXX
**PPS subjects**	XXX	XXX	XXX
**SS subjects**	XXX	XXX	XXX

Table 2. List of cases not included in mITT (enrolled subjects)

Group	Center	Random number	Clinical stage	Reason	mITT	PPS	SS
XXX	XXX	XXX	XXX	XXX	XXX	XXX	XXX
...	...	...	...	...	...	...	...
...	...	...	...	...	...	...	...
...	...	...	...	...	...	...	...
XXX	XXX	XXX	XXX	XXX	XXX	XXX	XXX

Table 3. Demographic and Baseline Characteristics

Characteristics	Laparoscopic Surgery Group (n=XXX)	Open Surgery Group (n=XXX)	*P* value
**Male, n (%)**	XXX(XX.X)	XXX(XX.X)	X.XXX
**Age, median (IQR), year**	XXX (XX.X–XX.X)	XX (XX.X–XX.X)	X.XXX
**BMI, median (IQR), kg/m^2**	XX (XX.X–XX.X)	XX (XX.X–XX.X)	X.XXX
BMI <25.0 kg/m^2, n (%)	XXX(XX.X)	XXX(XX.X)	X.XXX
25.0 kg/m^2≤BMI<30.0 kg/m^2, n (%)	XXX(XX.X)	XXX(XX.X)	X.XXX
BMI≥30.0 kg/m^2, n (%)	XXX(XX.X)	XXX(XX.X)	
**ECOG score, n (%)**			X.XXX
0	XXX(XX.X)	XXX(XX.X)	
1	XXX(XX.X)	XXX(XX.X)	
2	XXX(XX.X)	XXX(XX.X)	
**ASA score, n (%)**			X.XXX
I	XXX(XX.X)	XXX(XX.X)	
II	XXX(XX.X)	XXX(XX.X)	
III	XXX(XX.X)	XXX(XX.X)	
**Comorbidity, n (%)**			X.XXX
Yes	XXX(XX.X)	XXX(XX.X)	
No	XXX(XX.X)	XXX(XX.X)	
**Tumor distance from dentate line, median (IQR), mm**	XX (XX.X–XX.X)	XX (XX.X–XX.X)	X.XXX
**Clinical TNM stage, n (%)**			X.XXX
I	XXX(XX.X)	XXX(XX.X)	
II/III	XXX(XX.X)	XXX(XX.X)	
**Preoperative therapy, n (%)**			X.XXX
Chemoradiotherapy	XXX(XX.X)	XXX(XX.X)	
Radiotherapy alone	XXX(XX.X)	XXX(XX.X)	
Chemotherapy alone	XXX(XX.X)	XXX(XX.X)	
Without Chemoradiotherapy	XXX(XX.X)	XXX(XX.X)	

Table 4. Surgical Details

Characteristics	Laparoscopic Surgery Group (n=XXX)	Open Surgery Group (n=XXX)	*P* value
**Conversion to open resection, n (%)**	XXX(XX.X)	-	-
**Operative time, median (IQR), min**	XX (XX.X–XX.X)	XX (XX.X–XX.X)	X.XXX
**Combined resection, n (%)**	XXX(XX.X)	XXX(XX.X)	
Uterus	X	X	
Ovaries	X	X	
Seminal vesicle	X	X	
Vagina	X	X	
Bladder	X	X	
...	X	X	
...	X	X	
...	X	X	
**Estimated blood loss, median (IQR), mL**	XX (XX.X–XX.X)	XX (XX.X–XX.X)	X.XXX
**Intraoperative complications No. (%)**	XXX(XX.X)	XXX(XX.X)	X.XXX
Bleeding	XXX(XX.X)	XXX(XX.X)	
Presacral hemorrhage	XXX(XX.X)	XXX(XX.X)	
Rectum perforation	XXX(XX.X)	XXX(XX.X)	
Urinary tract injury	XXX(XX.X)	XXX(XX.X)	
...	XXX(XX.X)	XXX(XX.X)	
...	XXX(XX.X)	XXX(XX.X)	
...	XXX(XX.X)	XXX(XX.X)	
**Type of surgery, n (%)**			X.XXX
LAR	XXX(XX.X)	XXX(XX.X)	
ISR	XXX(XX.X)	XXX(XX.X)	
APR	XXX(XX.X)	XXX(XX.X)	
Others	XXX(XX.X)	XXX(XX.X)	
**Sphincter preserving, n (%)**	XXX(XX.X)	XXX(XX.X)	X.XXX
**Diverting ostomy, n (%)**			X.XXX
Yes	XXX(XX.X)	XXX(XX.X)	
No	XXX(XX.X)	XXX(XX.X)	

Table 5. Quality of TME and Pathologic Outcomes

Characteristics	Laparoscopic Surgery Group (n=XXX)	Open Surgery Group (n=XXX)	Difference,% (95% CI)	*P* value
**Quality of TME, n (%)**				X.XX X
Complete	XXX(XX.X)	XXX(XX.X)	XX (XX.X–XX.X)	
Near-complete	XXX(XX.X)	XXX(XX.X)	XX (XX.X–XX.X)	
Incomplete	XXX(XX.X)	XXX(XX.X)	XX (XX.X–XX.X)	
**Quality of TME for stage I, n (%)**				X.XXX
Complete	XXX(XX.X)	XXX(XX.X)	XX (XX.X–XX.X)	
Near-complete	XXX(XX.X)	XXX(XX.X)	XX (XX.X–XX.X)	
Incomplete	XXX(XX.X)	XXX(XX.X)	XX (XX.X– XX.X)	
**Quality of TME or stage II/III, n (%)**				X.XXX
Complete	XXX(XX.X)	XXX(XX.X)	XX (XX.X–XX.X)	
Near-complete	XXX(XX.X)	XXX(XX.X)	XX (XX.X–XX.X)	
Incomplete	XXX(XX.X)	XXX(XX.X)	XX (XX.X–XX.X)	
**Length of PRM, median (IQR), mm**	XX (XX.X–XX.X)	XX (XX.X–XX.X)		X.XXX
Overall group				
Stage I disease				
Stage II/III disease				
**Length of DRM, median (IQR), mm**	XX (XX.X–XX.X)	XX (XX.X–XX.X)		X.XXX
Overall group				
Stage I disease				
Stage II/III disease				
**Lymph nodes dissected, median (IQR)**	XX (XX.X–XX.X)	XX (XX.X–XX.X)		X.XXX
Overall group				
Stage I disease				
Stage II/III disease				
**Negative resection margin, n (%)**				
**Negative PRMs, No. (%)**				X.XXX
Overall group	XXX(XX.X)	XXX(XX.X)		
Stage I disease	XXX(XX.X)	XXX(XX.X)		

Stage II/III disease	XXX(XX.X)	XXX(XX.X)		
**Negative DRMs, No. (%)**				X.XXX
Overall group	XXX(XX.X)	XXX(XX.X)		
Stage I disease	XXX(XX.X)	XXX(XX.X)		
Stage II/III disease	XXX(XX.X)	XXX(XX.X)		
**Negative CRMs, No. (%)**				X.XXX
Overall group	XXX(XX.X)	XXX(XX.X)		
Stage I disease	XXX(XX.X)	XXX(XX.X)		
Stage II/III disease	XXX(XX.X)	XXX(XX.X)		
**Pathologic T stage, n (%)**				X.XXX
T0/Tis	XXX(XX.X)	XXX(XX.X)		
T1	XXX(XX.X)	XXX(XX.X)		
T2	XXX(XX.X)	XXX(XX.X)		
T3	XXX(XX.X)	XXX(XX.X)		
T4a	XXX(XX.X)	XXX(XX.X)		
T4b	XXX(XX.X)	XXX(XX.X)		
**Pathologic N stage, n (%)**				X.XXX
N0	XXX(XX.X)	XXX(XX.X)		
N1a	XXX(XX.X)	XXX(XX.X)		
N1b	XXX(XX.X)	XXX(XX.X)		
N1c	XXX(XX.X)	XXX(XX.X)		
N2a	XXX(XX.X)	XXX(XX.X)		
N2b	XXX(XX.X)	XXX(XX.X)		
**Pathologic TNM stage, n (%)**				X.XXX
0/pCR	XXX(XX.X)	XXX(XX.X)		
I	XXX(XX.X)	XXX(XX.X)		
IIA	XXX(XX.X)	XXX(XX.X)		
IIB	XXX(XX.X)	XXX(XX.X)		
IIC	XXX(XX.X)	XXX(XX.X)		
IIIA	XXX(XX.X)	XXX(XX.X)		
IIIB	XXX(XX.X)	XXX(XX.X)		
IIIC	XXX(XX.X)	XXX(XX.X)		
IVA	XXX(XX.X)	XXX(XX.X)		
IVB	XXX(XX.X)	XXX(XX.X)		

Table 6. Postoperative Recovery and Complications

Characteristics	Laparoscopic Surgery Group (n=XXX)	Open Surgery Group (n=XXX)	*Difference, % (95% CI)*	*P* value
**Postoperative recovery**				
Time to first flatus, median (IQR), h	XX (XX.X–XX.X)	XX (XX.X–XX.X)		X.XXX
Time to first defecation, median (SD), h	XX (XX.X–XX.X)	XX (XX.X–XX.X)		X.XXX
Time to liquid diet, median (IQR), h	XX (XX.X–XX.X)	XX (XX.X–XX.X)		X.XXX
Time to resumption of normal diet, median (IQR), h	XX (XX.X–XX.X)	XX (XX.X–XX.X)		X.XXX
Time to analgesic requirement, median (IQR), h	XX (XX.X–XX.X)	XX (XX.X–XX.X)		X.XXX
Postoperative hospital stays, median (IQR), d	XX (XX.X–XX.X)	XX (XX.X–XX.X)		X.XXX
**Postoperative 30-day complications, n (%)**	XXX(XX.X)	XXX(XX.X)		X.XXX
Presacral hemorrhage	XXX(XX.X)	XXX(XX.X)	XX (XX.X–XX.X)	X.XXX
Active intraabdominal bleeding	XXX(XX.X)	XXX(XX.X)	XX (XX.X–XX.X)	X.XXX
Anastomotic bleeding	XXX(XX.X)	XXX(XX.X)	XX (XX.X–XX.X)	X.XXX
Anastomotic leakage	XXX(XX.X)	XXX(XX.X)	XX (XX.X–XX.X)	X.XXX
Chylous leakage	XXX(XX.X)	XXX(XX.X)	XX (XX.X–XX.X)	X.XXX
Ileus	XXX(XX.X)	XXX(XX.X)	XX (XX.X–XX.X)	X.XXX
Incision complications	XXX(XX.X)	XXX(XX.X)	XX (XX.X–XX.X)	X.XXX
Stoma-related complications	XXX(XX.X)	XXX(XX.X)	XX (XX.X–XX.X)	X.XXX
Urinary disorder	XXX(XX.X)	XXX(XX.X)	XX (XX.X–XX.X)	X.XXX
Urinary tract infection	XXX(XX.X)	XXX(XX.X)	XX (XX.X–XX.X)	X.XXX
Cardiovascular event	XXX(XX.X)	XXX(XX.X)	XX (XX.X–XX.X)	X.XXX
Pneumonia	XXX(XX.X)	XXX(XX.X)	XX (XX.X–XX.X)	X.XXX
...	XXX(XX.X)	XXX(XX.X)	XX (XX.X–XX.X)	X.XXX
**Postoperative 30-day mortality, n (%)**	XXX(XX.X)	XXX(XX.X)	XXX(XX.X)	X.XXX
**Clavien-Dindo classification**				X.XXX
I	XXX(XX.X)	XXX(XX.X)	XX (XX.X–XX.X)	
II	XXX(XX.X)	XXX(XX.X)	XX (XX.X–XX.X)	
IIIa	XXX(XX.X)	XXX(XX.X)	XX (XX.X–XX.X)	
IIIb	XXX(XX.X)	XXX(XX.X)	XX (XX.X–XX.X)	
IVa	XXX(XX.X)	XXX(XX.X)	XX (XX.X–XX.X)	
IVb	XXX(XX.X)	XXX(XX.X)	XX (XX.X–XX.X)	

V	XXX(XX.X)	XXX(XX.X)	XX (XX.X–XX.X)	

Table 7. The DFS (mon) for Laparoscopic Surgery vs Open Surgery within 3 Years after Surgery (mITT)

	Laparoscopic Surgery Group	Open Surgery Group	*P* value
**N**	XXX	XXX	X.XXX
**Censored(%)**	XX(XX.X)	XX(XX.X)	
**Events(%)**	XX(XX.X)	XX(XX.X)	
**25%(95%CI)**	XX.X (XX.X~XX.X)	XX.X (XX.X~XX.X)	
**50%(95%CI)**	XX.X (XX.X~XX.X)	XX.X (XX.X~XX.X)	
**75%(95%CI)**	XX.X (XX.X~XX.X)	XX.X (XX.X~XX.X)	

Table 8. The DFS (mon) for Laparoscopic Surgery vs Open Surgery within 3 Years after Surgery (PPS)

	Laparoscopic Surgery Group	Open Surgery Group	*P* value
**N**	XXX	XXX	X.XXX
**Censored(%)**	XX(XX.X)	XX(XX.X)	
**Events(%)**	XX(XX.X)	XX(XX.X)	
**25%(95%CI)**	XX.X (XX.X~XX.X)	XX.X (XX.X~XX.X)	
**50%(95%CI)**	XX.X (XX.X~XX.X)	XX.X (XX.X~XX.X)	
**75%(95%CI)**	XX.X (XX.X~XX.X)	XX.X (XX.X~XX.X)	

Figure 1. Kaplan–Meier curve for comparison of 3-year DFS rate between two groups (mITT)

Figure 2. Kaplan–Meier curve for comparison of 3-year DFS rate between two groups (PPS)

Table 9. Cox Regression Model Analysis of DFS (mITT)

		DF	HR	95%CIL	95%CIU	*P* value
**Group**	Laparoscopic Surgery vs Open Surgery	X	X.XX	X.XX	X.XX	X.XXX
**Center**	Center 1 vs Center X	X	X.XX	X.XX	X.XX	X.XXX
	Center 2 vs Center X	X	X.XX	X.XX	X.XX	X.XXX
	Center…vs Center X	X	X.XX	X.XX	X.XX	X.XXX
**XXX**		X	X.XX	X.XX	X.XX	X.XXX

Table 10. The OS (mon) for Laparoscopic Surgery vs Open Surgery within 3 Years after Surgery(mITT)

	Laparoscopic Surgery Group	Open Surgery Group	*P* value
**N**	XXX	XXX	X.XXX
**Censored(%)**	XX(XX.X)	XX(XX.X)	
**Events(%)**	XX(XX.X)	XX(XX.X)	
**25%(95%CI)**	XX.X (XX.X~XX.X)	XX.X (XX.X~XX.X)	
**50%(95%CI)**	XX.X (XX.X~XX.X)	XX.X (XX.X~XX.X)	
**75%(95%CI)**	XX.X (XX.X~XX.X)	XX.X (XX.X~XX.X)	

Figure 3. Kaplan–Meier curve for comparison of 3-year overall survival rate between two groups (mITT)

Table 11. The OS (mon) for Laparoscopic Surgery vs Open Surgery within 5 Years after Surgery(mITT)

	Laparoscopic Surgery Group	Open Surgery Group	*P* value
**N**	XXX	XXX	X.XXX
**Censored(%)**	XX(XX.X)	XX(XX.X)	
**Events(%)**	XX(XX.X)	XX(XX.X)	
**25%(95%CI)**	XX.X (XX.X~XX.X)	XX.X (XX.X~XX.X)	
**50%(95%CI)**	XX.X (XX.X~XX.X)	XX.X (XX.X~XX.X)	
**75%(95%CI)**	XX.X (XX.X~XX.X)	XX.X (XX.X~XX.X)	

Figure 4. Kaplan–Meier curve for comparison of 5-year overall survival rate between two groups (mITT)

Table 12. Time to Tumor Recurrence (mon) (mITT)

	Laparoscopic Surgery Group	Open Surgery Group	*P* value
**N**	XXX	XXX	X.XXX
**Censored(%)**	XX(XX.X)	XX(XX.X)	
**Events(%)**	XX(XX.X)	XX(XX.X)	
**25%(95%CI)**	XX.X (XX.X~XX.X)	XX.X (XX.X~XX.X)	
**50%(95%CI)**	XX.X (XX.X~XX.X)	XX.X (XX.X~XX.X)	
**75%(95%CI)**	XX.X (XX.X~XX.X)	XX.X (XX.X~XX.X)	

Figure 5. Kaplan–Meier curve for comparison of tumor recurrence rate between two groups (mITT)

Table 13. Analysis of the Incidence of Adverse Events and Adverse Reactions (SS)

	Laparoscopic Surgery Group	Open Surgery Group	*P* value
**N**	XXX	XXX	
**Adverse events, n(%)**	XX(XX.X)	XX(XX.X)	X.XXX
**Adverse reactions, n(%)**	XX(XX.X)	XX(XX.X)	X.XXX
**SAE, n(%)**	XX(XX.X)	XX(XX.X)	X.XXX

Table14. Summary of Number (%) of Subjects with Adverse Events by System Organ Class (SS)

**SOC**	**Laparoscopic Surgery Group**		**Open Surgery Group**	
PT	Number of cases	Number of occurrences	Number of cases	Number of occurrences
N	XXX		XXX	
Total ,n(%)	XXX(XX.X)	XXX	XXX(XX.X)	XXX
SOC1 n(%)	XXX(XX.X)	XXX	XXX(XX.X)	XXX
PT1 n(%)	XXX(XX.X)	XXX	XXX(XX.X)	XXX
PT2 n(%)	XXX(XX.X)	XXX	XXX(XX.X)	XXX
…				
SOC2	XXX(XX.X)	XXX	XXX(XX.X)	XXX
PT1 n(%)	XXX(XX.X)	XXX	XXX(XX.X)	XXX
PT2 n(%)	XXX(XX.X)	XXX	XXX(XX.X)	XXX
…				
SOC3	XXX(XX.X)	XXX	XXX(XX.X)	XXX
PT1 n(%)	XXX(XX.X)	XXX	XXX(XX.X)	XXX
PT2 n(%)	XXX(XX.X)	XXX	XXX(XX.X)	XXX
…				

Table 15. Summary of Number (%) of Subjects with Adverse Reactions by System Organ Class (SS)

**SOC**	**Laparoscopic Surgery Group**		**Open Surgery Group**	
PT	Number of cases	Number of occurrences	Number of cases	Number of occurrences
N	XXX		XXX	
Total n(%)	XXX(XX.X)	XXX	XXX(XX.X)	XXX
SOC1 n(%)	XXX(XX.X)	XXX	XXX(XX.X)	XXX
PT1 n(%)	XXX(XX.X)	XXX	XXX(XX.X)	XXX
PT2 n(%)	XXX(XX.X)	XXX	XXX(XX.X)	XXX
…				
SOC2	XXX(XX.X)	XXX	XXX(XX.X)	XXX
PT1 n(%)	XXX(XX.X)	XXX	XXX(XX.X)	XXX
PT2 n(%)	XXX(XX.X)	XXX	XXX(XX.X)	XXX
…				
SOC3	XXX(XX.X)	XXX	XXX(XX.X)	XXX
PT1 n(%)	XXX(XX.X)	XXX	XXX(XX.X)	XXX
PT2 n(%)	XXX(XX.X)	XXX	XXX(XX.X)	XXX
…				

10 Statistical conclusions

11 References

1. World Health Organization. WHO Mortality Database. Available at: http://www. Dep. iarc.fr/. Accessed January 15, 2011.
2. Heald RJ, Husband EM, Ryall RD. The mesorectum in rectal cancer surgery--the clue to pelvic recurrence?. Br J Surg. 1982;69(10):613-616. doi:10.1002/bjs. 1800691019.
3. Wang JP. Diagnosis and treatment of middle-low rectal cancer in the status quo and outlook. Chinese Journal of Practical Surgery, 2009, 29(4):287-290.
4. Guillou PJ, Quirke P, Thorpe H, et al. Short-term endpoints of conventional versus laparoscopic-assisted surgery in patients with colorectal cancer (MRC CLASICC trial): multicentre, randomised controlled trial. Lancet. 2005;365(9472):1718-1726. doi:10.1016/S0140-6736(05)66545-2.
5. Jayne DG, Brown JM, Thorpe H, et al. bladder and sexual function following resection for rectal cancer in a randomized clinical trial of laparoscopic versus open technique. Br J Surg. 2005;92(9):1124-1132. doi:10.1002/bjs.4989.
6. Jayne DG, Guillou PJ, Thorpe H, et al. Randomized trial of laparoscopic-assisted resection of colorectal carcinoma: 3-year results of the UK MRC CLASICC Trial Group. J Clin Oncol. 2007;25(21):3061-3068. doi:10.1200/JCO.2006.09.7758.
7. Jayne DG, Thorpe HC, Copeland J, et al. Five-year follow-up of the Medical Research Council CLASICC trial of laparoscopically assisted versus open surgery for colorectal cancer. Br J Surg. 2010;97(11):1638-1645. doi:10.1002/bjs.7160.
8. Taylor GW, Jayne DG, Brown SR, et al. Adhesions and incisional hernias following laparoscopic versus open surgery for colorectal cancer in the CLASICC trial. Br J Surg. 2010;97(1):70-78. doi:10.1002/bjs.6742.
9. Ng SS, Leung KL, Lee JF, et al. Laparoscopic-assisted versus open abdominoperineal resection for low rectal cancer: a prospective randomized trial [published correction appears in Ann Surg Oncol. 2009 Jan;16(1):229]. Ann Surg Oncol. 2008;15(9):2418-2425. doi:10.1245/s10434-008-9895-0.
10. Miyajima N, Fukunaga M, Hasegawa H, et al. Results of a multicenter study of 1,057 cases of rectal cancer treated by laparoscopic surgery. Surg Endosc. 2009;23(1):113-118. doi:10.1007/s00464-008-0078-7.
11. Gao F, Cao YF, Chen LS. Meta-analysis of short-term outcomes after laparoscopic resection for rectal cancer. Int J Colorectal Dis. 2006;21(7):652-656. doi:10.1007/ s00384-005-0079-0.
12. Xu ZR, Chi P. Comparison of the incidence of postoperative complications following laparoscopic and open colorectal cancer resection [J]. Chinese Journal of Gastrointestinal Surgery, 2012, 15(8):810-813.

13. Aziz O, Constantinides V, Tekkis PP, et al. Laparoscopic versus open surgery for rectal cancer: a meta-analysis. Ann Surg Oncol. 2006;13(3):413-424. doi:10.1245/ASO. 2006. 05.045.
14. Laurent C, Leblanc F, Bretagnol F, et al. Long-term wound advantages of the laparoscopic approach in rectal cancer. Br J Surg. 2008;95(7):903-908. doi:10.1002/bjs.6134.
15. Morino M, Allaix ME, Giraudo G, et al. Laparoscopic versus open surgery for extraperitoneal rectal cancer: a prospective comparative study. Surg Endosc. 2005;19(11):1460-1467. doi:10.1007/s00464-004-2001-1.
16. Lezoche E, Guerrieri M, De Sanctis A, et al. Long-term results of laparoscopic versus open colorectal resections for cancer in 235 patients with a minimum follow-up of 5 years. Surg Endosc. 2006;20(4):546-553. doi:10.1007/s00464-005-0338-8.
17. Li S, Chi P, Lin H, et al. Long-term outcomes of laparoscopic surgery versus open resection for middle and lower rectal cancer: an NTCLES study. Surg Endosc. 2011;25(10):3175-3182. doi:10.1007/s00464-011-1683-4.
18. Breukink SO, Pierie JP, Grond AJ, et al. Laparoscopic versus open total mesorectal excision: a case-control study. Int J Colorectal Dis. 2005;20(5):428-433. doi:10.1007/s00384-004-0715-0.
19. Bretagnol F, Lelong B, Laurent C, et al. The oncological safety of laparoscopic total mesorectal excision with sphincter preservation for rectal carcinoma. Surg Endosc. 2005;19(7):892-896. doi:10.1007/s00464-004-2228-x.
20. Anderson C, Uman G, Pigazzi A. Oncologic outcomes of laparoscopic surgery for rectal cancer: a systematic review and meta-analysis of the literature. Eur J Surg Oncol. 2008;34(10):1135-1142. doi:10.1016/j.ejso.2007.11.015.
21. Liang Y, Li G, Chen P, et al. Laparoscopic versus open colorectal resection for cancer: a meta-analysis of results of randomized controlled trials on recurrence. Eur J Surg Oncol. 2008;34(11):1217-1224. doi:10.1016/j.ejso.2007.11.004.
22. Konishi T, Watanabe T, Kishimoto J, et al. Elective colon and rectal surgery differ in risk factors for wound infection: results of prospective surveillance. Ann Surg. 2006;244(5):758-763. doi:10.1097/01.sla.0000219017.78611.49.
23. Lin HY, Chi P. Comparison of urinary function and sexual function between laparoscopic and open radical resection for rectal cancer [J]. Chinese Journal of Gastrointestinal Surgery, 2011, 14(4):289-290.
24. Zheng MH, Hu YY, Lu AG, et al. Clinical comparison of laparoscopic and open total mesorectal excision for lower rectal cancer. Chinese Journal of Gastrointestinal Surgery, 2004, 7(3): 177-180.
25. Zheng MH. Thoughts on the current situation of minimally invasive gastrointestinal surgery. Chinese Journal of Digestive Surgery, 2012,11(1): 22-24.

26. Zheng MH. Whether laparoscopic operation will be the gold standard for the surgical treatment of gastrointestinal neoplasms. Chinese Journal of Digestive Surgery, 2012,11(3):161-164.
27. American College of Surgeons. Laparoscopic-Assisted Resection or Open Resection in Treating Patients With Stage IIA, Stage IIIA, or Stage IIIB Rectal Cancer. http://clinicaltrials.gov/ct2/show/NCT00726622. It was accessed on September 2, 2011.
28. COLOR II: Laparoscopic Versus Open Rectal Cancer Removal. http://clinicaltrials.gov/ct2/show/ NCT00297791. Accessed September 2, 2011.
29. Kang SB, Park JW, Jeong SY, et al. Open versus laparoscopic surgery for mid or low rectal cancer after neoadjuvant chemoradiotherapy (COREAN trial): short-term outcomes of an open-label randomised controlled trial. Lancet Oncol, 2010,11:637-645.
30. National Comprehensive Cancer Network. NCCN Clinical Practice Guidelines in Oncology, Rectal Cancer, Version 4.2013.https//www.nccn.org/professionals /physician_gls/pdf/rectal.pdf [accessed 1 August 2013].
31. Dindo D, Demartines N, Clavien PA. Classification of surgical complications: a new proposal with evaluation in a cohort of 6336 patients and results of a survey. Ann Surg. 2004;240(2):205-213. doi:10.1097/01.sla.0000133083.54934. ae.
32. Nagtegaal ID, van de Velde CJ, van der Worp E, et al. Macroscopic evaluation of rectal cancer resection specimen: clinical significance of the pathologist in quality control. J Clin Oncol. 2002;20(7):1729-1734. doi:10.1200/JCO.2002.07.010.
33. Watanabe T, Itabashi M, Shimada Y, et al. Japanese Society for Cancer of the Colon and Rectum (JSCCR) guidelines 2010 for the treatment of colorectal cancer. Int J Clin Oncol. 2012;17(1):1-29. doi:10.1007/s10147-011-0315-2.
34. Hofheinz RD, Wenz F, Post S, et al. Chemoradiotherapy with capecitabine versus fluorouracil for locally advanced rectal cancer: a randomised, multicentre, non-inferiority, phase 3 trial. Lancet Oncol. 2012;13(6):579-588. doi:10.1016/ S1470-2045(12)70116-X.
35. Oken MM, Creech RH, Tormey DC, et al. Toxicity and response criteria of the Eastern Cooperative Oncology Group. Am J Clin Oncol. 1982;5(6):649-655.
36. Dripps RD, Lamont A, Eckenhoff JE. The role of anesthesia in surgical mortality. JAMA. 1961;178:261-266. doi:10.1001/jama.1961.03040420001001.
37. Dworak O, Keilholz L, Hoffmann A. Pathological features of rectal cancer after preoperative radiochemotherapy. Int J Colorectal Dis. 1997;12(1):19-23. doi:10.1007/s003840050072.
38. Aaronson NK, Ahmedzai S, Bergman B, et al. The European Organization for Research and Treatment of Cancer QLQ-C30: a quality-of-life instrument for use in international clinical trials in oncology. J Natl Cancer Inst. 1993;85(5):365-376. doi:10.1093/jnci/85.5.365.

39. Gujral S, Conroy T, Fleissner C, et al. Assessing quality of life in patients with colorectal cancer: an update of the EORTC quality of life questionnaire. Eur J Cancer. 2007;43(10):1564-1573. doi:10.1016/j.ejca.2007.04.005.
40. Barry MJ, Fowler FJ Jr, O'Leary MP, et al. The American Urological Association symptom index for benign prostatic hyperplasia. The Measurement Committee of the American Urological Association. J Urol. 1992;148(5):1549-1564. doi:10.1016/s0022-5347(17)36966-5.
41. Rosen RC, Riley A, Wagner G, Osterloh IH, Kirkpatrick J, Mishra A. The international index of erectile function (IIEF): a multidimensional scale for assessment of erectile dysfunction. Urology. 1997 Jun;49(6):822-30. doi: 10.1016/s0090-4295(97)00238-0. PMID: 9187685.
42. Rosen R, Brown C, Heiman J, et al. The Female Sexual Function Index (FSFI): a multidimensional self-report instrument for the assessment of female sexual function. J Sex Marital Ther. 2000;26(2):191-208. doi:10.1080/009262300278597.
43. Jorge JM, Wexner SD. Etiology and management of fecal incontinence. Dis Colon Rectum. 1993;36(1):77-97. doi:10.1007/BF02050307.
