# Supplementary material for: Short-term Outcomes of Laparoscopy-Assisted vs Open Surgery for Patients With Low Rectal Cancer: The LASRE Randomized Clinical Trial
Source: JAMA Oncol. 2022 Sep 15;8(11):1607–15. doi: 10.1001/jamaoncol.2022.4079 (PMC9478880; doi:10.1001/jamaoncol.2022.4079)
Supplement: Supplement 3. — LASRE trial investigators [file jamaoncol-e224079-s003.pdf]

Supplemental Online Content: Nonauthor Collaborators
*First name, last name, and suffix (if applicable) are required and will appear in PubMed.

*Group Name(s): LASRE trial investigators							
***First Name and Middle Initial(s)**	***Last Name**	***Suffix (eg, Jr, III)**	Academic Degrees	Institution	Location (city, state/province, country)	Role or Contribution, eg, chair, principal investigator	Group (if more than 1 Group listed in the byline) and/or Subgroup (eg, Steering Committee)
Guo-Dong	He		MD	Department of General Surgery, Zhongshan Hospital, Fudan University	Shanghai, China	assistant	LASRE Trial Investigators
Zhen-Dan	Yao		MD	Key Laboratory of Carcinogenesis and Translational Research, Ministry of Education, Gastrointestinal Cancer Center, Peking University Cancer Hospital and Institute	Beijing, China	assistant	LASRE Trial Investigators
Yi	Xiao		MD	Division of Colorectal Surgery, Department of General Surgery, Peking Union Medical College Hospital, Chinese Academy of Medical Sciences & Peking Union Medical College	Beijing, China	assistant	LASRE Trial Investigators
Guo-Le	Lin		MD	Division of Colorectal Surgery, Department of General Surgery, Peking Union Medical College Hospital, Chinese Academy of Medical Sciences & Peking Union Medical College	Beijing, China	assistant	LASRE Trial Investigators
Chao-Yang	Gu		MD	Department of Gastrointestinal Surgery, West China Hospital, Sichuan University	Chengdu, Sichuan, China	assistant	LASRE Trial Investigators
Xiao-Wen	He		MD	Department of Colorectal Surgery, The Sixth Affiliated Hospital, Sun Yat-sen University	Guangzhou, Guandong, China	assistant	LASRE Trial Investigators

Supplemental Online Content: Nonauthor Collaborators
*First name, last name, and suffix (if applicable) are required and will appear in PubMed.

***First Name and Middle Initial(s)**	***Last Name**	***Suffix (eg, Jr, III)**	Academic Degrees	Institution	Location (city, state/province, country)	Role or Contribution, eg, chair, principal investigator	Group (if more than 1 Group listed in the byline) and/or Subgroup (eg, Steering Committee)
Ya-Nan	Wang		MD	Department of General Surgery, Nanfang Hospital, Southern Medical University	Guangzhou, Guandong, China	assistant	LASRE Trial Investigators
Qi-Ken	Li		MD	Department of Colorectal Surgery, Cancer Hospital of the University of Chinese Academy of Sciences & Zhejiang Cancer Hospital	Hangzhou, Zhejiang, China	assistant	LASRE Trial Investigators
Rui	Guo		MD	Department of Colorectal Surgery, Cancer Hospital of China Medical University, Liaoning Cancer Hospital & Institute	Shenyang, Liaoning, China	assistant	LASRE Trial Investigators
Da	Teng		MD	Department of General Surgery, General Hospital of PLA	Beijing, China	assistant	LASRE Trial Investigators
Yu-Gang	Yang		MD	Department of Colorectal & Anal Surgery, Zhangzhou Affiliated Hospital, Fujian Medical University	Zhangzhou, Fujian, China	assistant	LASRE Trial Investigators
Shao-Lan	Qin		MD	Department of Gastrointestinal Surgery, Renji Hospital, Shanghai Jiao Tong University School of Medicine	Shanghai, China	assistant	LASRE Trial Investigators
Jian-An	Lin		MD	Department of Gastrointestinal Surgery, The Second Affiliated Hospital, Fujian Medical University	Quanzhou, Fujian, China	assistant	LASRE Trial Investigators

Supplemental Online Content: Nonauthor Collaborators
*First name, last name, and suffix (if applicable) are required and will appear in PubMed.

***First Name and Middle Initial(s)**	***Last Name**	***Suffix (eg, Jr, III)**	Academic Degrees	Institution	Location (city, state/province, country)	Role or Contribution, eg, chair, principal investigator	Group (if more than 1 Group listed in the byline) and/or Subgroup (eg, Steering Committee)
Qing-Qi	Hong		MD	Department of Gastrointestinal Oncological Surgery, The First Affiliated Hospital, Xiamen University	Xiamen, Fujian, China	assistant	LASRE Trial Investigators
Wen-Feng	Li		MD	Department of Colorectal & Anal Surgery, Longyan Affiliated Hospital, Fujian Medical University	Longyan, Fujian, China	assistant	LASRE Trial Investigators
Lei	Liang		MD	Department of Colorectal Surgery, Fudan University Cancer Center	Shanghai, China	assistant	LASRE Trial Investigators
Jun-Jie	Hu		MD	Department of Gastrointestinal Surgery, Hubei Provincial Cancer Hospital	Wuhan, Hubei, China	assistant	LASRE Trial Investigators
Xing-Hua	Liu		MD	Department of Gastrointestinal Surgery, Union Hospital, Tongji Medical College, Huazhong University of Science and Technology	Wuhan, Hubei, China	assistant	LASRE Trial Investigators
Jun	Li		MD	Department of Colorectal Surgery and Oncology, Key Laboratory of Cancer Prevention and Intervention, Ministry of Education, The Second Affiliated Hospital, School of Medicine, Zhejiang University	Hangzhou, Zhejiang, China	assistant	LASRE Trial Investigators
Hong	Zhang		MD	Department of Colorectal Oncological Surgery, Shengjing Hospital, China Medical University	Shenyang, Liaoning, China	assistant	LASRE Trial Investigators
