# Supplementary material for: Short-term Outcomes of Laparoscopy-Assisted vs Open Surgery for Patients With Low Rectal Cancer: The LASRE Randomized Clinical Trial
Source: JAMA Oncol. 2022 Sep 15;8(11):1607–15. doi: 10.1001/jamaoncol.2022.4079 (PMC9478880; doi:10.1001/jamaoncol.2022.4079)
Supplement: Supplement 4. — Data Sharing Statement [file jamaoncol-e224079-s004.pdf]

Data Sharing Statement

Jiang. Short-Term Outcomes of Laparoscopy-Assisted vs Open Surgery for Patients With Low Rectal Cancer. *JAMA Oncol*. Published September 15, 2022.
doi:10.1001/jamaoncol.2022.4079

Data
Data available: No

Additional Information
Explanation for why data not available: Our data is not yet available to share with others. We are planning to collate and analyze the long-term outcomes and the quality of life and sexual, bladder, and anal function.
